# Engineered CRISPR-Cas12a for higher-order combinatorial chromatin perturbations

**DOI:** 10.1038/s41587-024-02224-0

**Published:** 2024-05-17

**Authors:** C. C.-S. Hsiung, C. M. Wilson, N. A. Sambold, R. Dai, Q. Chen, N. Teyssier, S. Misiukiewicz, A. Arab, T. O’Loughlin, J. C. Cofsky, J. Shi, L. A. Gilbert

**Affiliations:** 1 Department of Pathology, Stanford University School of Medicine, Stanford, CA USA; 2 Department of Urology, University of California, San Francisco, CA USA; 3 Helen Diller Family Comprehensive Cancer Center, University of California, San Francisco, San Francisco, CA USA; 4 Arc Institute, Palo Alto, CA USA; 5 Tetrad Graduate Program, University of California, San Francisco, CA USA; 6 Biomedical Sciences Graduate Program, University of California, San Francisco, San Francisco, CA USA; 7 Department of Cancer Biology, University of Pennsylvania, Philadelphia, PA USA; 8 Biological and Medical Informatics Graduate Program, University of California, San Francisco, San Francisco, CA USA; 9 Department of Biological Chemistry and Molecular Pharmacology, Harvard Medical School, Boston, MA USA

**Keywords:** Functional genomics, Gene regulation, Synthetic biology

## Abstract

Multiplexed genetic perturbations are critical for testing functional interactions among coding or non-coding genetic elements. Compared to double-stranded DNA cutting, repressive chromatin formation using CRISPR interference (CRISPRi) avoids genotoxicity and is more effective for perturbing non-coding regulatory elements in pooled assays. However, current CRISPRi pooled screening approaches are limited to targeting one to three genomic sites per cell. We engineer an *Acidaminococcus* Cas12a (AsCas12a) variant, multiplexed transcriptional interference AsCas12a (multiAsCas12a), that incorporates R1226A, a mutation that stabilizes the ribonucleoprotein–DNA complex via DNA nicking. The multiAsCas12a-KRAB fusion improves CRISPRi activity over DNase-dead AsCas12a-KRAB fusions, often rescuing the activities of lentivirally delivered CRISPR RNAs (crRNA) that are inactive when used with the latter. multiAsCas12a-KRAB supports CRISPRi using 6-plex crRNA arrays in high-throughput pooled screens. Using multiAsCas12a-KRAB, we discover enhancer elements and dissect the combinatorial function of *cis*-regulatory elements in human cells. These results instantiate a group testing framework for efficiently surveying numerous combinations of chromatin perturbations for biological discovery and engineering.

## Abstract (web summary)

An engineered Cas12a enables higher-order combinatorial functional genomic screens using CRISPR interference.

## Main

Functional interactions among combinations of genetic elements underlie many natural and engineered phenotypes^1–3^, often involving higher-order (≥ 3-plex) combinations of coding^4,5^ or non-coding elements^6–9^. Experimentally testing higher-order combinations of genetic perturbations has been limited by throughput, with prior systematic analyses primarily performed in yeast^10–14^. In mammalian functional genomics, pooled screens^15–17^ using sequencing readouts have been limited to up to three genetic perturbations per cell when using RNA interference^18^ or CRISPR-Cas9 (ref. ^19^). Further multiplexing in Cas9-based pooled screening is challenging due to increasingly complex cloning schemes for large constructs encoding multiple guides expressed from separate promoters and length-dependent high recombination frequencies of lentiviral guide libraries^20–22^. Conceptually, it also remains unclear how to tractably survey the potentially vast combinatorial spaces for ≥3-plex perturbations.

Cas12a, a member of the type V CRISPR-Cas family, has been proposed as an alternative to Cas9 for genetic perturbations due to enhanced multiplexing capabilities. Cas12a harbors RNase activity, separable from its DNase activity, that can process a compact primary transcript expressed from a single promoter into multiple CRISPR RNAs (crRNAs)^23,24^. An array of multiple Cas12a crRNAs, each composed of a 19-nt direct repeat and a 19-to 23-nt spacer, can be encoded by a chemically synthesized oligo for single-step cloning into an expression vector^25–29^. Cas12a has been engineered for mammalian cell applications using its DNase activity to disrupt coding gene function using single or multiplexed crRNA constructs in individual well-based assays^24–27,30,31^ and in pooled sequencing screens^28,29,31–35^. However, extended multiplexing with fully DNase-competent Cas12a is expected to be constrained by genotoxicity from double-stranded DNA breaks in many biological contexts^28,36–41^. In principle, avoiding genotoxicity can be achieved by using DNase-dead Cas fusion proteins to control chromatin state and transcription, such as by DNase-dead Cas9 (dCas9)-based CRISPR interference (CRISPRi) or CRISPR activation (CRISPRa)^42–44^. Moreover, CRISPRi is more efficient than DNA cutting at perturbing enhancers in pooled screens^45–47^, likely due to CRISPRi’s larger genomic window of activity via formation of repressive chromatin^48^. Thus, a DNase-dead Cas12a (dCas12a) functional genomics platform for multisite CRISPRi targeting would be highly desirable for testing the combinatorial functions of coding and non-coding genetic elements. However, no dCas12a-based pooled CRISPRi screening platform has been reported. Several studies have used dCas12a fusion proteins for CRISPRi in human cells in individual well-based assays, reporting either successful^27,49–51^ or unsuccessful^52^ repression of target genes. These dCas12a CRISPRi studies delivered crRNA plasmids by transient transfection rather than lentiviral transduction. Transient plasmid transfections express synthetic components at 10- to 1,000-fold higher than single-copy lentiviral integration of crRNA constructs, which is required in pooled screens to attribute cellular phenotypes to unique crRNA constructs by high-throughput sequencing^15,16^. Whether prior dCas12a CRISPRi constructs are sufficiently potent for pooled screens remains unclear.

In this study, we show that existing dCas12a CRISPRi fusion constructs function poorly when used with limiting doses of lentivirally delivered components, thus precluding their application in pooled screens. We engineered an *Acidaminococcus* Cas12a (AsCas12a) variant that incorporates a key mutation, R1226A, which enhances stability of the ribonucleoprotein–DNA complex in the form of a nicked DNA intermediate in vitro^53,54^. We show that in human cells, multiAsCas12a-KRAB fusion substantially improves CRISPRi activity in the setting of lentivirally delivered crRNA constructs, enabling use of 6-plex crRNA arrays in high-throughput pooled screens and up to 10-plex crRNA arrays in well-based assays. We use this combinatorial CRISPRi platform to efficiently discover enhancer elements and to test higher-order combinatorial perturbations of *cis*-regulatory elements. These results instantiate a group testing framework that enables efficient searches of potentially large combinatorial spaces of chromatin perturbations.

## Results

### Lentivirally delivered CRISPRi by dAsCas12a fusion proteins is hypoactive

**Fig. 1:**
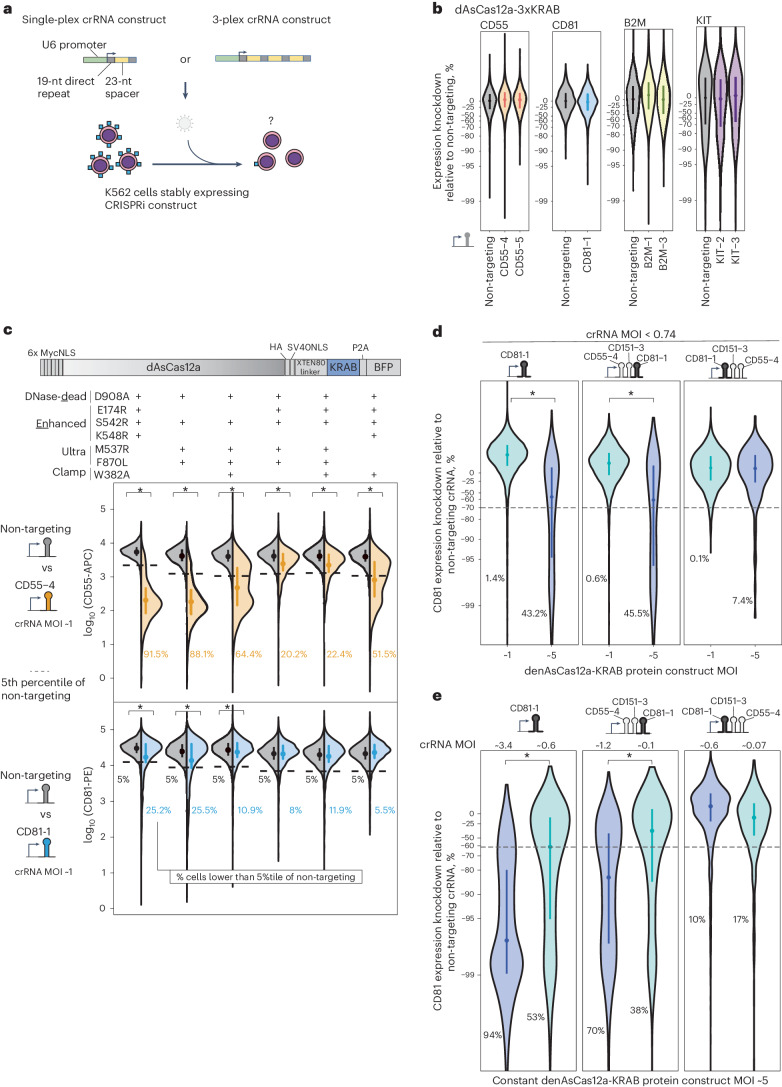
dAsCas12a-KRAB variants are dose-limited and weak in CRISPRi activity when using lentivirally delivered crRNAs, despite incorporating state-of-the-art optimizations. **a**, Schematic for assaying CRISPRi activity of AsCas12a constructs using lentivirally transduced single-plex or 3-plex crRNAs targeting cell surface marker genes assayed by antibody staining and flow cytometry. **b**, K562 cells constitutively expressing dAsCas12a-3xKRAB^27^ were lentivirally transduced with the indicated single crRNAs and assayed by flow cytometry 6 days after crRNA transduction. One of two biological replicates is shown; second replicate is shown in Supplementary Fig. 2. **c**, A panel of AsCas12a variants harboring combinations of mutations are tested using crCD55-4 and crCD81-1 using the fusion protein domain architecture shown. Both AsCas12a fusion protein and crRNA constructs are delivered by lentiviral transduction. D908A is a mutation in the RuvC catalytic triad that renders Cas12a DNase inactive^24,54^. Other mutations are described in detail in the main text. Shown are single-cell distributions of target gene expression assayed by flow cytometry 6 days after crRNA transduction for one of three independent replicates. Additional replicates and results for additional crRNA constructs (up to 3-plex crRNA constructs) are summarized in Supplementary Fig. 6a–c. **d**, Analysis of CD81 knockdown in cells lentivirally transduced with denAsCas12a-KRAB protein construct at multiplicity of infection (MOI) ~1 versus MOI ~5 while maintaining constant crRNA MOI (<0.74) for each crRNA construct. CD81 expression was assayed by flow cytometry 6 days after crRNA transduction. Shown are single-cell distributions for one out of 3–6 biological replicates for each crRNA construct. Summaries of all replicates shown in Supplementary Fig. 7a. **e**, Analysis of CD81 knockdown in cells lentivirally transduced with denAsCas12a-KRAB protein construct at MOI ~ 5, while crRNA MOI is changed from high to low as indicated. CD81 expression was assayed by flow cytometry 10 days after crRNA transduction. Shown are single-cell distributions of CD81 knockdown for one of two biological replicates. Second replicate shown in Supplementary Fig. 7b. **b**–**e**, Medians and interquartile ranges are shown for single-cell distributions (*n* > 200 cells per replicate). **c**–**e**, Asterisks indicate *P* < 0.01 for comparing the replicate-level single-cell distributions of the paired conditions by one-sided Wilcoxon rank-sum test. Percentages of cells below the 5th percentile (dashed line) of non-targeting crRNA are also shown. Source data

We focused on building a CRISPRi functional genomics platform using AsCas12a, the only Cas12a ortholog with demonstrated success in pooled screens in mammalian cells^28,29,31–34,55,56^. A previous study reported using dAsCas12a for CRISPRi by plasmid transient transfection delivery of dAsCas12a-3xKRAB protein (harboring the E993A DNase-dead mutation) and crRNA in HEK 293T cells^27^. To test this construct in the setting of lentivirally delivered crRNA, we introduced dAsCas12a-3xKRAB by piggyBac transposition in K562 cells, followed by lentiviral transduction of single crRNA constructs targeting canonical (TTTV) or non-canonical^30^ protospacer adjacent motifs (PAMs) proximal to transcriptional start sites of four genes encoding for cell surface proteins, three of which (CD55, CD81 and B2M) have been successfully knocked down by dCas9-KRAB CRISPRi (Fig. 1a)^57^. Throughout this study we encoded crRNAs in a CROP-seq^58^ lentiviral vector previously optimized for pooled screens using DNase-active AsCas12a (ref. ^29^). We observed no expression change in any of the targeted genes (Fig. 1b and Supplementary Figs. 1 and 2). We confirmed the expression of dAsCas12a-3xKRAB by western blot (Supplementary Fig. 3) and by flow cytometry monitoring of the in-frame P2A-BFP (Supplementary Fig. 4a). We also observed this lack of CRISPRi activity for dAsCas12a-3xKRAB using lentivirally transduced crRNAs in C4-2B prostate cancer cells (Supplementary Fig. 4b). In contrast, transient co-transfection of dAsCas12a-3xKRAB and CD55-targeting crRNA plasmids shows modest CRISPRi knockdown in HEK 293T cells (Supplementary Fig. 5), consistent with prior work^27^. These findings indicate that the requirements for CRISPRi activity using dAsCas12a-3xKRAB with lentiviral crRNA constructs are distinct from those of plasmid transient transfection in HEK 293T cells^27^.

In an attempt to overcome this lack of CRISPRi activity, we tested combinations of several mutations representing state-of-the-art optimizations of Cas12a. These include 1) E174R/S542R/K548R (enhanced AsCas12a, or enAsCas12a)^30^; 2) M537R/F870L (AsCas12a ultra)^58^; and 3) W382A, a mutation that reduces R-loop dissociation in vitro for an orthologous enzyme (*Lachnospiraceae* Cas12a W355A)^59^ but has not yet been tested in cells. We generated six dAsCas12a variants that each harbor the DNase-inactivating D908A mutation, plus a select combination of the aforementioned mutations. We delivered these variants in K562 cells by stable lentiviral expression, followed by lentiviral transduction of crRNA construct targeting the transcription start site (TSS) of *CD55* or *CD81* (Fig. 1c and Supplementary Fig. 6a,b). Among this panel, denAsCas12a-KRAB (E174R/S542R/K548R, plus D908A DNase-dead mutation) performed the best and demonstrated strong repression of CD55. However, even for this best construct, we observed weak repression of CD81, indicating inconsistent performance across crRNAs (Fig. 1c and Supplementary Fig. 6c).

Dose-response and construct potency are key considerations for multiplexed applications, as increased multiplexing effectively reduces the Cas protein available to bind each individual crRNA. Focusing on denAsCas12a-KRAB as the top variant, we tested the effect of separately altering the dosage of AsCas12a fusion protein and crRNAs. We found that increasing the MOI of the denAsCas12a-KRAB construct from ~1 to ~5 can improve CRISPRi knockdown by some crRNA constructs (Fig. 1d and Supplementary Fig. 7a). However, CRISPRi activity of denAsCas12a-KRAB is reduced or lost when the crRNA MOI is reduced to <1 to mimic the low MOI required for pooled screens (Fig. 1e and Supplementary Fig. 7b). More problematically, CD81 knockdown by a 3-plex crRNA (crCD81-1_crCD151-3_crCD55-4) is extremely weak (~0–25% median expression knockdown relative to non-targeting control) across all doses of protein (Fig. 1d and Supplementary Fig. 7a) and crRNA (Fig. 1e and Supplementary Fig. 7b) tested.

Given the inconsistent and deficient performance of denAsCas12a-KRAB, we tested an alternative CRISPRi approach without mutating the RuvC DNase active site. In the setting of transient plasmid transfection delivery in HEK 293T cells, wild-type AsCas12a has been used for transcriptional control with truncated (15 nt) crRNA spacers, which enable DNA binding but not cleavage^26,27^. We tested this approach by fusing KRAB or 3xKRAB to opAsCas12a, a fully DNase active AsCas12a optimized for pooled screens^29^. We confirmed that 15-nt spacers do not support DNA cleavage, whereas 23-nt spacers do (Supplementary Fig. 8a). However, using 15-nt spacers, we observed weak or no CRISPRi activity in two cell lines (Supplementary Fig. 8b–f). In total, we tested three separate approaches to abolish the DNase activity of AsCas12a (E993A in dAsCas12a-3xKRAB in Fig. 1b and Supplementary Fig. 4b; D908A in Fig. 1c–e and Supplementary Fig. 7a,b; and truncated spacers in Supplementary Fig. 8b–f) that show overall weak and inconsistent CRISPRi activity using lentivirally transduced crRNA constructs.

### MultiAsCas12a-KRAB (R1226A/E174R/S542R/K548R) substantially improves lentivirally delivered CRISPRi

**Fig. 2:**
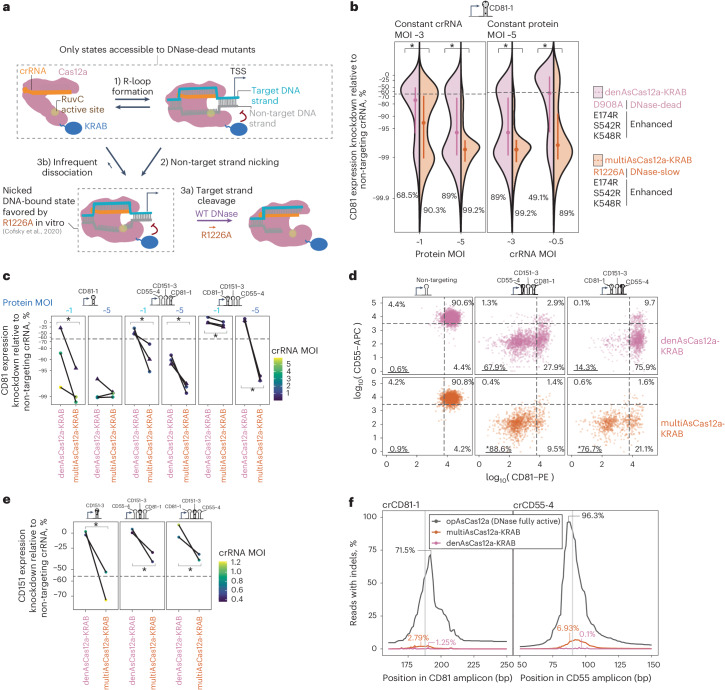
MultiAsCas12a-KRAB (R1226A/E174R/S542R/K548R), an engineered variant that favors a nicked DNA intermediate, substantially improves lentivirally delivered CRISPRi activity. **a**, Model of Cas12a DNA binding and cleavage states for wild-type DNase versus the R1226A nicking-biased mutant based on prior in vitro studies^53,54,60–64^. Sizes of arrows qualitatively reflect relative reaction rates within each biochemical step. **b**, Comparison of denAsCas12a-KRAB (D908A/E174R/S542R/K548R) versus multiAsCas12a-KRAB (R1226A/E174R/S542R/K548R) in CRISPRi knockdown of CD81 expression assayed by flow cytometry 10 days after crRNA transduction, using the combinations of MOI for crRNA and protein shown. One biological replicate is shown for each condition; additional replicates shown in Supplementary Fig. 9. **c**, Comparison of CD81 knockdown by lentivirally delivered denAsCas12a-KRAB versus multiAsCas12a-KRAB at protein MOI ~1 versus ~5 across a panel of single and 3-plex crRNA constructs, maintaining a constant crRNA MOI within each paired comparison of denAsCas12a-KRAB versus multiAsCas12a-KRAB. Lines connect paired experiments within each biological replicate. crRNA MOI indicated by color scale. Dots indicate flow cytometry measurement 10 days after crRNA transduction; triangles indicate measurements 16 days after crRNA transduction. **d**, Same as panel c, but showing scatter plot of CD55-APC and CD81-PE antibody co-staining signals on flow cytometry performed 16 days after transduction of the indicated crRNA constructs in K562 cells lentivirally transduced with denAsCas12a-KRAB versus multiAsCas12a-KRAB at protein MOI ~ 5. One-sided two-sample chi-square test was performed to compare the proportion of cells with double knockdown for denAsCas12a-KRAB versus multiAsCas12a-KRAB for a given crRNA construct (*n* > 200 cells per condition); asterisks indicate *P* < 0.01. **e**, K562 cells piggyBac-engineered to constitutively express denAsCas12a-KRAB or multiAsCas12a-KRAB were transduced with the indicated crRNA constructs, followed by measurement of CD151 expression by antibody staining and flow cytometry 13 days after crRNA transduction. Lines connect paired experiments within each replicate. crRNA MOI indicated by color scale. **f**, Indel quantification from PCR amplicons surrounding target sites of crCD81-1 and crCD55-4 in cells lentivirally transduced at protein MOI ~5 for denAsCas12a-KRAB and multiAsCas12a-KRAB. Cells lentivirally transduced with opAsCas12a (DNase fully active) are shown for comparison. Percent of reads containing indels at each base position within the amplicon is plotted, with labels indicating maximum indel frequency observed across all bases within the amplicon. **b**–**e**, dashed lines indicate 5th percentile of measurements for non-targeting crRNA. Asterisks indicate *P* < 0.01 for one-sided Wilcoxon rank-sum test of single-cell distributions (*n* > 200 cells per replicate) for a given paired comparison for all replicates shown. Source data

The mediocre performance of dAsCas12a for CRISPRi surprised us given the success of AsCas12a in DNA-cutting pooled screens^28,29,34,35^. We wondered whether full inactivation of DNA cutting in dAsCas12a may preclude strong CRISPRi activity by reducing DNA affinity, as previous studies indicate that DNA cleavage strengthens the Cas12a–DNA interaction^53,60,61^. In the Cas12a DNA cleavage process, the RuvC active site first cuts the non-target strand, then the target strand^62^. Although double-strand breaks are undesired for CRISPRi applications, we wondered whether favoring the nicked DNA intermediate might reduce the R-loop dissociation rate (Fig. 2a and Discussion). In support of this possibility, in vitro studies showed that dCas12a–DNA complexes are 20-fold more stable when the non-target strand is precleaved^53^ and that non-target strand nicking biases Cas12a–DNA complexes away from dissociation-prone conformations^63,64^.

To engineer nicking-induced stabilization of AsCas12a binding to DNA for CRISPRi applications, we incorporated R1226A, a mutation that has not been tested in the context of transcriptional control. The R1226A mutant, originally described as a nickase^54^, is ~100- to 1,000-fold slower in cleaving the non-target DNA strand and ~10,000-fold slower in cleaving the target DNA strand in vitro, relative to wild-type AsCas12a (ref. ^53^). Consistent with nicking-induced stabilization, AsCas12a R1226A indeed binds DNA more strongly in vitro than the fully DNase-inactivated D908A variant^53^. We expect the R1226A mutation to both disfavor R-loop reversal and slow progression to double-stranded breaks (Fig. 2a and Discussion). We hypothesized that, by trapping the ribonucleoprotein–DNA in a nicked DNA intermediate, the R1226A mutation would prolong chromatin occupancy and thus the time available for the KRAB domain to recruit transcriptional repressive complexes.

To test the impact of R1226A on CRISPRi activity, we replaced the DNase-inactivating D908A in denAsCas12a-KRAB with R1226A, and we hereafter refer to this Cas12a variant as multiAsCas12a (multiplexed transcriptional interference AsCas12a; that is, R1226A/E174R/S542R/K548R). To compare dose sensitivity of their CRISPRi activities, we stably expressed denAsCas12a-KRAB and multiAsCas12a-KRAB protein and crRNA constructs by lentiviral transduction at high versus low MOIs for protein and crRNA constructs. Across a panel of single and 3-plex crRNA constructs, multiAsCas12a-KRAB consistently exhibits robust CRISPRi with less sensitivity to low MOI of protein or crRNA constructs (Fig. 2b–d and Supplementary Figs. 9 and 10) and shows minimal to no off-target effects on the transcriptome (Supplementary Fig. 11a–c). Notably, multiAsCas12a-KRAB substantially rescues the activities of several crRNA constructs that are virtually inactive for denAsCas12a-KRAB even at high protein dose delivered by either high MOI lentiviral transduction (Fig. 2c,d) or by piggyBac transposition in the setting of a non-canonical GTTC PAM target (crCD151-3; Fig. 2e). Targeting by multiAsCas12a-KRAB results in low indel frequencies at crCD81-1 (2.79%) and crCD55-4 (6.93%) target sites (Fig. 2f). Simulations accounting for DNA copy number^65^ indicate that any possible gene expression impact from indels at these target sites are far lower than the observed target gene knockdown (Supplementary Fig. 14a,b).

### MultiAsCas12a-KRAB enables multigene transcriptional repression using higher-order arrayed crRNA lentiviral constructs

**Fig. 3:**
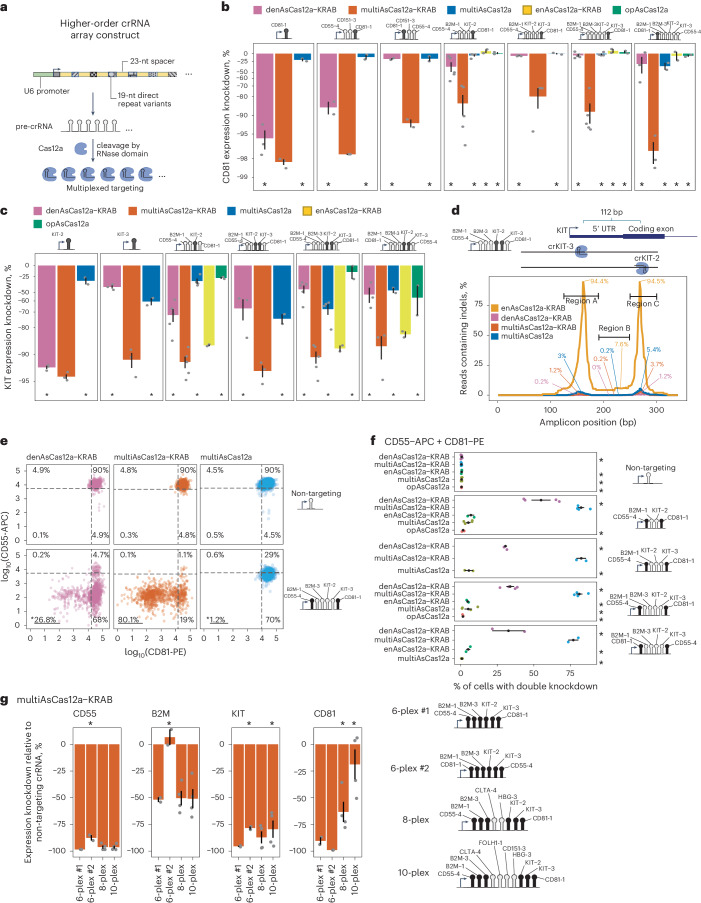
MultiAsCas12a-KRAB enables multigene CRISPRi perturbations using higher-order arrayed crRNA lentiviral constructs. **a**, Schematic for higher-order crRNA expression constructs. **b**–**g**, Experiments were performed on K562 cells engineered by piggyBac transposition of fusion protein constructs (except opAsCas12a was delivered by lentiviral transduction). **b**, Flow cytometry analysis of CD81 expression knockdown 6 days after transduction of the indicated lentiviral crRNA constructs. Shown are averages of median single-cell expression knockdown from two to five biological replicates for each crRNA construct, with error bars indicating standard error of the mean. One-sided Wilcoxon rank-sum test was performed for differences in single-cell expression distributions (*n* > 200 cells) for each fusion protein against multiAsCas12a-KRAB for each replicate. Asterisk indicates *P* < 0.01 for all replicates for a given pairwise comparison. **c**, Same as panel b, but shown for KIT expression knockdown. **d**, Indel quantification by Illumina sequencing of a 340 bp PCR amplicon surrounding two sites on opposite strands near the *KIT* TSS targeted by crKIT-2 and crKIT-3 encoded within a 6-plex crRNA array. Percentages indicates maximum fraction of reads containing indels overlapping any base position within each of the demarcated regions for each of the fusion protein constructs. **e**, Flow cytometry comparison of the indicated fusion protein constructs in dual CD55 and CD81 knockdown 10 days after lentiviral transduction of a 6-plex crRNA construct, shown for one biological replicate. Percentages of cells in each quadrant of the scatter plot, defined by the 5th percentile of non-targeting crRNA for each fluorescence signal, are indicated. One-sided, two-sample chi-square test was used to compare the proportion of cells (*n* = 56–5,006 cells per replicate) with double knockdown between multiAsCas12a-KRAB versus each of the other fusion protein constructs; asterisks indicate *P* < 0.01. **f**, Same analysis as panel **e**, but summarized for additional crRNA constructs and showing the percentage of cells with successful double knockdown of CD55 and CD81. Two to six biological replicates are shown as individual data points and summarized by the mean and standard error of the mean as error bars. **g**, Gene expression knockdown by multiAsCas12a-KRAB using 6-plex, 8-plex and 10-plex crRNA array constructs was measured by flow cytometry 10-11 days after lentiviral transduction of crRNA constructs. Shown are median gene expression knockdown averaged from two to four biological replicates, with error bars denoting standard error of the mean. From the two replicates for which all constructs were tested, one-sided Wilcoxon rank-sum test was performed to compare the single-cell distribution (*n* > 200 cells for each replicate) of the 6-plex #1 construct against each of the other constructs; asterisks indicate *P* < 0.01 for both replicates. Source data

We next tested the performance of multiAsCas12a-KRAB in targeting three or more genomic sites per cell for CRISPRi using lentiviral crRNA arrays^29^. To minimize the possibility of lentiviral recombination, the expression construct (Fig. 3a) uses a unique direct repeat variant at each position of the array, selected from a set of previously tested direct repeat variants^28^. We assembled a panel of 13 distinct crRNA constructs (seven single-plex, two 3-plex, two 4-plex, two 5-plex and two 6-plex) from individually active TSS-targeting spacers (Fig. 3b,c and Supplementary Fig. 12a,b). For these 13 crRNA constructs, we compared the CRISPRi activities of denAsCas12a-KRAB, multiAsCas12a-KRAB and multiAsCas12a (no KRAB). For a subset of crRNA constructs we also added enAsCas12a-KRAB (DNase fully active) as comparison. For these experiments and the remainder of this study we use piggyBac transposition to constitutively express all fusion proteins at very similar levels that are comparable to high MOI (~5) lentiviral fusion protein delivery (Supplementary Figs. 3 and 13a,b), but avoids day-to-day variations in lentiviral titers. Across the entire crRNA panel, multiAsCas12a-KRAB substantially outperforms denAsCas12a-KRAB in CRISPRi activity for seven out of seven constructs targeting *CD81* (Fig. 3b), four out of six constructs targeting *B2M* (Supplementary Fig. 12b) and six out of six constructs targeting *KIT* (Fig. 3c). For CD55 knockdown (Supplementary Fig. 12a), multiCas12a-KRAB substantially outperforms denAsCas12a-KRAB using crCD55-5 (weaker spacer) and performs the same as or marginally better than denAsCas12a-KRAB for seven constructs containing crCD55-4 (stronger spacer). Similarly superior CRISPRi performance by multiAsCas12a-KRAB was observed in C4-2B cells (Supplementary Fig. 4a,b).

For all crRNA constructs tested, multiAsCas12a alone shows much lower impact on target gene expression than multiAsCas12a-KRAB (for example, Fig. 3b for CD81), demonstrating strong dependence of gene knockdown on the KRAB domain. For some target genes, such as *KIT*, partial knockdown can be observed for multiAsCas12a alone (Fig. 3c). Such gene knockdown may be due to 1) non-genetic perturbation of transcription, or 2) alteration of DNA sequences crucial for transcription due to residual DNA cutting. To distinguish these possibilities, we used short-read Illumina sequencing of PCR amplicons (Fig. 3d) to quantify indels generated by the panel of fusion proteins when simultaneously targeting two sites proximal to the *KIT* TSS spaced ~112 bp apart. For multiAsCas12a-KRAB, we observed a maximum indel frequency of 3.7% anywhere in the 340-bp PCR amplicon surrounding these two target sites (Fig. 3d). Based on this observed maximum indel frequency and known DNA copy number in this region^65^, we calculated an upper estimate of expected 1.3% median KIT expression knockdown driven solely by indels, far less than the observed 90.4% median expression knockdown by multiAsCas12a-KRAB, which is 44.4% in excess of the observed for denAsCas12a-KRAB (Supplementary Fig. 15a,b). Similar conclusions are supported by additional measurements at this and other loci obtained for multiAsCas12a-KRAB and/or multiAsCas12a using short-read PCR amplicon sequencing (Supplementary Figs. 14a–d and 15a,b) and/or long-read Nanopore sequencing of native genomic DNA up to tens of kilobases in length (Supplementary Fig. 16a–c). Altogether, our analyses of indel frequencies demonstrate that target gene knockdown by multiAsCas12a-KRAB is largely attributable to nongenetic perturbation of transcription via a combination of direct obstruction of transcription by the Cas protein (as was observed for dCas9 (refs. ^42,43,66^)) and KRAB-mediated repression.

At the single-cell level, multiAsCas12a-KRAB consistently outperforms denAsCas12a-KRAB in the fraction of cells with successful double and triple knockdowns of target genes using higher-order crRNA arrays (Figs. 3e,f and Supplementary Fig. 17a–c). To test the upper limit of multiplexing, we constructed 8-plex and 10-plex constructs assembled using individually active spacers. In these 8-plex and 10-plex arrays, spacers encoded in various positions within the array maintain robust CRISPRi activity (that is, for CD55, KIT and B2M; Fig. 3g). However, crCD81-1 encoded at the 3′ most position in these arrays shows progressive diminishment in CRISPRi activity with further multiplexing at 8-plex and 10-plex (Fig. 3g). This pattern suggests an intrinsic deficiency of crCD81-1 that is unmasked by further multiplexing, perhaps related to the dose sensitivity of this spacer (Fig. 2b,c). Nevertheless, these results indicate that 8-plex and 10-plex crRNA arrays can support robust CRISPRi activity for most spacers within these arrays. We also observed that a specific 6-plex crRNA construct (crCD81-1_crB2M-1_crB2M-3_crKIT-2_crKIT-3_crCD55-4, 6-plex #2 in Fig. 3g) fails to knockdown B2M, despite robust CRISPRi of the other target genes. However, the same combination of spacers in a slightly different 6-plex arrangement (crCD55-4_crB2M-1_crB2M-3_crKIT-2_crKIT-3_crCD81-1) and also in 8-plex and 10-plex embodiments achieve ~50% B2M knockdown (Fig. 3g). These results indicate the existence of still unpredictable pre-crRNA sequence context influences on CRISPRi activity of specific spacers, unrelated to genomic distance from the U6 promoter.

### MultiAsCas12a-KRAB outperforms denAsCas12a-KRAB and performs similarly to dCas9-KRAB in pooled single-guide CRISPRi screens

Given the success of multiAsCas12a-KRAB in individual well-based assays using lentivirally delivered crRNAs, we next evaluated its performance in the context of high-throughput pooled screens. We designed a library, referred to as Library 1 (summarized in Supplementary Fig. 18a), aimed at extracting patterns for Cas12a CRISPRi activity with respect to genomic position relative to the TSS using cell fitness as a readout. Library 1 contains 77,387 single crRNA lentiviral constructs tiling all predicted canonical TTTV PAM sites and non-canonical PAMs (recognizable by enAsCas12a (ref. ^30^)) in the −50-bp to +300 bp region around the TSSs of 559 common essential genes with K562 cell fitness defects in prior genome-wide dCas9-KRAB screens^67^.

**Fig. 4:**
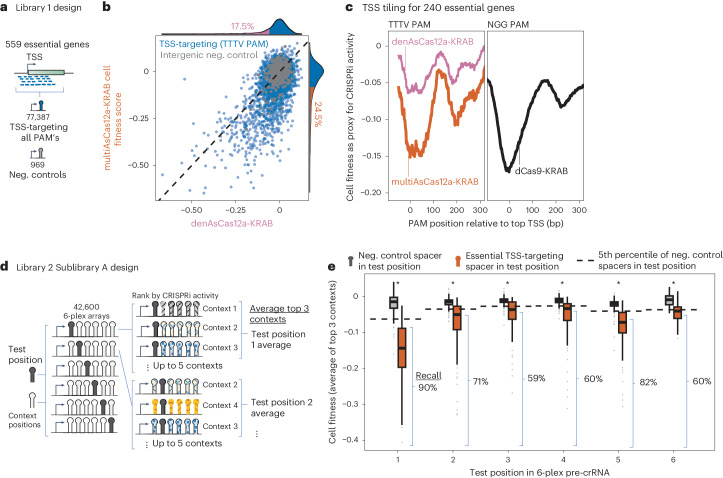
MultiAsCas12a-KRAB enables TSS-targeting pooled CRISPRi screens, including with 6-plex crRNA arrays. **a**, Design of Library 1, consisting of single crRNAs tiling TSS-proximal regions of essential genes. **b**, Library 1: Scatter plot of cell fitness scores in K562 cells for multiAsCas12a-KRAB versus denAsCas12a-KRAB for 3,357 single crRNA constructs with sufficient read coverage for analysis and targeting canonical TTTV PAMs within a −50-bp to +300-bp window of 559 essential gene TSSs. Marginal histograms show percentage of crRNA constructs with cell fitness score is lower than the 5th percentile of negative control crRNAs. **c**, Library 1: Moving average cell fitness score across all TTTV PAM-targeting crRNAs at each PAM position relative to the TSS for denAsCas12a-KRAB or multiAsCas12a-KRAB (left), shown for the 240 essential gene TSS’s for which analogous dCas9-KRAB NGG PAM tiling screen data is available in K562 cells (right)^51^. **d**, Design of Library 2 Sublibrary A, aimed at evaluating CRISPRi activity at each position in the 6-plex array. For each 6-plex array, a specific position is defined as the test position (which can encode either a TSS-targeting spacer or a negative control spacer), and the remaining positions are referred to as context positions encoding one of 5 sets of negative control spacers designated only for context positions. **e**, Library 2 Sublibrary A: Analysis of 2,391 6-plex constructs with sufficient read coverage and that encode in the test position one of 99 spacers that scored as strong hits as single crRNAs in the Library 1 screen (orange boxplots), or negative control crRNA (grey boxplots). Boxplots show cell fitness scores averaged from the top three context constructs of each test position spacer in the 6-plex array from two screen replicates. Recall is calculated as the percentage of essential TSS-targeting spacers (that were empirically active in the single crRNA Library 1 screen) with a cell fitness defect in the Library 2 6-plex crRNA array screen for a given test position, using the 5th percentile of constructs containing negative control spacer in the same test position as a threshold (dashed line) for calling hits. Boxplots display median, interquartile range, whiskers indicating 1.5× interquartile range, and outliers. One-sided Wilcoxon rank-sum test was performed for the difference in the distributions of negative control spacers (*n* = 69–506 constructs) versus TSS-targeting spacers (*n* = 20–99 constructs) at each position, with asterisks indicating *P* < 0.01. Source data

Using K562 cells piggyBac-engineered to constitutively express multiAsCas12a-KRAB or denAsCas12a-KRAB, we conducted a pooled cell fitness screen using this TSS tiling crRNA library transduced at MOI ~0.15. In this assay, CRISPRi knockdown of target essential genes results in the relative depletion of cells harboring the corresponding crRNA over time, quantified as a cell fitness score, negative values of which represent cell fitness defects (Fig. 4a). Concordance between cell fitness scores of screen replicates is high for multiAsCas12a-KRAB (*R* = 0.7) and much lower for denAsCas12a-KRAB (*R* = 0.31), the latter due to much lower signal-to-background ratio (Supplementary Fig. 19a). The cell fitness score distributions are virtually indistinguishable between the intergenic targeting negative controls and the non-targeting negative controls (Supplementary Fig. 20b), indicating no appreciable nonspecific genotoxicity from multiAsCas12a-KRAB single-site targeting. Among the 3,357 crRNAs targeting canonical TTTV PAMs, 24.5% versus 17.5% showed a fitness defect in multiAsCas12a-KRAB versus denAsCas12a-KRAB, respectively (using the 5th percentile of intergenic negative controls as a threshold), with the magnitude of effect for each crRNA overall stronger for multiAsCas12a-KRAB (Fig. 4b).

Previous studies using dCas9-KRAB have identified a strong association between CRISPRi activity and genomic proximity of the crRNA binding site to the TSS^43,51^. We found that multiAsCas12a-KRAB generates a metagene profile of average CRISPRi activity around the TSS that is remarkably similar in magnitude and bimodal genomic distribution of CRISPRi activity obtained by dCas9-KRAB targeting NGG PAMs (Fig. 4c), consistent with nucleosomal hindrance near the +150 bp region downstream of the TSS^51,68^. denAsCas12a-KRAB is substantially weaker than both multiAsCas12a-KRAB and dCas9-KRAB at all positions relative to the TSS (Fig. 4c). multiAsCas12a-KRAB also outperforms denAsCas12a-KRAB in the average CRISPRi activity of the top three best-performing crRNAs/sgRNA for each TSS, and is similar to dCas9-KRAB (Supplementary Fig. 20a). Compared to the canonical TTTV PAMs, a smaller proportion of crRNAs targeting non-canonical PAMs are active, in agreement with lower median CRISPick on-target activity predictions (Supplementary Fig. 21a)^28,69^. Within each PAM sequence, individual crRNAs show significant variations in activity not accounted for by CRISPick predictions (Supplementary Fig. 21b,c).

### MultiAsCas12a-KRAB enables pooled CRISPRi screens using 6-plex crRNA arrays

To evaluate the performance of multiAsCas12a-KRAB in pooled sequencing screens using multiplexed crRNA constructs, we constructed a library consisting of 6-plex crRNAs. We refer to this 6-plex library as Library 2 (summarized in Supplementary Fig. 18b,c), which includes Sublibrary A (described in this section) and Sublibrary B (described in the next section). Sublibrary A was designed to contain 42,600 6-plex constructs for evaluating CRISPRi activity at each of the six positions in the array in a K562 cell fitness screen (Fig. 4d). Each 6-plex construct has one of the six positions designated as the ‘test’ position, which can encode either 1) a spacer targeting one of the top 50 essential gene TSSs (ranked based on prior dCas9-KRAB screen data^51^) or 2) an intergenic negative control (Fig. 4d). The remaining five positions in the array are designated as ‘context’ positions that encode negative control spacers drawn from a separate set of 30 negative control spacers (Fig. 4d). The motivation for this library design was to enable sampling multiple sets of context spacers for a given test position.

The entirety of Library 2 was used in a cell fitness screen in K562 cells piggyBac-engineered to stably express multiAsCas12a-KRAB, conducted with high replicate concordance (*R* = 0.73) (Supplementary Fig. 19b). There is no appreciable non-specific genotoxicity from 6-plex arrays consisting exclusively of intergenic negative control crRNAs (Supplementary Fig. 20c). For a given test position spacer, we calculated the average cell fitness scores from the top three context constructs with the strongest cell fitness defect (Fig. 4d). For each test position, the spacers that previously showed strong cell fitness defects as single crRNAs in the Library 1 screen also showed cell fitness score distributions (average of top 3 contexts) that are clearly lower than the corresponding negative control distributions (Fig. 4e). Each TSS-targeting spacer encoded in the test position elicits a weaker cell fitness defect than the same spacer encoded singly in the Library 1 screen (Supplementary Fig. 22). The recall of empirically active single crRNA spacers from the Library 1 screen by the 6-plex crRNA constructs in this Library 2 Sublibrary A screen ranges from 59% to 90% across test positions (Fig. 4e). As each position in the array is assigned a unique direct repeat variant held constant across all constructs in this analysis, these apparent positional biases may reflect contributions from differences among direct repeat variants. Together, these results systematically demonstrate that the majority of individually active crRNAs retain measurable activities when embedded within 6-plex crRNA arrays in the setting of pooled screens using multiAsCas12a-KRAB.

### Discovery and higher-order combinatorial perturbations of *cis*-regulatory elements

**Fig. 5:**
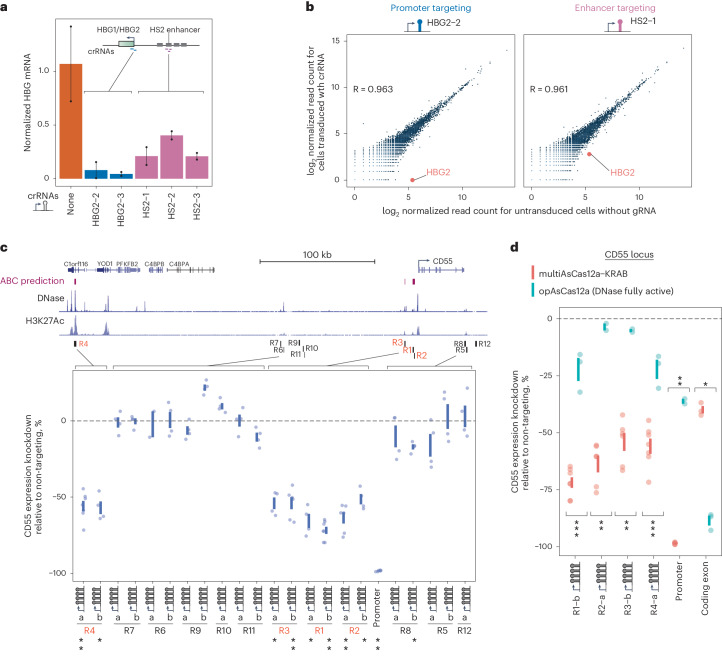
MultiAsCas12a-KRAB CRISPRi enables enhancer perturbation and discovery. **a**, K562 cells lentivirally transduced at MOI ~5 to constitutively express multiAsCas12a-KRAB were lentivirally transduced with single crRNAs targeting the *HBG1/HBG2* TSS’s or their known enhancer, HS2. Shown are *HBG1/HBG2* mRNA levels measured by RT-qPCR, normalized to *GAPDH* mRNA levels for two biological replicates shown as individual data points connected by vertical line to denote the range. **b**, 3’ RNA-seq analysis for a subset of crRNAs shown in panel a. Additional crRNAs and analyses shown in Supplementary Fig. 11a–c. Pearson correlation coefficients are calculated for the transcriptome, excluding *HBG2*. **c**, Genome browser view of the *CD55* locus, including predicted enhancers using the activity-by-contact model^80^ and DNase-seq and H3K27Ac ChIP-seq tracks from ENCODE^110^. K562 cells piggyBac-engineered to constitutively express multiAsCas12a-KRAB were transduced with 4-plex crRNA constructs targeting each candidate region (R1–R12) in the *CD55* locus, with R12 being a negative control region devoid of DNase hypersensitivity and H3K27Ac. For regions targeted by two distinct 4-plex crRNA constructs, each construct is labeled as ‘a’ or ‘b’. For comparison, targeting the *CD55* promoter using a 6-plex crRNA array (crCD55-4_crB2M-1_crKIT-2_crKIT-3_crCD81-1) is included. CD55 expression was assayed by flow cytometry between 9 and 11 days after crRNA transduction. One-sided Wilcoxon rank-sum test was performed on the medians of single-cell expression knockdown across *n* = 2–7 biological replicates (shown as individual data points) for each crRNA construct, compared to the medians of single-cell expression knockdown of R12 (negative control region). **P = 0.01; *P = 0.03. Vertical lines denote standard error of the mean. **d**, Comparison of CRISPRi targeting in K562 cells engineered to constitutively express multiAsCas12a-KRAB (delivered by piggyBac) versus opAsCas12a (delivered by lentiviral transduction) using a subset of lentivirally transduced crRNA constructs from panel b, plus a crRNA construct targeting a coding exon of *CD55* as a positive control for knockdown by DNA cutting. CD55 expression was assayed by flow cytometry 11 days after crRNA transduction. One-sided Wilcoxon rank-sum test was performed to compare the medians of single-cell expression knockdown of multiAsCas12a-KRAB versus opAsCas12a across *n* = 2–7 biological replicates (shown as individual data points, with vertical lines denoting standard error of the mean). ***P = 0.008; **P = 0.033–0.036; *P = 0.05. Source data

The human genome contains ~500,000 predicted enhancers^70^, a small minority of which have been functionally tested by perturbations. To our knowledge, no study has reported enhancer perturbation by CRISPRi using Cas12a. We confirmed that multiAsCas12a-KRAB targeting using single crRNAs can effectively perturb the promoters of the *HBG1/HBG2* paralogs and their known enhancer, HS2 (refs. ^71,72^) (Fig. 5a,b and Supplementary Fig. 11a,b), similar to dCas9-KRAB^73,74^. We next used multiAsCas12a-KRAB to discover previously uncharacterized enhancers using the *CD55* locus in K562 cells as a model. *CD55* encodes for decay-accelerating factor, a cell surface protein that inhibits the activation of complement and is expressed in most human cell types^75^. CD55 function in the myeloid lineage is relevant in multiple disease states, including paroxysmal nocturnal hemoglobinuria^76^ and malaria^77,78^. To our knowledge, no known enhancers in myeloid cells have been identified for *CD55*. In K562 cells, a myeloid cell model, several DNase hypersensitive sites (DHSs) marked by histone 3 lysine 27 acetylation (H3K27Ac), a modification associated with active enhancers^70,79^, reside near *CD55* (Fig. 5c). To conduct a well-based flow cytometry screen of the DHSs within this general region for enhancers that regulate *CD55*, we designed 21 4-plex crRNAs (encompassing 88 unique spacers) targeting 11 manually selected regions (R1–R11) bearing varying levels of DNase hypersensitivity and H3K27Ac, plus a negative control region (R12) devoid of DHSs and H3K27Ac. R1–R4 are predicted by the activity-by-contact (ABC) model^80^ as candidate enhancers. Each region is independently targeted by two completely distinct 4-plex crRNAs (except R10 and R12, which are each targeted by one 4-plex crRNA). We observed ~50%-75% reduction in CD55 expression upon multiAsCas12a CRISPRi targeting of the ABC-predicted R1–R4, whereas no decrease in CD55 expression is observed for R5-R12. For each region, the two distinct 4-plex crRNA arrays show quantitatively similar levels of CD55 knockdown (Fig. 5c), indicating each array contains some 4-plex or lower-order combination of active spacers. This consistency in the magnitude of CD55 expression knockdown likely reflects the magnitude of true enhancer impact on gene transcription, rather than technical peculiarities of individual spacer activities, which might be more unpredictably variable and labor-intensive to test if encoded as single-plex perturbations. To our knowledge, R1–R4 are the first functionally demonstrated enhancers for *CD55* in a myeloid cell type, in addition to another enhancer recently reported in a B-cell model^81^. In contrast to multiAsCas12a-KRAB, using opAsCas12a to target R1–R4 for DNA cutting using the same 4-plex crRNAs elicits very little or no CD55 expression knockdown, despite potent knockdown by a positive control crRNA targeting a coding exon (Fig. 5d).

**Fig. 6:**
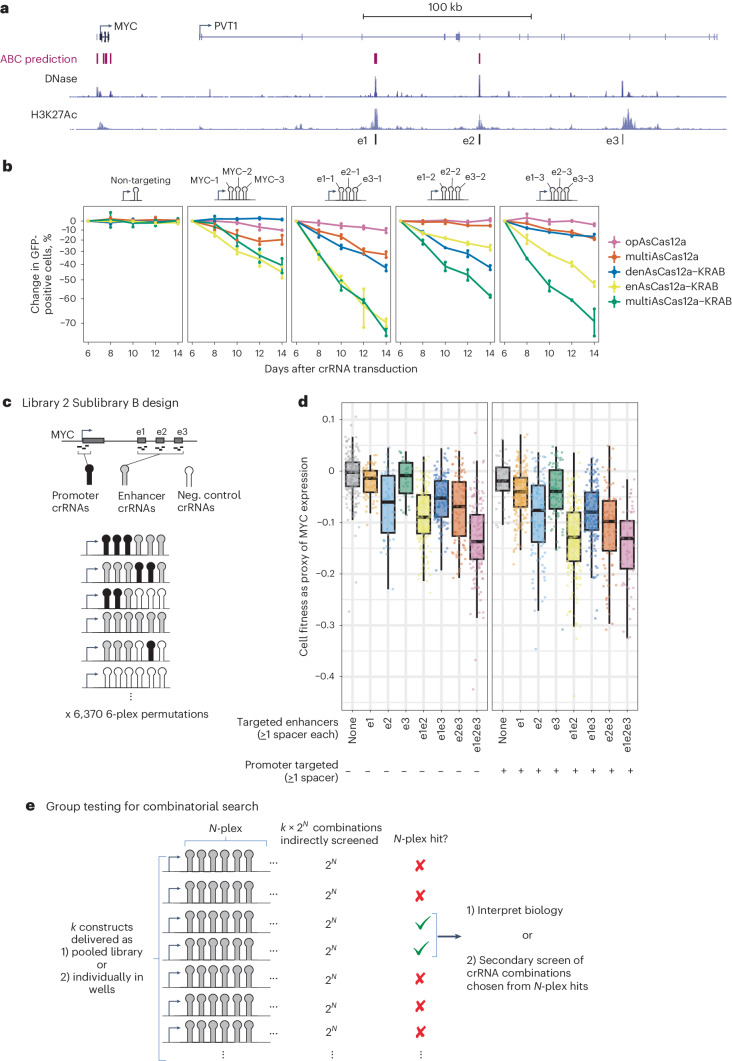
Higher-order combinatorial targeting of *cis*-regulatory elements by multiAsCas12a-KRAB instantiates a group testing framework. **a**, Genome browser view of the *MYC* locus, including activity-by-contact model predictions, and DNase-seq and H3K27Ac ChIP-seq tracks from ENCODE^110^. Three of the known *MYC* enhancers (e1, e2 and e3) in the body of the non-coding RNA, *PVT1*, are shown. **b**, K562 cells piggyBac-engineered to constitutively express the indicated panel of fusion protein constructs were transduced with one of four 3-plex crRNA constructs targeting the *MYC* promoter or cotargeting the three enhancers using one crRNA per enhancer. Cell fitness as a proxy of MYC expression is measured as log_2_ fold-change in percentage of cells expressing GFP marker on the crRNA construct, relative to day 6 after crRNA transduction. The averages of two biological replicates (*n* = 3,432–8,872 cells per replicate) are shown as individual data points and the range denoted by vertical lines. **c**, 6,370 6-plex permutations of the 12 individual spacers from panel b and three intergenic negative control spacers, were designed as 6-plex crRNA arrays in Library 2 Sublibrary B. **d**, Library 2 Sublibrary B: Analysis of 1,629 constructs with sufficient read coverage, categorized based on whether each contains at least one of three crRNAs that target the *MYC* promoter, and/or at least one crRNA that targets each of the *MYC* enhancers. Boxplots summarize cell fitness score distributions (as proxy of MYC expression) of all constructs that fall in each category. Boxplots show median, interquartile range, whiskers indicating 1.5× interquartile range, and are overlaid with individual data points each representing a 6-plex construct. **e**, A general framework for efficiently exploring combinations of CRISPR perturbations using the concept of group testing. Source data

To further test the utility of multiAsCas12a-KRAB in studies of enhancer function, we used the *MYC* locus as a model. Prior studies using CRISPRi pooled screens in K562 cells have shown that MYC expression is proportional to cell fitness^82^ and is regulated by several enhancers identified by screens using cell fitness^82^ and mRNA expression^83^ readouts. A recent study found that pairwise dCas9-KRAB perturbations of these enhancers elicit stronger phenotypes than perturbing single enhancers^84^. Here, we use multiasCas12a-KRAB to dissect the phenotypic impact of ≥3-plex perturbations of *MYC* *cis*-regulatory elements, which remained unknown. To avoid testing intractably numerous higher-order combinations of crRNA spacers that are largely uninformative due to the inclusion of weak or inactive spacers, we pre-screened for a small group of active 3-plex crRNA combinations that can be subsequently assembled into higher and lower-order combinations. We used multiAsCas12a-KRAB to test four 3-plex crRNA constructs each targeting combinations of *MYC* *cis*-regulatory elements (three crRNAs for promoter and three crRNAs for each of three known enhancers: e1, e2 and e3) in a well-based cell competition assay (Fig. 6a,b). We found that these four 3-plex crRNAs induce varying degrees of cell fitness defect as a proxy of MYC expression knockdown, indicating that each construct contains some spacer combination with CRISPRi activity. For comparison, we included denAsCas12a-KRAB, multiAsCas12a, enAsCas12a-KRAB and opAsCas12a, which showed relative activities that further demonstrate that multiAsCas12a-KRAB’s superior gene knockdown is largely attributable to nongenetic transcriptional perturbation (Fig. 6b).

We in silico assembled these 12 nominated spacers and 3 intergenic negative control spacers into Library 2 Sublibrary B, consisting of 6,370 6-plex permutations of these prenominated spacers encoded as 6-plex crRNA arrays (Fig. 6c and summarized in Supplementary Fig. 18c). These 6-plex arrays each target up to 4 *cis*-regulatory elements (promoter + 3 enhancers) with up to three spacers per element. Negative control spacers fill in the remaining array positions not filled by targeting spacers. This Sublibrary B was included as part of the cell fitness screen for the entirety of Library 2. Among 1,629 6-plex arrays with sufficient read coverage for analysis, we grouped them into 16 categories, based on whether it encodes at least one spacer targeting the promoter, and/or at least one spacer targeting each of the three enhancers (Fig. 6d). We found that higher-order targeting of enhancers shows stronger cell fitness defects exceeding that of targeting lower-order enhancer combinations (Fig. 6d, left panel). Cotargeting the promoter together with any combination of enhancers showed greater cell fitness defect over targeting the promoter alone while also exhibiting the cumulative effects of multi-enhancer targeting (Fig. 6d, right panel). These results suggest that when targeting subsets of *cis*-regulatory elements in a locus by CRISPRi, other *cis*-regulatory elements can compete with CRISPRi to partially sustain gene transcription. Such effects may reflect how *cis*-regulatory elements combinatorially respond to endogenous repressive cues in the natural regulation of *MYC* gene transcription. These results demonstrate an example where cotargeting distal *cis*-regulatory elements alone or in combination can be more effective for gene knockdown than targeting the promoter alone. In the Discussion, we explore how the above approaches applied to studying the *cis* regulation of the *CD55* and *MYC* loci instantiate a general group testing framework (Fig. 6e) for efficient testing of combinatorial genetic perturbations.

## Discussion

In this study, we engineered multiAsCas12a-KRAB as a platform for higher-order combinatorial CRISPRi perturbations of gene transcription and enhancer function. The enhanced CRISPRi potency of multiAsCas12a-KRAB is more robust to lower doses of ribonucleoprotein (Figs. 2b,c,e), enabling higher-order multiplexing (Fig. 3a–g) and high-throughput pooled screening applications conducted at single-copy integrations of crRNA (Figs. 4a–e and 6c,d). We propose that the improved CRISPRi activity of multiAsCas12a-KRAB emerges from prolonged chromatin occupancy due to DNA nicking (Fig. 2a). This strategy is conceptually distinct from prior protein engineering approaches to improving Cas12a function in mammalian cells, which focused on substituting for positively charged amino acid residues near the protein–DNA interface^30,49^, using directed evolution to optimize DNA cleavage^58,85^, or optimizing transcriptional effector domain function^56^. We propose the following biophysical explanation for improved function of multiAsCas12a, grounded in prior in vitro literature. In the absence of nicking, R-loop reversal occurs by invasion of the non-target strand into the crRNA:target strand duplex, displacing the crRNA in a process analogous to toehold-mediated nucleic acid strand displacement^86^. Severing the non-target strand increases its conformational entropy and effectively destroys the toehold, decreasing the rate at which the non-target strand can invade the crRNA:target strand duplex^86^. This model can also explain previous observations of cutting-dependent complex stabilization^53,60,61^ and suggests that engineering a nicking preference may improve the efficacy of other Cas enzymes in chromatin targeting. Other potential explanations for multiAsCas12a’s enhanced CRISPRi activity include protein–DNA contacts formed after non-target-strand nicking^59^ and/or nicking-induced relaxation of DNA supercoiling^87^. We have demonstrated that the effects of multiAsCas12a-KRAB’s residual DNase activity on DNA sequence contributes minimally to target gene knockdown for typical functional genomics experimental conditions (Figs. 2f, 3b–f, 5d and 6b and Supplementary Figs. 12 and 14–16). Nevertheless, for screens involving strong positive selection, it may be possible for infrequent deletions to exert more appreciable influence on screen outcome.

We propose that multiAsCas12a-KRAB provides solutions to a major challenge in combinatorial genetics: the infeasibility of surveying potentially enormous combinatorial spaces of ≥3-plex genetic perturbations. Testing a single higher-order *N*-plex combination also indirectly tests all or many of its constituent lower-order combinations, for up to a total of 2^*N*^ combinations. Thus, increases in multiplexing capability potentially yield exponential increases in search efficiency using group testing^88,89^. In group testing (Fig. 6e), a primary screen is conducted on grouped subjects (for example, a multiplexed array of crRNA constructs) to reduce the costs otherwise incurred by individually testing all subjects (for example, individual crRNAs). Our screen for *CD55* enhancers instantiates this approach by testing 22 4-plex crRNA arrays targeting 12 candidate regions, indirectly testing 22 × 2^4^ = 352 crRNA combinations in a cost-effective well-based experiment (Fig. 5c). For this experimental objective, the grouped hits can be biologically interpreted without exhaustively testing the lower-order combinations of crRNAs (Fig. 6e). For other objectives, such as the analysis of combinatorial *cis* regulation at the *MYC* locus, grouped hits (Fig. 6b) can be followed by secondary testing of the combinatorial logic (Fig. 6c,d) as needed. For pooled sequencing screens, the ability to deterministically encode specific higher-order crRNA combinations in a single array is crucial for group testing. In contrast, cloning combinatorial guide libraries by a multiplicative and stochastic approach^19^ requires testing all combinations at the onset and thus is incompatible with group testing. Group testing can significantly compress the size of crRNA libraries to facilitate screens limited by assayable cell numbers. Group testing may also be combined with compressed sensing^90,91^ to facilitate screens with multidimensional phenotypic readouts^92–99^.

A key parameter in group testing is the extent of potential signal dilution and/or interference relative to individual testing. Signal dilution can arise from limiting doses of ribonucleoprotein due to delivery format or reduction in the protein available to bind each individual crRNA due to increased crRNA multiplexing. Signal interference can arise if certain sequence features in one part of the crRNA array masks the activity of individual constituent crRNAs in a dominant fashion. Despite some evidence of signal dilution and/or interference for multiplexed crRNA arrays (Figs. 3g and 4e and Supplementary Fig. 22), multiAsCas12a-KRAB demonstrates sufficient robustness for yielding biological insights into combinatorial *cis* regulation at the *MYC* locus using 6-plex crRNAs in high-throughput pooled screens (Fig. 6c,d). Although we have emphasized meeting the stringent requirements of pooled screening formats, multiAsCas12a also significantly lowers technical barriers to higher-order crRNA perturbations in array-based screening (Fig. 5c), which has recently improved in throughput^100^. The assay format will likely influence the deliverable dose of synthetic components and thus the upper limit of multiplexing for effective CRISPRi using multiAsCas12a-KRAB. Improved prediction of crRNA array activities will likely further support highly multiplexed and/or dose-limited applications, thus extending the scalability of combinatorial genetic screens by group testing. A fully active 10-plex crRNA array could indirectly screen up to 2^10^ = 1,024 crRNA combinations. Another area for improvement is the observation that a low proportion of crRNAs targeting non-canonical PAMs show CRISPRi activity when targeted by multiAsCas12a-KRAB (Supplementary Fig. 21a). Increasing the fraction of active crRNAs, including for non-canonical PAMs, would enable more reliable targeting with fewer crRNAs, especially in GC-rich TSS-proximal regions.

Although we have focused on CRISPRi applications using the KRAB domain, the discovery and engineering of effector domains for chromatin perturbations by CRISPR-Cas is rapidly evolving. Recent advances include repressive effectors^101–104^, activation effectors^56,102,104,105^ and combination effectors for epigenetic memory^51,106–109^. We expect that multiAsCas12a can be flexibly combined with these and other effector domains to support group testing for many chromatin perturbation objectives. We envision multiAsCas12a and the group testing framework will enable engineering and elucidating combinatorial genetic processes underlying broad areas of biology at previously intractable scales.

## Methods

### Plasmid design and construction

A summary of plasmid constructs are in Supplementary Table 1 and plasmid sequences are in Supplementary Data 2. Unless otherwise specified, cloning was performed by Gibson Assembly of PCR-amplified or commercially synthesized gene fragments (from Integrated DNA Technologies or Twist Bioscience) using NEBuilder Hifi Master Mix (NEB, E262), and final plasmids sequence-verified by Sanger sequencing of the open reading frame and/or commercial whole-plasmid sequencing service provided by Primordium.

#### Protein expression constructs

To summarize, denAsCas12a-KRAB, multiAsCas12a-KRAB, multiAsCas12a and enAsCas12a-KRAB open reading frames were embedded in the same fusion protein architecture consisting of an N-terminal 6xMyc-NLS^29^ and C-terminal XTEN80-KRAB-P2A-BFP^103^. The denAsCas12a open reading frame was PCR amplified from pCAG-denAsCas12a(E174R/S542R/K548R/D908A)-NLS(nuc)-3xHA-VPR (RTW776) (Addgene, plasmid 107943 (ref. ^30^)). AsCas12a variants described were generated by using the denAsCas12a open reading frame as starting template and introducing the specific mutations encoded in overhangs on PCR primers that serve as junctions of Gibson assembly reactions. opAsCas12a (ref. ^29^) is available as Addgene plasmid 149723, pRG232. 6xMyc-NLS was PCR amplified from pRG232. KRAB domain sequence from KOX1 was previously reported^42^. The lentiviral backbone for expressing Cas12a fusion protein constructs expresses the transgene from an SFFV promoter adjacent to UCOE and is a gift from Marco Jost and Jonathan Weissman, derived from a plasmid available as Addgene 188765. XTEN80 linker sequence was taken from a previous study^51^ and was originally from Schellenberger et al.^111^. For constructs used in piggyBac transposition, the open reading frame was cloned into a piggyBac vector backbone (Addgene, 133568) and expressed from a CAG promoter. Super PiggyBac Transposase (PB210PA-1) was purchased from System Biosciences.

dAsCas12a-3xKRAB open reading frame sequence is from a construct originally referred to as SiT-ddCas12a-[Repr]^27^. We generated SiT-ddCas12a-[Repr] by introducing the DNase-inactivating E993A by PCR-based mutagenesis using SiT-Cas12a-[Repr] (Addgene, 133568) as template. Using Gibson Assembly of PCR products, we inserted the resulting ddCas12a-[Repr] open reading frame in-frame with P2A-BFP in a piggyBac vector (Addgene, 133568) to enable direct comparison with other fusion protein constructs cloned in the same vector backbone (crRNAs are encoded on separate plasmids as described below).

Fusion protein constructs described in Supplementary Fig. 8b–f were assembled by subcloning the protein-coding sequences of AsCas12a and KRAB into a lentiviral expression vector using the In-Fusion HD Cloning system (TBUSA). AsCas12a mutants were cloned by mutagenesis PCR on the complete wild-type AsCas12a vector to generate the final lentiviral expression vector.

#### crRNA expression constructs

All individually cloned crRNA constructs and their expression vector backbone are listed in Supplementary Table 1. Unless otherwise specified, individual single and 3-plex crRNA constructs were cloned into the human U6 promoter-driven expression vector pRG212 (Addgene, 149722 (ref. ^29^)), which contains wildtype (WT) direct repeats (DR). Library 1, Library 2, and some 3-plex and all 4-plex, 5-plex and 6-plex *As*. crRNA constructs were cloned into pCH67, which is derived from pRG212 by replacing the 3’ DR with the variant DR8 (ref. ^28^). For constructs cloned into pCH67, the specific *As*. DR variants were assigned to each position of the array as follows, in 5′ to 3′ order:

3-plex: WT DR, DR1, DR3, DR8

4-plex: WT DR, DR1, DR10, DR3, DR8

5-plex: WT DR, DR1, DR16, DR10, DR3, DR8

6-plex: WT DR, DR1, DR16, DR18, DR10, DR3, DR8

8-plex: WT DR, DR1, DR16, DR_NS1, DR17, DR18, DR10, DR3, DR8

10-plex: WT DR, DR1, DR16, DR_NS1, DR4, DR_NS2, DR17, DR18, DR10, DR3, DR8

DR sequences are as follows: WT DR = AATTTCTACTCTTGTAGAT, DR1 = AATTTCTACTGTCGTAGAT, DR16 = AATTCCTACTATTGTAGGT, DR_NS1 = AATTCCTCCTCTTGGAGGT, DR4 = AATTTCTACTATTGTAGAT, DR_NS2 = AATTCCTCCTATAGGAGGT, DR17 = AATTTCTCCTATAGGAGAT, DR18 = AATTCCTACTCTAGTAGGT, DR10 = AATTCCTACTCTCGTAGGT, DR3 = AATTTCTACTCTAGTAGAT, DR8 = AATTTCTCCTCTAGGAGAT. Sequences for DR variants were previously reported^28^, except for DR_NS1 and DR_NS2, which were newly designed based on combining previously reported variants^28^. The rationale for selecting specific DR variants was to minimize homology across variants and maintain high crRNA activity based on prior analysis^28^.

1-plex,3-plex, 8-plex, and 10-plex crRNA constructs were cloned by annealing sets of complementary oligos with compatible overhangs in spacer regions, phosphorylation by T4 polynucleotide kinase (NEB M0201S), and ligated with T4 DNA ligase (NEB M0202) into BsmbI site of vector backbones. 4-plex, 5-plex and 6-plex crRNA arrays were ordered as double-stranded gene fragments and cloned into the BsmbI site of vector backbones by Gibson Assembly using the NEBuilder HiFi DNA Assembly Master Mix (NEB, E2621). Functions for designing oligos or gene blocks for cloning crRNA arrays are available as an R package at https://github.com/chris-hsiung/bears01.

### Design of individual crRNAs

All spacer and PAM sequences are provided in Supplementary Table 1. For cloning individual crRNA constructs targeting TSS’s, CRISPick (https://portals.broadinstitute.org/gppx/crispick/public) was used in the enAsCas12a ‘CRISPRi’ mode (by providing gene name) or ‘CRISPRko’ mode (by providing sequence for TSS-proximal regions) to design spacers targeting canonical (TTTV) or non-canonical PAMs generally located within −50-bp to +300-bp region around the targeted TSS whenever possible, but some sites farther from the annotated TSS can show successful CRISPRi activity and were used. We manually selected spacers from the CRISPick output by prioritizing the highest on-target efficacy scores while avoiding spacers with high off-target predictions. The same non-targeting spacer was used throughout the individual well-based experiments and was randomly generated and checked for absence of alignment to the human genome by BLAT^112^.

The hg19 genomic coordinates for *MYC* enhancers are e1 chr8:128910869-128911521, e2 chr8:128972341-128973219 and e3 chr8:129057272-129057795. DNA sequences from those regions were downloaded from the UCSC Genome Browser and submitted to CRISPick. The top three spacers targeting each enhancer were picked based on CRISPick on-target efficacy score, having no Tier I or Tier II Bin I predicted off-target sites, and considering proximity to peaks of ENCODE^110^ DNase hypersensitivity signal (UCSC Genome Browser^113^ accession # wgEncodeEH000484, wgEncodeUwDnaseK562RawRep1.bigWig) and H3K27Ac ChIP-seq signal (UCSC Genome Browser accession # wgEncodeEH000043, wgEncodeBroadHistoneK562H3k27acStdSig.bigWig). These DNase hypersensitivity and H3K27Ac ChIP-seq tracks were similarly used to nominate candidate enhancer regions at the *CD55* locus, whose genomic sequences are provided in Supplementary Table 1.

### Cell culture, lentiviral production, lentiviral transduction and cell line engineering

C4-2B cells^114^ were gifted by F. Feng, originally gifted by L. Chung. All cell lines were cultured at 37°C with 5% CO_2_ in tissue culture incubators. K562 and C4-2B cells were maintained in RPMI-1640 (Gibco, 22400121) containing 25 mM HEPES, 2 mM L-glutamine and supplemented with 10% FBS (VWR), 100 U ml^−1^ streptomycin, and 100 mg ml^−1^ penicillin. For pooled screens using K562 cells cultured in flasks in a shaking incubator, the culture medium was supplemented with 0.1% Pluronic F-127 (Thermo Fisher, P6866). HEK 293T cells were cultured in media consisting of DMEM, high glucose (Gibco 11965084, containing 4.5 g ml^−1^ glucose and 4mM L-glutamine) supplemented with 10% FBS (VWR) and 100 units/mL streptomycin, 100 mg ml^−1^ penicillin. Adherent cells were routinely passaged and harvested by incubation with 0.25% trypsin-EDTA (Thermo Fisher, 25200056) at 37°C for 5–10 min, followed by neutralization with media containing 10% FBS.

Unless otherwise specified below, lentiviral particles were produced by transfecting standard packaging vectors (pMD2.G and pCMV-dR8.91) into HEK293T using TransIT-LT1 Transfection Reagent (Mirus, MIR2306). At <24 h after transfection, culture medium was exchanged with fresh medium supplemented with ViralBoost (Alstem Bio, VB100) at 1:500 dilution. Viral supernatants were harvested ~48–72 h after transfection and filtered through a 0.45 mm PVDF syringe filter and either stored in 4°C for use within <2 weeks or stored in −80°C until use. Lentiviral infections included polybrene (8 µg/ml). MOI was estimated from the fraction of transduced cells (based on fluorescence marker positivity) by the following equation^115,116^: MOI =−ln(1 − fraction of cells transduced).

For experiments described in Supplemental Fig. 8a–f, lentivirus was produced by transfecting HEK293T cells with lentiviral vector, VSVG and psPAX2 helper plasmids using polyethylenimine. Medium was changed ~6–8 h post transfection. Viral supernatant was collected every 12 h five times and passed through 0.45-µm PVDF filters. Lentivirus was added to target cell lines with 8 µg ml^−1^ polybrene and centrifuged at 650 ×*g* for 25 min at room temperature. Medium was replaced 15 h after infection. An antibiotic (1 µg ml^−1^ puromycin) was added 48 h after infection.

For piggyBac transposition of fusion protein constructs, cells were electroporated with ~210 ng of AsCas12a fusion protein plasmid and ~84 ng of Super PiggyBac Transposase Expression Vector (PB210PA-1, Systems Biosciences) using the SF Cell Line 4D-Nucleofector X Kit (V4XC-2032, Lonza Bioscience) and the 4D-Nucleofector X Unit as per manufacturer’s instructions (FF-120 program for K562 cells; EN-120 program for C4-2B cells).

### Antibody staining and flow cytometry

The following antibodies were used for flow cytometry at 1:100 dilution: CD55-APC (BioLegend, 311312), CD55-PE (BioLegend, 311308), CD81-PE (BioLegend, 349506), CD81-AlexaFluor700 (BioLegend, 349518), B2M-APC (BioLegend, 316311), KIT-PE (BioLegend, 313204), KIT-BrilliantViolet785 (BioLegend, 313238) and FOLH1-APC (BioLegend, 342508). Cells were stained with antibodies were diluted in FACS Buffer (PBS with 1% BSA) and washed with FACS Buffer, followed by data acquisition on the Attune NxT instrument in 96-well plate format unless otherwise specified. For CRISPRi experiments, all data points shown in figures are events first gated for single cells based on FSC/SSC, then gated on GFP-positivity as a marker for cells successfully transduced with crRNA construct, as exemplified in Supplementary Fig. 1. For CRISPRi experiments in C4-2B cells, propensity score matching on BFP signal was performed using the MatchIt v4.5.3 R package.

For cell fitness competition assays, the percentage of cells expressing the GFP marker encoded on the crRNA expression vector is quantified by flow cytometry. log_2_ fold-change of percentage of GFP-positive cells was calculated relative to day 2 (for experiments targeting the *Rpa3* locus in Supplementary Fig. 8) or day 6 (for experiments targeting the *MYC* locus in Fig. 6b). For experiments targeting the *Rpa3* locus, flow cytometry was performed on the Guava Easycyte 10 HT instrument.

### Pooled crRNA library design

For all crRNAs in Library 1 and Library 2, we excluded in the analysis spacers with the following off-target prediction criteria using CRISPick run in the CRISPRi setting: 1) off-target match = ‘MAX’ for any tier or bin, or 2) # Off-Target Tier I Match Bin I Matches > 1). The only crRNAs for which this filter was not applied are the non-targeting negative control spacers, which do not have an associated CRISPick output. All crRNA sequences were also filtered to exclude BsmbI sites used for cloning and three or more consecutive T’s, which mimic RNA Pol III termination signal.

#### Library 1 (single crRNAs)

To design crRNA spacers targeting gene TSS’s for Library 1, we used the −50-bp to +300-bp regions of TSS annotations derived from capped analysis of gene expression data and can include multiple TSSs per gene^67^. We targeted the TSSs of 559 common essential genes from DepMap with the strongest cell fitness defects in K562 cells based on prior dCas9-KRAB CRISPRi screen^67^. We used CRISPick with enAsCas12a settings to target all possible PAMs (TTTV and 44 non-canonical PAMs) in these TSS-proximal regions. Except for the criteria mentioned in the previous paragraph, no other exclusion criteria were applied. For the TSS-level analyses shown in Fig. 4d,e, each gene was assigned to a single TSS targeted by the crRNA with the strongest fitness score for that gene.

Negative controls in Library 1 fall into two categories: 1) 524 intergenic negative controls, and 2) 445 non-targeting negative controls that do not map to the human genome. Target sites for intergenic negative controls were picked by removing all regions in the hg19 genome that are within 10 kb of annotated ensembl genes (retrieved from biomaRt from https://grch37.ensembl.org) or within 3 kb of any ENCODE DNase hypersensitive site (wgEncodeRegDnaseClusteredV3.bed from http://hgdownload.cse.ucsc.edu/goldenpath/hg19/encodeDCC/wgEncodeRegDnaseClustered/). The remaining regions were divided into 1-kb fragments. 90 such 1-kb fragments were sampled from each chromosome. Fragments containing ≥20 consecutive Ns were removed. The remaining sequences were submitted to CRISPick run under CRISPRi settings. The CRISPick output was further filtered for spacers that meet these criteria: 1) off-target prediction criteria described in the beginning of this section, and 2) on-target Efficacy Score ≥0.5 (the rationale is to maximize representation by likely active crRNAs to bias for revealing any potential cell fitness effects from nonspecific genotoxicity due to residual DNA cutting by multiCas12a-KRAB), 3) mapping uniquely to the hg19 genome by Bowtie^117^ using ‘-m 1’ and otherwise default parameters, 3) filtered once more against those whose uniquely mapped site falls within 10 kb of annotated ensembl genes or any ENCODE DNase hypersensitive site.

Non-targeting negative control spacers were generated by 1) combining non-targeting negative controls in the Humagne C and D libraries (Addgene accession numbers 172650 and 172651), 2) taking 20-nt non-targeting spacers from the dCas9-KRAB CRISPRi_v2 genome-wide library^67^, removing the G in the 1st position and appending random 4-mers to the 3’ end. This set of spacers were then filtered for those that do not map to the hg19 genome using Bowtie with default settings.

#### Library 2 (6-plex crRNAs)

Sublibrary A (42,600 constructs designed): Test position spacers were encoded at each position of the 6-plex array, with remaining positions referred to as context positions and filled with negative control spacers. Test positions encodes one of 506 intergenic negative control spacers and 914 essential TSS-targeting spacers. The essential TSS-targeting spacers were selected from among all spacers targeting PAMs within −50-bp to +300-bp TSS-proximal regions of 50 common essential genes with the strongest K562 cell fitness defect in prior dCas9-KRAB CRISPRi screen^67^ and must have ≥0.7 CRISPick on-target efficacy score. Negative control context spacers consist of five 6-plex combinations; three of these combinations consist entirely of non-targeting negative controls, and two of the combinations consist entirely of intergenic negative controls.

Sublibrary B (6,370 constructs designed): crRNA combinations targeting *cis*-regulatory elements at the *MYC* locus were assembled from a subset of combinations possible from 15 starting spacers (3 targeting *MYC* TSS, 3 targeting each of 3 enhancers, and 3 intergenic negative control spacers). The three enhancer elements are described in the subsection ‘Design of individual crRNAs.’ These 15 starting spacers were grouped into 5 3-plex combinations, each 3-plex combination exclusively targeting one of the four *cis*-regulatory elements, or consisting entirely of intergenic negative controls. Each 3-plex was then encoded in positions 1–3 of 6-plex arrays, and positions 4–6 were filled with all possible 3-plex combinations chosen from the starting 15 spacers. All 6-plex combinations were also encoded in the reverse order in the array.

All-negative control constructs (2,000 constructs designed): 1,500 6-plex combinations were randomly sampled from the intergenic negative control spacers described for Library 1. 500 6-plex combinations were randomly sampled from non-targeting negative control spacers described for Library 1.

Intergenic negative controls and non-targeting negative controls are defined the same as in Library 1.

As Library 2 was designed and cloned prior to the completion of the Library 1 screen, the majority of Library 2 contains constructs encoding for spacers in the test position that in hindsight do not produce strong phenotypes as single crRNAs in the Library 1 screen.

Both Library 1 and Library 2 were constructed from pooled oligonucleotide libraries designed to contain crRNA constructs designed for exploratory analysis for a separate unpublished study. Sequencing reads from those non-contributory constructs are present in the raw fastq files, do not affect interpretation of Library 1 and Library 2 screen cell fitness scores, and are excluded from analysis in the present study.

### crRNA library construction

All PCRs were performed with NEBNext Ultra II Q5 Master Mix (NEB M0544). For Library 1, ~140 fmol pooled oligo libraries from Twist were subjected to 10 cycles of PCR amplification using primers specific to adaptor sequences flanking the oligos and containing BsmbI sites. The PCR amplicons were cloned into a crRNA expression backbone (pCH67) by Golden Gate Assembly with ~1:1 insert:backbone ratio using ~500 fmol, followed by bacterial transformation to arrive at an estimated 778× coverage in the final plasmid Library 1. For Library 2, 915 fmol of pooled oligo libraries from Twist was subjected to 18 cycles of PCR amplification and agarose gel purification of the correctly sized band before proceeding to Golden Gate Assembly. The estimated coverage of plasmid Library 2 from bacterial colony forming units is ~60×. Additional details are described in Supplementary Information.

### Illumina sequencing library preparation

Primer sequences are provided in Supplementary Table 2. Sequences of the expected PCR amplicons for Illumina sequencing are in Supplementary Data 2. crRNA inserts were amplified from genomic DNA isolated from screens using 16 cycles of first round PCR using pooled 0-8nt staggered forward and reverse primers, treated with ExoSAP-IT (Thermo Fisher, 78201.1.ML), followed by 7 cycles of round 2 PCR to introduce Illumina unique dual indices and adaptors. Sequencing primer binding sites, unique dual indices, P5 and P7 adaptor sequences are from Illumina Adaptor Sequences Document #1000000002694 v16. PCR amplicons were subject to size selection by magnetic beads (SPRIselect, Beckman, B23318) prior to sequencing on an Illumina NovaSeq6000 using SP100 kit (PE100) for Library 1 or SP500 kit (PE250) for Library 2. Sequencing of plasmid libraries were performed similarly, except 7 cycles of amplification were each used for Round 1 and Round 2 PCR. The size distribution of the final library was measured on an Agilent TapeStation system. We noted that even after magnetic bead selection of Round 2 PCR-amplified Library 2 plasmid library (colonies from which were Sanger sequencing verified) and genomic DNA from screens, smaller sized fragments from PCR amplification during Illumina sequencing library preparation persisted. Thus, the majority of unmapped reads likely reflect undesired PCR by-products, though lentiviral recombination could contribute at an uncertain but relatively low frequency as well.

### Cell fitness screens

Library 1 screen: K562 cells engineered by piggyBac transposition to constitutively express denAsCas12a-KRAB or multiAsCas12a-KRAB were transduced with lentivirally packaged Library 1 constructs at MOI ~0.15. Transduced cells were then selected using 1 µg/ml puromycin for 2 days, followed by washout of puromycin. On Day 6 after transduction, initial (T0) time point was harvested, and the culture was split into 2 replicates that are separately cultured henceforth. 10 days later (T10), the final time point was harvested (8.6 total doublings for multiAsCas12a-KRAB cells, 9.15 total doublings for denasCas12a-KRAB cells). A cell coverage of >500× was maintained throughout the screen. Library 2 screen: K562 cells engineered by piggyBac transposition to constitutively express multiAsCas12a-KRAB were transduced with lentivirally packaged Library 2 constructs at MOI ~0.15. The screen was carried out similarly as described for Library 1 screen, except the screen was carried out for 14 days (T14) or 13.5 total doublings and maintained at a cell coverage of >2,000× throughout. Genomic DNA was isolated using the NucleoSpin Blood XL Maxi kit (Machery-Nagel, 740950.50).

### Screen data processing and analysis

Summary of library contents are in Supplementary Fig. 18.

For Library 1, reads were mapped to crRNA constructs using sgcount (https://noamteyssier.github.io/sgcount/), requiring perfect match to the reference sequence. For Library 2, reads were mapped using an algorithm (detailed in Supplementary Information) requiring perfect match to the reference sequence, implemented as ‘casmap constructs‘ command in a package written in Rust, available at https://github.com/noamteyssier/casmap.

Starting from read counts, the remainder of analyses were performed using custom scripts in R. Constructs that contained less than 1 reads per million (RPM) aligned to the reference library in either replicates at T0 were removed from analysis. From the constructs that meet this read coverage threshold, a pseudocount of 1 was added for each construct and the RPM recalculated and used to obtain a fitness score^118^ that can be interpreted as the fractional defect in cell fitness per cell population doubling:

$${\gamma }=\log_2\left(\frac{\left({\mathrm{RPMfinal}}/{\mathrm{negctrlmedianRPMfinal}}\right)}{\left({\mathrm{RPMinitial}}/{\mathrm{negctrlmedianRPMinitial}}\right)}\right)\Big/{\mathrm{totaldoublings}},$$

where RPM is the read count per million reads mapped to reference (initial = at T0, final = at end of screen), negctrlmedian is the median of RPM of intergenic negative control constructs, totaldoublings is the total cell population doublings in the screen. For Library 1, data from a single T0 sample was used to calculate the fitness score for both replicates due to an unexpected global loss of sequencing read counts for one of two originally intended T0 replicate samples. For each screen replicate in Library 2, data from two separate sequencing library preps from the same Round 1 PCR material subjected to separate Round 2 PCRs and sequenced on separate runs were pooled together for analysis.

### Indel analysis by Illumina short-read sequencing

K562 cell lines engineered with the corresponding Cas12a protein constructs were transduced with crRNAs and sorted for transduced cells based on GFP-positivity. 200,000 cells were collected 14 or 15 days after crRNA transduction and genomic DNA was isolated using NucleoSpin Blood (Macherey-Nagel, 740951.50). For analysis of *CD55* and *CD81* loci, PCRs for loci of interest were run using Amplicon-EZ (Genewiz) partial Illumina adapters and amplicons were processed using NucleoSpin Gel and PCR Clean-up Kit (Macherey-Nagel, 740609.250). Paired-end (2 × 250 bp) sequencing was completed at GENEWIZ (Azenta Life Sciences). Raw fastq files were obtained from GENEWIZ and aligned to reference sequences using *CRISPResso2* (ref. ^119^). Quantification diagrams were generated in R. For analysis at the *KIT* locus, cells were lysed using QuickExtract DNA Solution (Lucigen) and amplicons were generated using 15 cycles of PCR to introduce Illumina sequencing primer binding sites and 0-8 staggered bases to ensure library diversity. After reaction clean-up using ExoSAP-IT kit (Thermo Fisher, 78201), an additional 15 cycles of PCR was used to introduce unique dual indices and Illumina P5 and P7 adaptors. Libraries were pooled and purified by SPRIselect magnetic beads before paired-end sequencing using an Illumina MiSeq at the Arc Institute Multi-Omics Technology Center. Sequencing primer binding sites, unique dual indices (from Illumina TruSeq kits), P5 and P7 adaptor sequences are from Illumina Adaptor Sequences Document #1000000002694 v16. Bioinformatic analysis of indel frequencies and simulation of indel impacts on gene expression, accounting for DNA copy number of the target region in the K562 genome^65^, are detailed in Supplementary Information. Primer sequences are in Supplementary Table 2.

### Nanopore long-read sequencing analysis of deletion frequencies

Genomic DNA was harvested from 20 million cells using the Qiagen Genomic Tips Kit (10243). As detailed in Supplementary Information, we used a custom protocol adapted from the Nanopore Cas9 Sequencing Kit user’s manual (SQK-CS9109, though this kit was not actually used) to enrich for genomic DNA surrounding crRNA target sites for Nanopore sequencing using Kit 14 chemistry. Cas9 guide spacer sequences are in Supplementary Table 1.

fastq files generated by MinKNOW version 23.07.15 (Oxford Nanopore Technologies) were aligned to the ~20-kb regions (defined by the outermost Cas9 sgRNA protospacer sites flanking each targeted locus) surrounding each crRNA target site in MinKNOW to generate bam files. Bam files for each sample were merged using samtool merge (samtools v1.6 (ref. ^120^)). Merged bam files were filtered for alignments that overlap the start and end coordinates of the protospacer region of the Cas12a crRNA using bamtools filter -region (bamtools v2.5.1 (ref. ^121^)). Filtered bam files were loaded into the Integrative Genomics Viewer 2.17.0 (ref. ^122^) for visualization of individual read alignments. pysamstats –fasta –type variation (pysamstats v1.1.2) was used to extract per base total read coverage and deletion counts. The fraction of aligned reads harboring a deletion at each base was plotted using custom scripts in R.

### 3’ RNA-seq

Approximately 200,000 to 1 million cells were harvested, resuspended in 300 µl RNA Lysis Buffer (Zymo, R1060), and stored at −70°C until further processing for RNA isolation using the Quick-RNA Miniprep Kit (Zymo, R1055). 3′ RNA-seq was batch processed together with samples unrelated to this study using a QuantSeq-Pool Sample-Barcoded 3′ mRNA-Seq Library Prep Kit for Illumina (Lexogen cat#139) in accordance with the manufacturer’s instructions. 10 ng of each purified input RNA was used for first-strand cDNA synthesis with an oligo(dT) primer containing a sample barcode and a unique molecular identifier. Subsequently, barcoded samples were pooled and used for second strand synthesis and library amplification. Amplified libraries were sequenced on an Illumina HiSeq4000 with 100-bp paired-end reads. The QuantSeq-Pool data was demultiplexed and preprocessed using an implementation of pipeline originally provided by Lexogen (https://github.com/Lexogen-Tools/quantseqpool_analysis). The final outputs of this step are gene level counts for all samples (including samples from multiple projects multiplexed together). Downstream analyses were performed using DESeq2 (ref. ^123^) for differential expression analysis, crisprVerse^124^ for off-target analysis, and custom R scripts for plotting as detailed in Supplementary Information.

### RT-qPCR

For the CRISPRi experiments targeting the *HBG1*/*HBG2* TSSs or HS2 enhancer, K562 cells engineered (by lentiviral transduction at MOI ~ 5) for constitutive expression of multiAsCas12a-KRAB were transduced with crRNAs and sorted, followed by resuspension of ~200,000 to 1 million cells in 300 µl RNA Lysis Buffer from the Quick-RNA Miniprep Kit (Zymo, R1055) and stored in −70°C. RNA isolation was performed following the kit’s protocols, including on-column DNase I digestion. 500 ng RNA was used as input for cDNA synthesis primed by random hexamers using the RevertAid RT Reverse Transcription Kit (Thermo Fisher, K1691), as per manufacturer’s instructions. cDNA was diluted 1:4 with water and 2 µl used as template for qPCR using 250 nM primers using the SsoFast EvaGreen Supermix (BioRad, 1725200) on an Applied Biosystems ViiA 7 Real Time PCR System. Data was analyzed using the ddCT method, normalized to GAPDH and no crRNA sample as reference. qPCR primer sequences are in Supplementary Table 2.

### Transient transfection experiments

For co-transfection experiments, the day before transfection, 100,000 HEK293T cells were seeded into wells of a 24-well plate. The following day, we transiently transfected 0.6 µg of each protein construct and 0.3 µg gRNA construct per well (in duplicate) in Mirus TransIT-LT1 (MIR 2304) transfection reagent according to manufacturer’s instructions. Mixtures were incubated at room temperature for 30 min and then added in dropwise fashion into each well. 24 h after transfection, cells were replenished with fresh media. 48 h after transfection, BFP and GFP-positive cells (indicative of successful delivery of protein and crRNA constructs) were sorted on a BD FACSAria Fusion and carried out for subsequent flow cytometry experiments.

### Western blotting

Approximately 400,000 cells per sample were washed with 1 ml cold PBS and resuspended in 400 µl Pierce RIPA Buffer supplemented with Halt Protease and Phosphatase inhibitor cocktail (Thermo Fisher, 1861281) on ice. Samples were rotated for 15 min at 4°C, followed by centrifugation at 20,000 *g* for 15 min to pellet cell debris. The supernatant was collected and mixed with 4x Bolt LSD Sample Buffer (Thermo Fisher, B0007) supplemented with 50 mM DTT, followed by heating for 10 min at 70°C. Samples were electrophoresed on Bolt 4%–12% Bis-Tris Plus Gels (Thermo Fisher), and transferred using the BioRad TurboTransfer system onto Trans-Blot Turbo Mini 0.2 µm Nitrocellulose Transfer Packs (1704158). Membranes were blocked with 6% BSA in TBST (Tris-buffered saline, 0.1% Tween 20) at room temperature for ~1 h, followed by incubation at 4°C overnight with antibodies against anti-HA-tag rabbit antibody (Cell Signaling Technology, 3724 S) at 1:1,000 dilution and anti-GAPDH rabbit antibody (Cell Signaling Technology, 2118) at 1:3,000 dilution in 6% BSA in TBST. Membranes were washed with TBST at room temperature three times for 5 min. each, followed by incubation with IRDye secondary antibody for 1 h at room temperature, washed three times with TBST 5 min for each and two times with PBS. Blots were imaged using Odyssey CLx (LI-COR).

### Reporting summary

Further information on research design is available in the Nature Portfolio Reporting Summary linked to this article.

## Online content

Any methods, additional references, Nature Portfolio reporting summaries, source data, extended data, supplementary information, acknowledgements, peer review information; details of author contributions and competing interests; and statements of data and code availability are available at 10.1038/s41587-024-02224-0.

## Supplementary information

Supplementary Information Supplementary Figures 1–22 and Methods.

Reporting Summary

Supplementary Data 1 Statistical source data for Supplementary Figures.

Supplementary Table 1 Sequences of guides and genomic regions.

Supplementary Table 2 Primers and PCR amplicon sequences.

Supplementary Data 2 Annotated sequences for plasmids and Illumina sequencing library prep amplicons.

Supplementary Table 3 Processed screen data.

## Source data

Source Data Fig. 1 Statistical source data.

Source Data Fig. 2 Statistical source data.

Source Data Fig. 3 Statistical source data.

Source Data Fig. 4 Statistical source data.

Source Data Fig. 5 Statistical source data.

Source Data Fig. 6 Statistical source data.

## Data Availability

Plasmids are available on Addgene under accession numbers 217330–217345, and sequence maps are provided in Supplementary Data 2. Raw fastq files and processed data tables are available at the Gene Expression Omnibus accession GSE260832 (ref. ^125^) and in Supplementary Table 3. Source data are provided with this paper.

## Code availability

Code for read counting for single-plex screens is available at https://noamteyssier.github.io/sgcount/. Code for read counting for 6-plex screens is available at https://github.com/noamteyssier/casmap. R scripts for downstream screen analyses are deposited on Zenodo (10.5281/zenodo.10784378)^126^. Custom R functions used for crRNA design, flow cytometry, and screen analyses are available as an R package at https://github.com/chris-hsiung/bears01.
