# Supplementary material for: Engineered CRISPR-Cas12a for higher-order combinatorial chromatin perturbations
Source: Nat Biotechnol. 2024 May 17;43(3):369–83. doi: 10.1038/s41587-024-02224-0 (PMC11919711; doi:10.1038/s41587-024-02224-0)
Supplement: Supplementary file 1 — Supplementary Figures 1–22 and Methods. [file 41587_2024_2224_MOESM1_ESM.pdf]

nature biotechnology

Article https://doi.org/10.1038/s41587-024-02224-0

Engineered CRISPR-Cas12a for higher-order combinatorial chromatin perturbations

In the format provided by the authors and unedited

Table of Contents for Supplementary Figures

S1 Example of flow cytometry gating strategy for CRISPRi experiments. 2
S2 Additional replicate testing dAsCas12a-3xKRAB CRISPRi activity. 3
S3 Western blot of fusion proteins. 4
S4 CRISPRi activity of multiCas12a-KRAB, denAsCas12a-KRAB, and dAsCas12a-3xKRAB in C4-2B cells. 5
S5 dAsCas12a-3xKRAB CRISPRi by transient transfection in HEK 293T cells. 6
S6 Comparisons of dAsCas12a variant fusion CRISPRi constructs using up to 3-plex crRNA constructs. . 7
S7 Additional replicates testing effect of dose on denAsCas12a-KRAB CRISPRi activity 8
S8 Testing CRISPRi activity of lentivirally delivered truncated crRNAs 9
S9 CD81 knockdown by denAsCas12a-KRAB vs. multiAsCas12a-KRAB at different protein and crRNA MOIs 10
S10 CD55 knockdown by denAsCas12a-KRAB vs. multiAsCas12a-KRAB at different protein MOIs . . . 11
S11 RNA-seq analysis of crRNA specificity. 12
S12 CRISPRi knockdown of CD55 and B2M using up to 6-plex crRNA arrays. 13
S13 Monitoring P2A-BFP reporter as proxy of fusion protein expression level. 14
S14 Indel quantification and gene expression knockdown simulation for single-targeting. 15
S15 Indel quantification and gene expression knockdown simulation for dual-targeting of the KIT TSS region. 16
S16 Long-read Nanopore sequencing quantification of deletions surrounding single and dual crRNA target sites. 17
S17 Double and triple gene knockdown by CRISPRi using higher-order crRNA arrays. 18
S18 Summaries of Library 1 and Library 2 screens. 19
S19 Screen replicate concordance for Library 1 and Library 2. 20
S20 Cell fitness score distributions of top multiAsCas12a-KRAB vs. dCas9-KRAB TSS-targeting guides and negative control crRNAs from pooled screens. 21
S21 Relationship among CRISPick predictions, PAM sequence, and empirical CRISPRi activity. 22
S22 Difference in cell fitness scores for 6-plex vs. 1-plex crRNA constructs from pooled screens. 23

Table of Contents for Supplementary Methods

1 Simulations of indel impacts on gene expression 24
2 Description of Library 2 screen read mapping algorithm for 6-plex crRNA constructs 24
3 Golden Gate assembly and bacterial transformation for pooled crRNA library cloning 24
4 Bioinformatics processing of indel quantificaition by short-read sequencing of PCR amplicons 25
5 Analysis of 3' RNA-seq data 25
6 Nanopore long-read sequencing analysis of deletion frequencies 26

Supplementary Figures

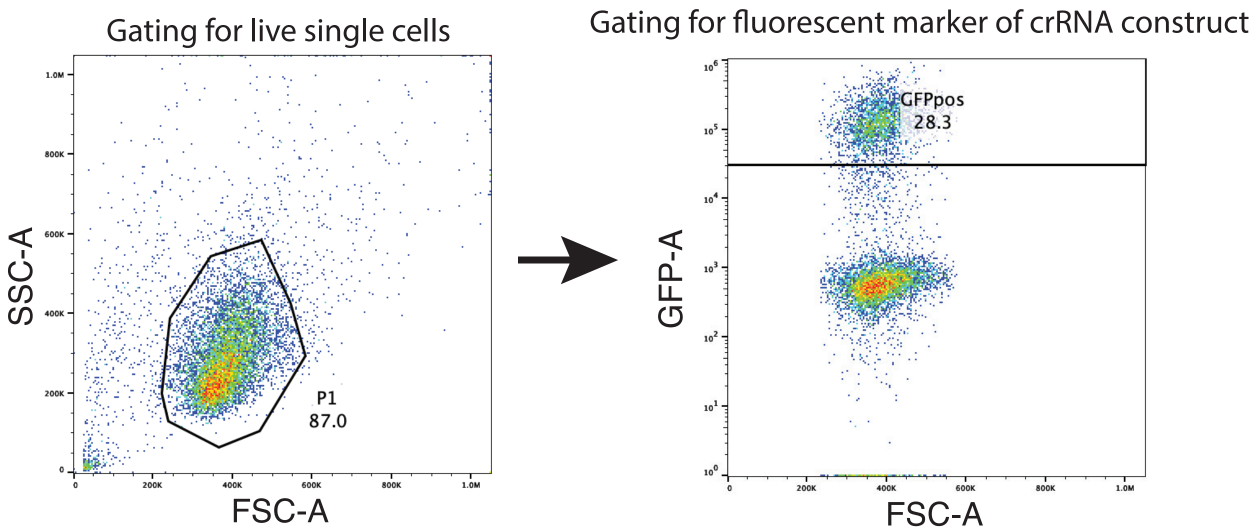

Figure S1 – Example of flow cytometry gating strategy for CRISPRi experiments. Example of the general gating strategy for CRISPRi experiments using flow cytometry readouts, shown for K562 cells as an example. Single live cells are gated by FSC vs. SSC, followed by gating for the fluorescent marker on the crRNA construct (typically GFP), which are subsequently analyzed for target gene expression in the respective fluorescence channels.

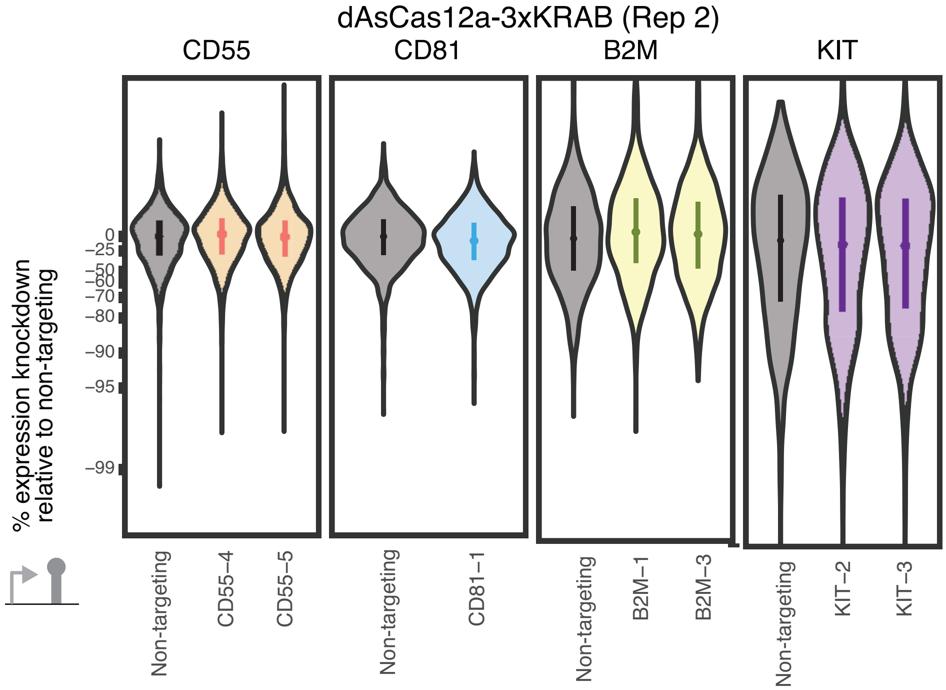

Figure S2 – Additional replicate testing dAsCas12a-3xKRAB CRISPRi activity.
Additional biological replicate for Fig. 1B. K562 cells constitutively expressing dAsCas12a-3xKRAB were lentivirally transduced with single crRNAs targeting CD55, CD81, B2M, KIT, or a non-targeting crRNA, and assayed by flow cytometry 6 days after crRNA transduction. Shown are single-cell distributions (N>200 cells for each condition) of gene expression with overlaid with median and interquartile ranges.

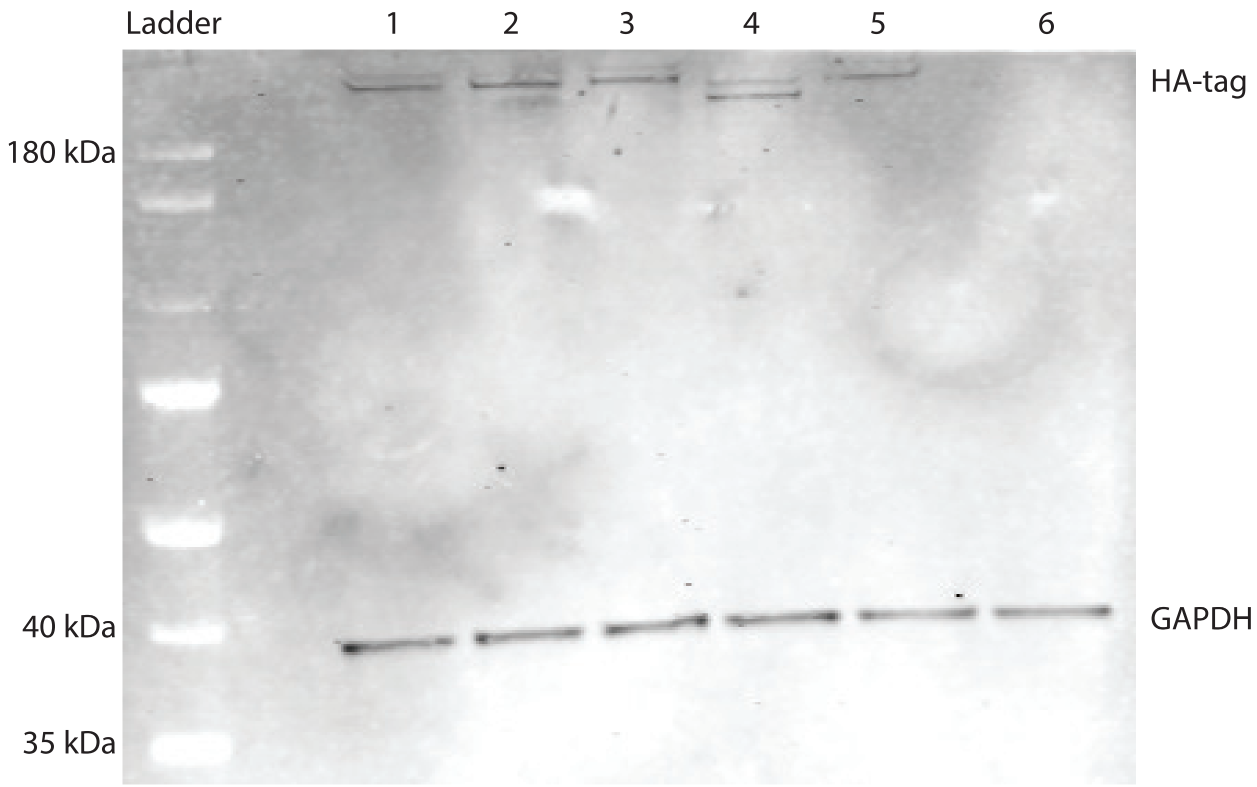

Well	Protein construct	Plasmid ID
1	multiAsCas12a-HA-XTEN80-KRAB-P2A-BFP	pCH61
2	denAsCas12a-HA-XTEN80-KRAB-P2A-BFP	pCH62
3	enAsCas12a-HA-XTEN80-KRAB-P2A-BFP	pNS49
4	multiAsCas12a-HA-P2A-BFP	pNS35
5	dAsCas12a-3xKRAB-3xHA-P2A-BFP	pCH68
6	No construct	No construct

Figure S3 – Western blot of fusion proteins. Western blot of whole-cell lysates prepared from K562 cells piggyBac engineered to constitutively express each of the fusion proteins in the panel and previously sorted for similar levels of BFP expression. anti-HA tag was used for detection of the fusion protein and anti-GAPDH for detection of GAPDH as loading control. Shown is a representative blot from two gel electrophoresis experiments.

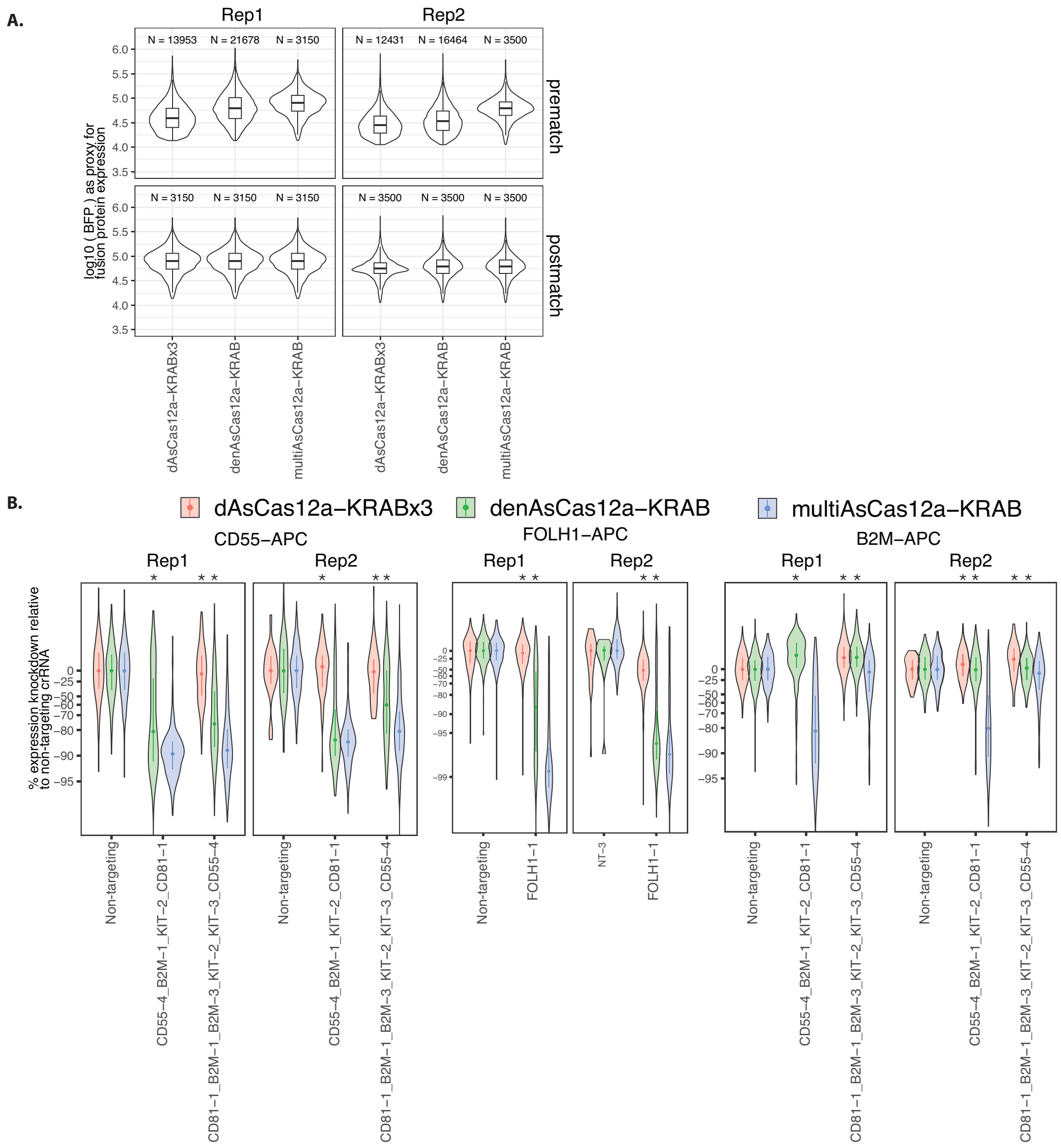

Figure S4 – CRISPRi activity of multiCas12a-KRAB, denAsCas12a-KRAB, and dAsCas12a-3xKRAB in C4-2B cells.
A) C4-2B cells piggyBac-engineered to constitutively express each of the fusion protein constructs are lentivirally transduced with the indicated crRNA constructs. Cells were sorted based on P2A-BFP marker signal. Because some of these cell lines showed slightly different levels of BFP signal as a proxy of fusion protein expression, to account for fusion protein expression we performed propensity score matching to subset for populations of cells for each fusion protein construct with the same distributions in BFP signals after data acquisition for flow cytometry in CRISPRi experiments. The BFP signals before and after matching are shown as violin plots for each of 2 biological replicates, overlaid with boxplots showing median and interquartile range. Numbers of cells before and after propensity score matching are indicated.
B) Target gene expression are measured by cells surface antibody staining and flow cytometry 13-14 days after crRNA transduction. After propensity score matching for BFP levels as described in A, single-cell distributions of expression knockdown relative to non-targeting crRNA are shown for each of 2 biological replicates, with median and interquartile range overlaid using the cells. One-sided Wilcoxon rank-sum test was used to compare multiAsCas12a-KRAB knockdown vs. each of the other fusion protein constructs (N = 11-929 cells per replicate after propensity score matching); asterisks indicate $p<0.01$. CRISPRi knockdown results are indistinguishable when analyzed with and without (not shown) propensity score matching.

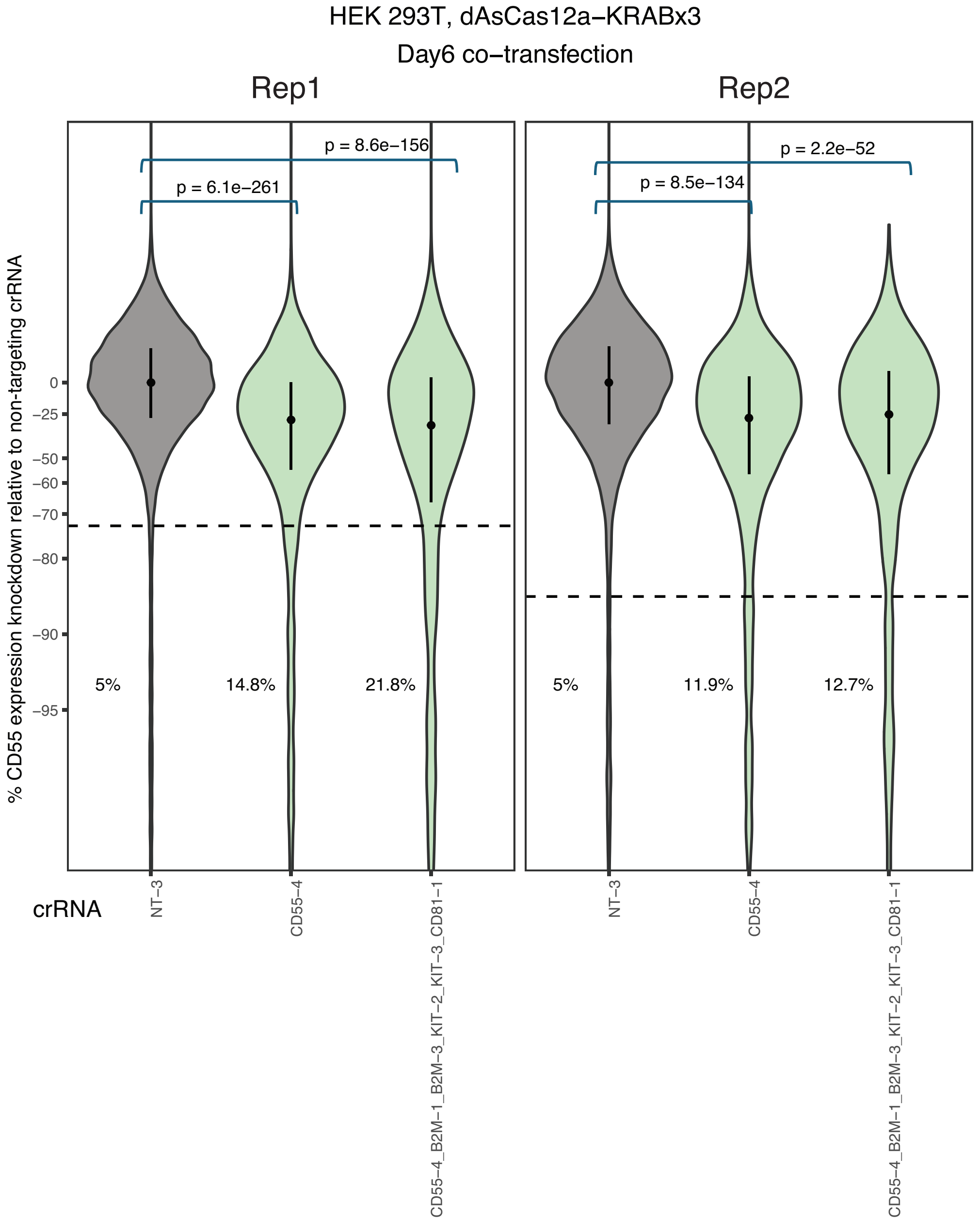

Figure S5 – dAsCas12a-3xKRAB CRISPRi by transient transfection in HEK 293T cells. HEK 293T cells were co-transfected with a plasmid encoding for dAsCas12a-3xKRAB and plasmids encoding for the indicated crRNA constructs targeting CD55. Cells were sorted 2 days after transfection for successful co-transfection based on BFP and GFP markers on the plasmids and CD55 expression was measured by antibody staining on flow cytometry 6 days after transfection. Violin plots of single-cell distributions of CD55 expression knockdown as a percentage of the median of non-targeting control are shown for 2 biological replicates. Median and interquartile range are shown in the plot. The percentage of cells below the 5th percentile of the non-targeting control are also shown. One-sided Wilcoxon rank-sum test was performed to compare the single-cell expression distribution of non-targeting crRNA vs. each of the other crRNA constructs, with p-values indicated (N = 1,213-15,835 cells).

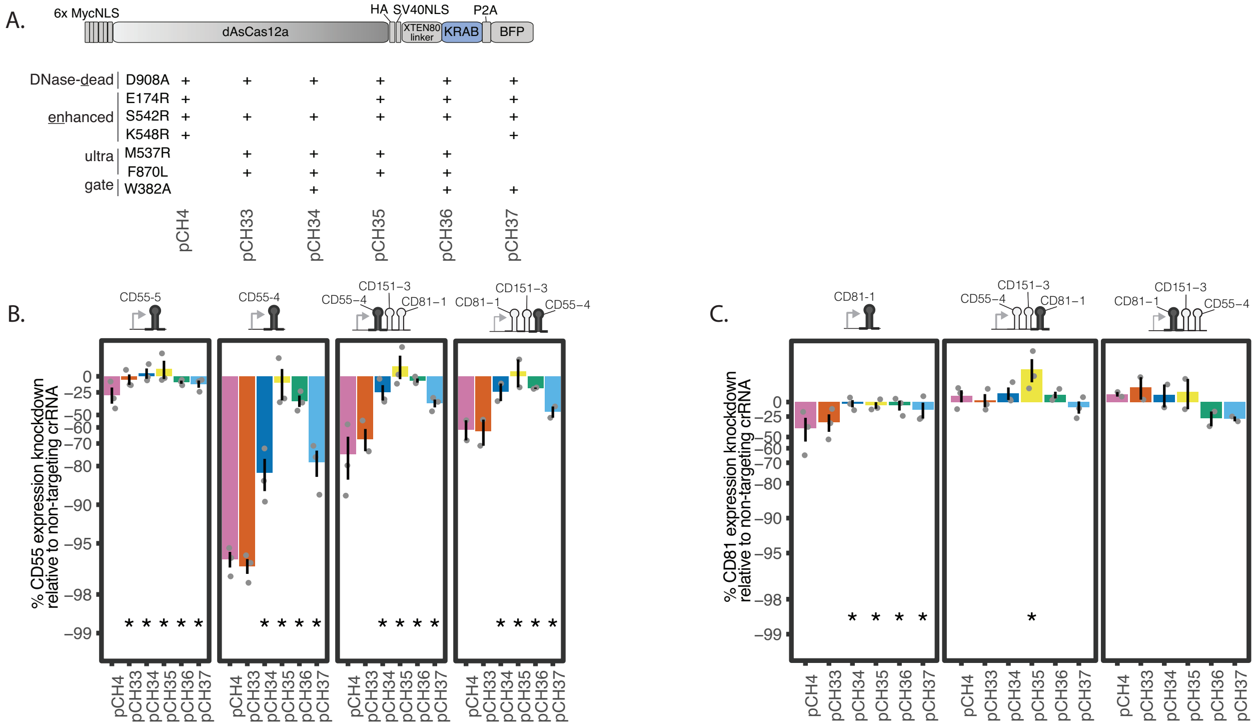

Figure S6 – Comparisons of dAsCas12a variant fusion CRISPRi constructs using up to 3-plex crRNA constructs.

A) The same fusion protein schematic as shown in Fig. 1C, labeled with construct IDs for ease of reference.

B) CD55 expression knockdown measured by flow cytometry using the indicated crRNA constructs and the panel of fusion protein constructs in A. Bars indicate averages of the median single-cell expression knockdown relative to non-targeting crRNA for 3 biological replicates (including the replicate for crCD55-4 shown in Fig. 1C) for all comparisons, except the comparison for crCD81-1_crCD151-3_crCD55-4 contains 2 biological replicates. Error bars indicate SEM. One-sided Wilcoxon rank-sum test of single-cell distributions of gene expression comparing denAsCas12a-KRAB (pCH4) to each of the other fusion constructs in the panel was performed (N>200 cells for each biological replicate). Asterisks indicate p<0.01 for all replicate-level comparisons for a given construct.

C) Analogous to B, but for CD81 knockdown. Summaries shown for 3 biological replicates (including the replicate for crCD81-1 shown in Fig. 1C) for all comparisons, except the comparison for crCD81-1_crCD151-3_crCD55-4 contains 2 replicates.

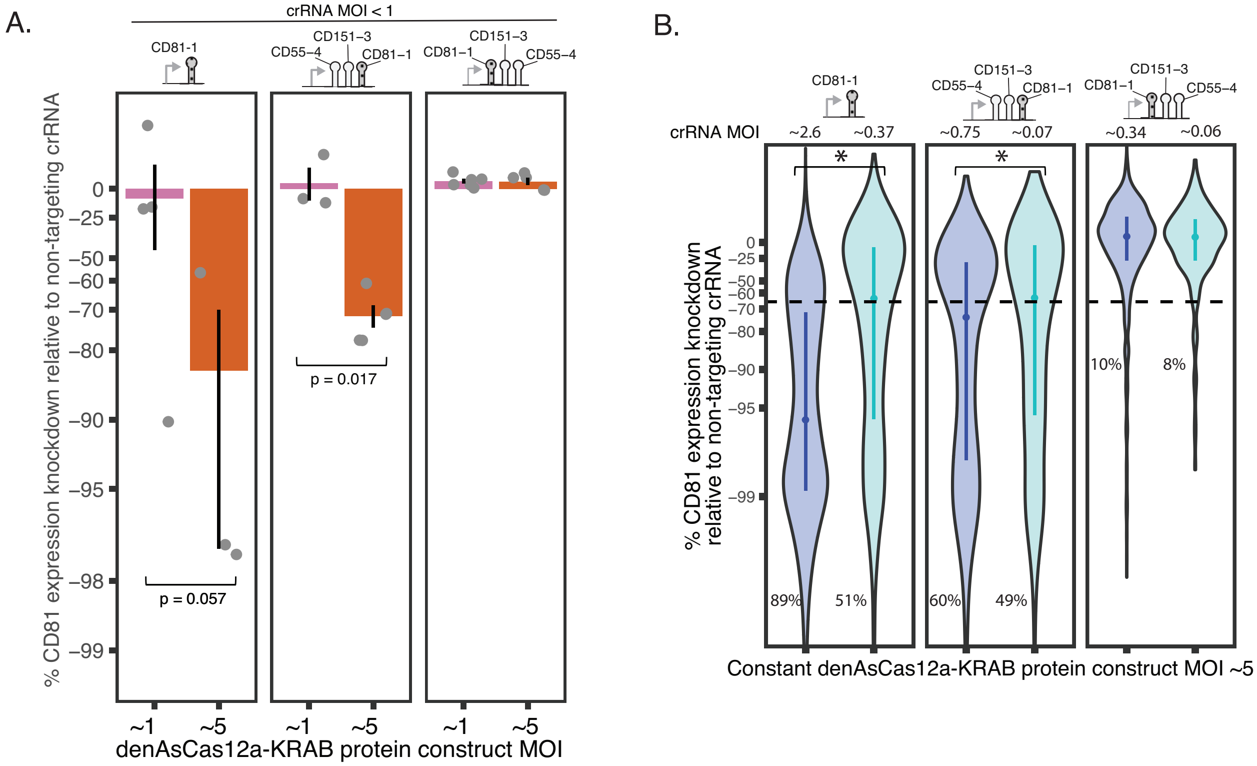

Figure S7 – Additional replicates testing effect of dose on denAsCas12a-KRAB CRISPRi activity

A) Summary of all replicates for experiment shown in Fig. 1D. Analysis of CD81 knockdown in cells lentivirally transduced with denAsCas12a-KRAB protein construct at MOI ∼1 vs. MOI ∼5, while maintaining constant crRNA MOI (<0.74) for each crRNA construct. CD81 expression was assayed by flow cytometry 6 days after crRNA transduction. Shown are averages of median expression knockdown for each crRNA construct (from left to right: N = 4, 3, 3, 5, 6, and 5 biological replicates per condition shown as individual dots for each replicate, including the replicate shown in Fig. 1D). Error bars denote SEM. One-sided Wilcoxon rank-sum test was performed on the medians of single-cell expression and p-values indicated where relevant.

B) Additional biological replicate for Fig. 1E. Analysis of CD81 knockdown in cells lentivirally transduced with denAsCas12a-KRAB protein construct at MOI ∼5, while crRNA MOI is changed from high to low as indicated. CD81 expression was assayed by flow cytometry 10 days after crRNA transduction. Shown are single-cell distributions of CD81 knockdown for the replicate. One-sided Wilcoxon rank-sum test was performed on single-cell distributions (N>200 cells) for the replicate; asterisks indicates $p<0.01$.

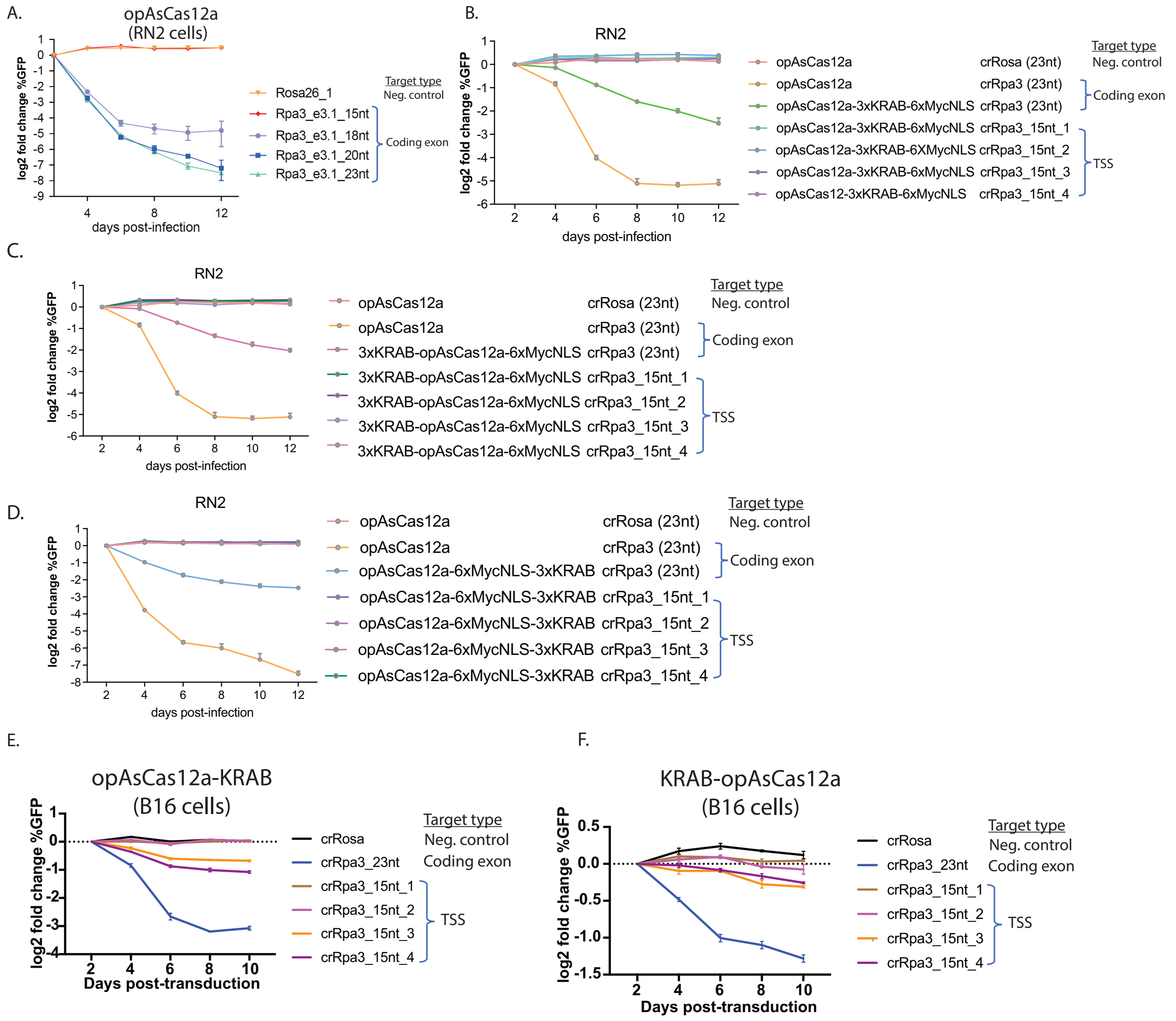

Figure S8 – Testing CRISPRi activity of lentivirally delivered truncated crRNAs. In all panels, the indicated cell line (RN2 or B16) was engineered for constitutive expression of the indicated fusion protein constructs by lentiviral transduction, followed by lentiviral transduction (at MOI between 0.3-0.4) of the indicated single-plex crRNA constructs containing spacers of the indicated lengths targeting Rpa3, an essential gene. The spacers target either the gene's coding exon, the TSS region, or the Rosa locus (negative control) as indicated in the legends. Cell fitness defect is used as a proxy of Rpa3 gene expression knockdown due to either DNA cutting or CRISPRi activity. Cell fitness is measured in a competition assay by quantifying log2 fold change in percent of cells expressing the GFP marker on the crRNA expression constructs (relative to day 2). Error bars indicate SEM for N = 3 biological replicates for all panels.

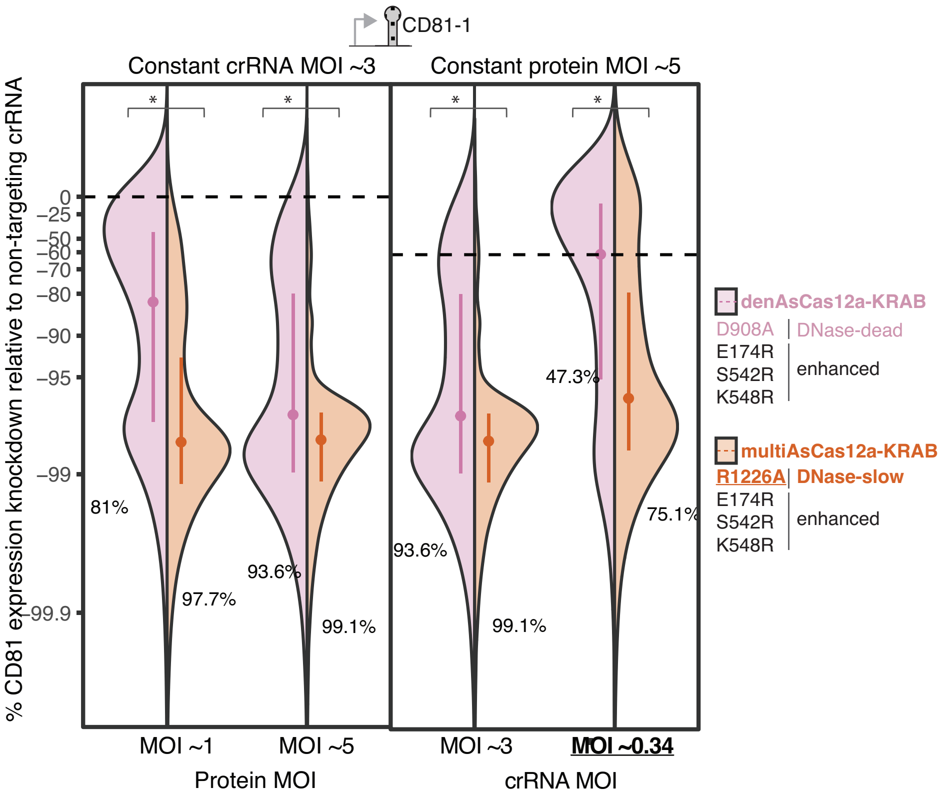

Figure S9 – CD81 knockdown by denAsCas12a-KRAB vs. multiAsCas12a-KRAB at different protein and crRNA MOIs. Second biological replicate for Fig. 2B. Comparison of denCas12a-KRAB (D908A/E174R/S542R/K548R) vs. multiAsCas12a-KRAB (R1226A/E174R/S542R/K548R) in CRISPRi knockdown of CD81 using crCD81-1. CD81 expression assayed by flow cytometry 10 days after crRNA transduction. Left panel: Holding crRNA MOI constant at ∼3 while testing protein MOI ∼1 vs. ∼5. Right panel: Holding protein MOI constant at ∼5 while testing crRNA MOI at ∼3 vs. ∼0.5. Asterisks indicate p<0.01 for one-sided Wilcoxon rank-sum test of single-cell distributions (N>200 cells per replicate). Percentage of cells below the 5th percentile of non-targeting crRNA control (dashed line) is shown for each condition.

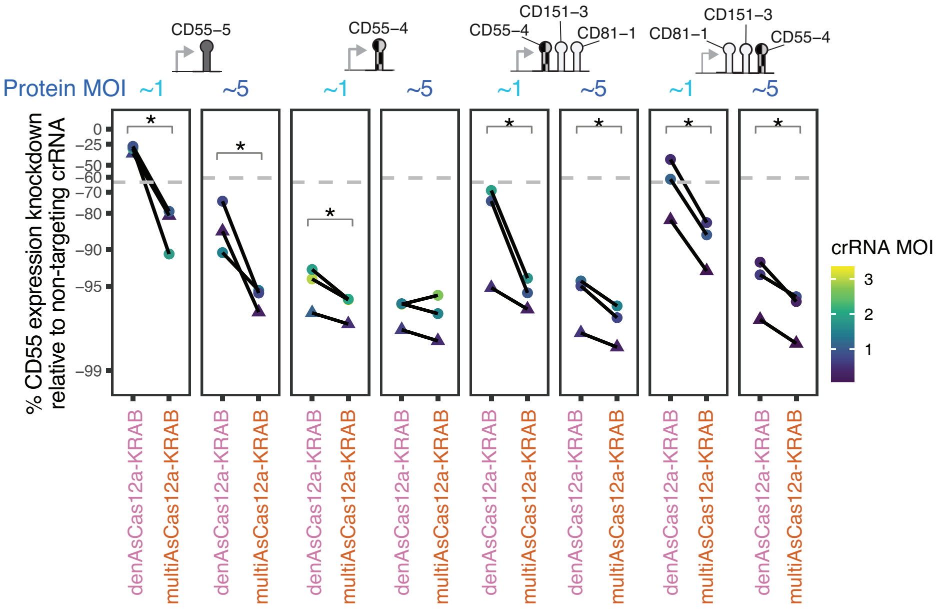

Figure S10 – CD55 knockdown by denAsCas12a-KRAB vs. multiAsCas12a-KRAB at different protein MOIs. Comparison of CD55 knockdown by lentivirally delivered denAsCas12a-KRAB vs. multiAsCas12a-KRAB at protein MOI ∼1 vs. ∼5 across a panel of single and 3-plex crRNA constructs, while holding constant crRNA MOI for each paired fusion protein comparison for each crRNA construct. Dashed gray line indicates 5th percentile of non-targeting crRNA control. crRNA MOI indicated by color scale. Lines connect paired experiments performed on the same day. One-sided Wilcoxon rank-sum tests were performed on single-cell distributions for each replicate, and asterisk denotes $p<0.01$ for all paired replicates within each condition ($N>200$ cells per replicate). Dots indicate flow cytometry measurement 10 days after crRNA transduction; triangles indicate flow cytometry measurement 16 days after crRNA transduction.

A.

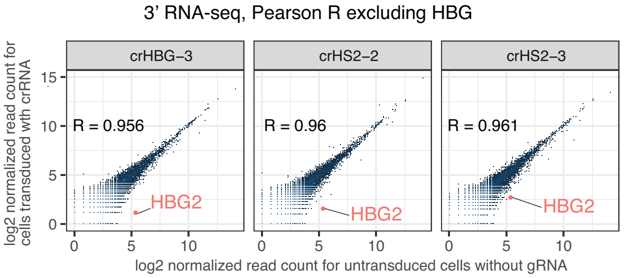

B.

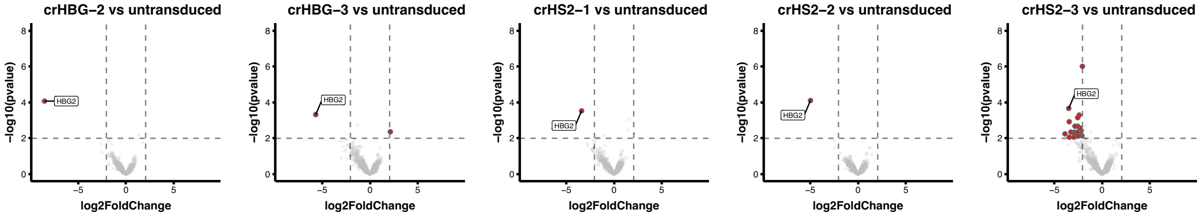

C.

crHBG-3 differentially expressed genes

	log2FoldChange	pvalue
COL6A3	-2.111480489	0.032227

crHS2-3 differentially expressed genes

	log2FoldChange	pvalue
COL6A3	-2.111480489	0.032227
ITGA3	-1.754297196	0.039295
PHLDA1	-1.627213967	0.143479
MALAT1	-1.321521059	0.001853
UCHL1	-2.374917208	0.011372
HBG2	-5.687020138	0.00048
TM4SF1	-1.471229856	0.105152
CD44	-1.689959013	0.025554
IGFBP7	-1.39428827	0.077142
CTSA	-1.778803563	0.033146
ALDH1A1	-1.336719338	0.071234
KRT81	-1.53327734	0.072989
THBS1	-1.179123518	0.252654
APP	-1.867798594	0.019031
CCND1	-1.879166671	0.066226
ANXA1	-1.720793771	0.046504
ALDH3A1	-1.879322885	0.03484
TGFBI	-1.58211392	0.030056
NEAT1	-1.670821249	0.013372
HMOX1	-1.762705604	0.067726
PLS3	-2.890643152	0.014401
SERPINE1	-1.635079753	0.067127

All crisprVerse off-target predictions

chr	TSS coordinate	Gene name
chr2	120211053	MTATP6P26
	120211726	MTCO3P43
	120212934	MTND3P10
	120213343	MTND4LP14
	120213630	MTND4P26
chr11	5249857	HBG1
	5254783	AC104389.6
chr12	7838913	AC006517.6
	7954215	AC006511.7
chr17	57531768	AC007431.3

Figure S11 – RNA-seq analysis of crRNA specificity.

A) K562 cells lentivirally engineered (MOI ∼5) to constitutively express multiAsCas12a-KRAB were either transduced with the indicated crRNA’s at MOI <0.3, followed by sorting for crRNA-transduced cells based on GFP marker, or received no crRNAs. RNA was isolated from the sorted cells 32 days of culture after crRNA transduction and subjected to 3’ RNA-seq. Scatter plot of normalized mRNA expression levels for crRNA transduced (1 biological replicate each) vs. cells without crRNA (2 biological replicates), and Pearson correlation coefficient calculated for the transcriptome, excluding HBG2. RT-qPCR quantifications are shown in Fig. 5A.

B) Differential expression analysis using DE-seq2 was performed for the no crRNA control vs. each crRNA. Volcano plots of p-values (without multiple testing adjustment) from two-sided Wald test vs. log2FoldChange from genes that fall beyond p-value and log2FoldChange cutoffs (dashed lines) are highlighted.

C) Lists of differentially expressed genes (other than HBG2) from the analysis in B for the crHBG-3 and crHS2-3 transduced cells are shown. For comparison, a list of all off-target predictions generated by crisprVerse are shown for all crRNAs in the panel in A and B. DE-seq2 outputs of p-values (without multiple testing adjustment) using two-sided Wald test are shown.

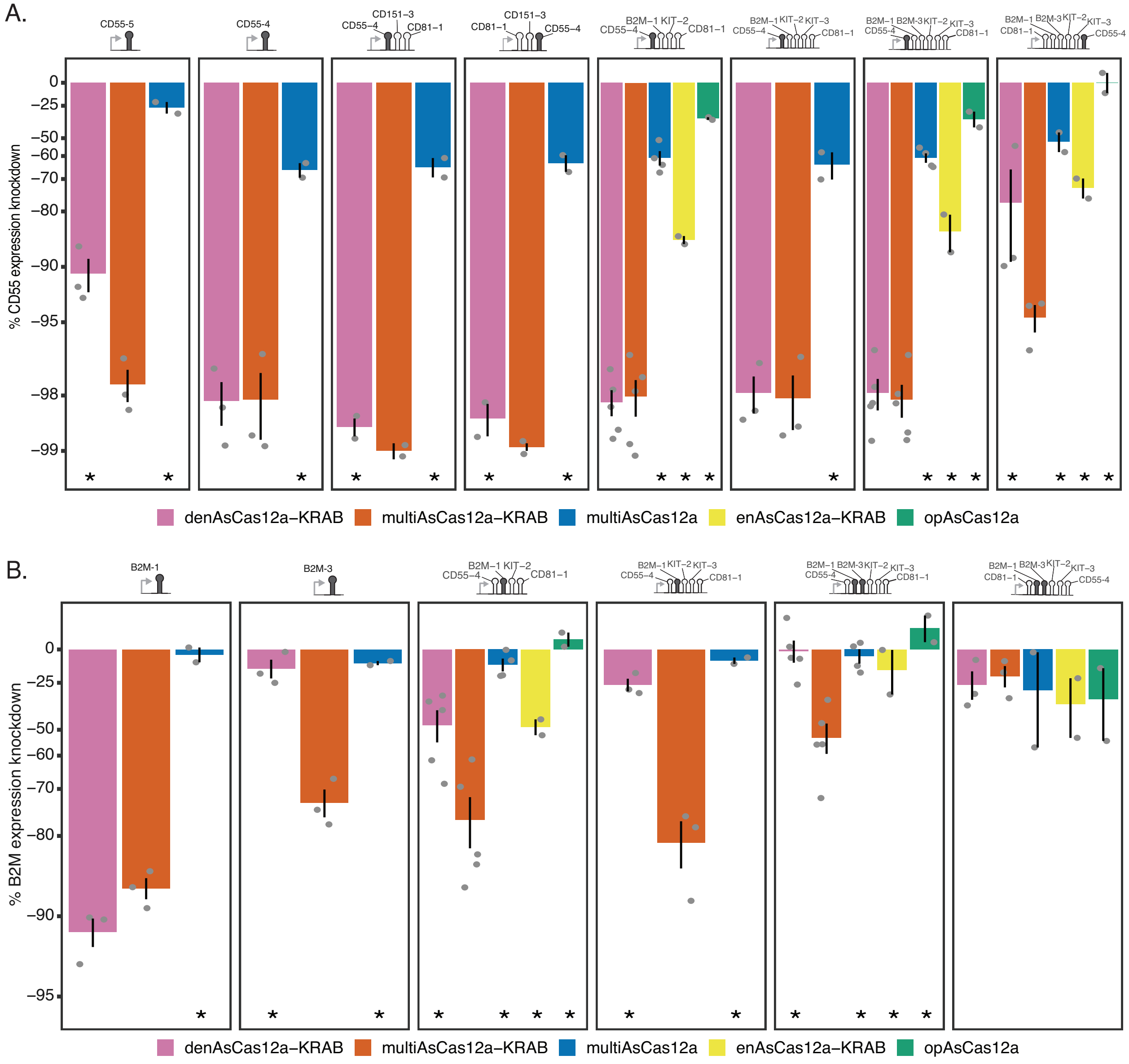

Figure S12 – CRISPRi knockdown of CD55 and B2M using up to 6-plex crRNA arrays.
A) Flow cytometry analysis of CD55 expression knockdown by antibody staining 6 days after transduction of the indicated lentiviral crRNA constructs in K562 cells engineered to constitutively express the specified fusion protein construct. Shown are averages of median single-cell expression knockdown from 2-5 biological replicates for each crRNA construct, with replicates shown as individual data points and error bars indicating SEM. One-sided Wilcoxon rank-sum test was used to compare the single-cell expression distribution for multiAsCas12a-KRAB vs. each of the other fusion protein constructs within each biological replicate. Asterisks indicate $p<0.01$ for all replicate-level comparisons within a given condition ($N>200$ cells for each biological replicate).
B) Analogous to A, but for B2M knockdown.

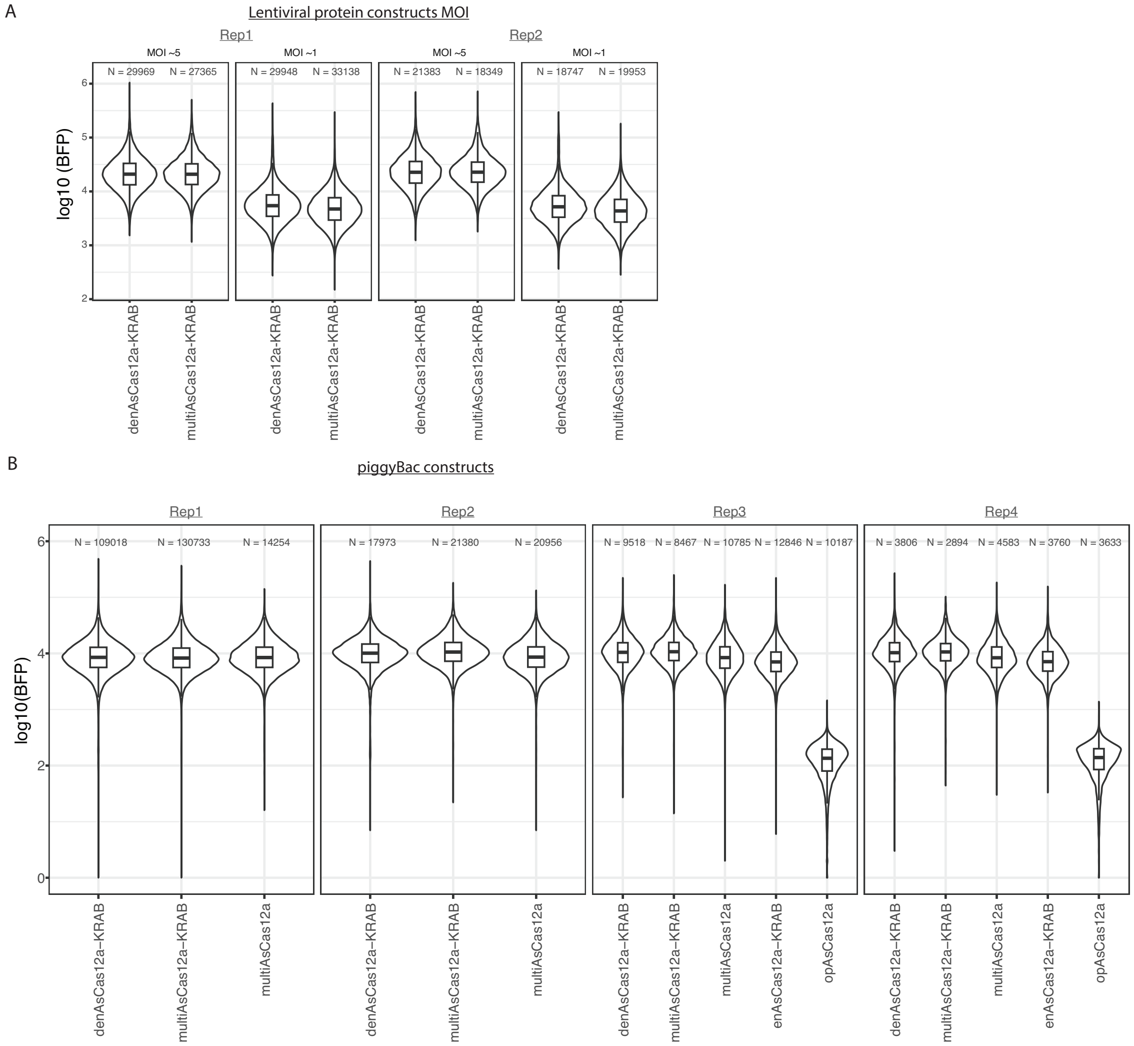

Figure S13 – **Monitoring P2A-BFP reporter as proxy of fusion protein expression level.**

A) K562 cells lentivirally engineered (at MOI ∼1 or MOI ∼5) to constitutively express the indicated fusion protein constructs were monitored for P2A-BFP expression levels by flow cytometry. Shown are 2 representative biological replicates from routine monitoring.

B) Same as A for K562 cells piggyBac-engineered to constitutively express the indicated fusion protein constructs, except opAsCas12a was lentivirally transduced. opAsCas12a does not contain BFP reporter and is shown as fluorescence negative control. Shown are 4 representative biological replicates from routine monitoring.

A-B) Boxplots denote medians and interquartile ranges and cell numbers (N) are shown.

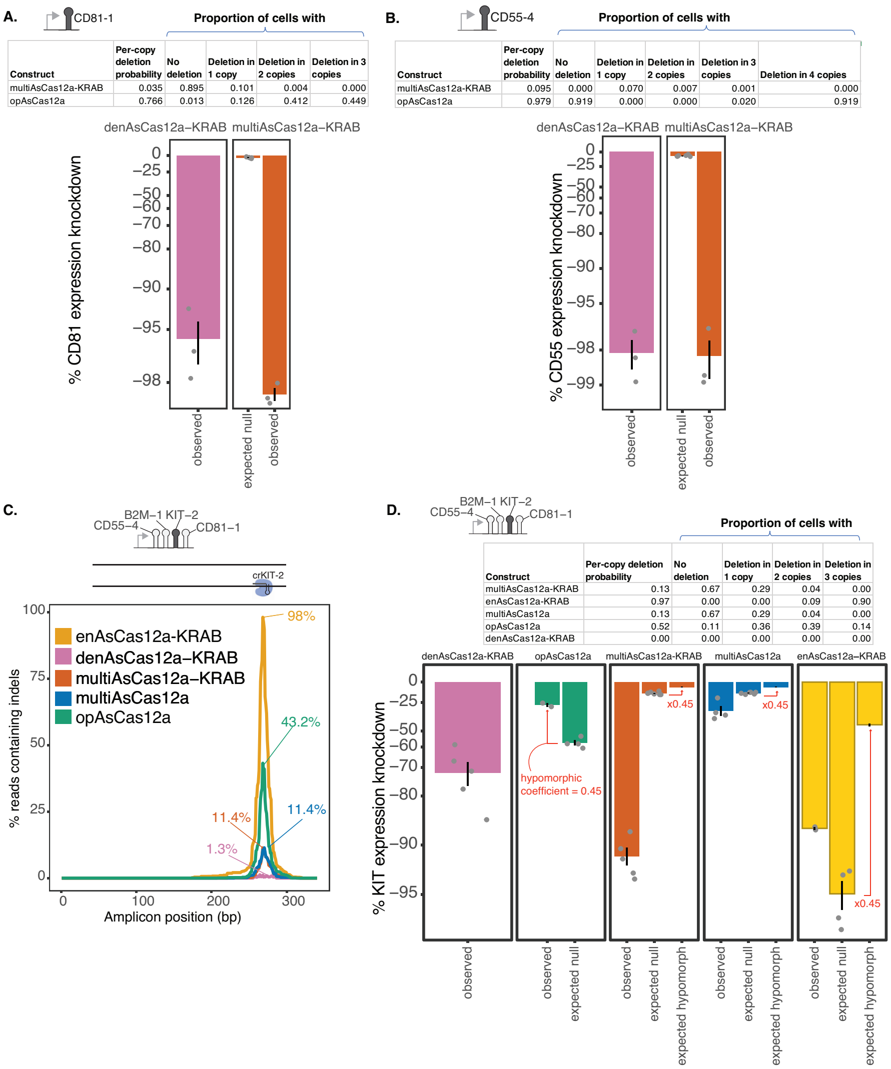

A. CD81-1

Construct	Per-copy deletion probability	No deletion	Deletion in 1 copy	Deletion in 2 copies	Deletion in 3 copies
multiAsCas12a-KRAB	0.035	0.895	0.101	0.004	0.000
opAsCas12a	0.766	0.013	0.126	0.412	0.449

B. CD55-4

Construct	Per-copy deletion probability	No deletion	Deletion in 1 copy	Deletion in 2 copies	Deletion in 3 copies	Deletion in 4 copies
multiAsCas12a-KRAB	0.095	0.000	0.070	0.007	0.001	0.000
opAsCas12a	0.979	0.919	0.000	0.000	0.020	0.919

D.

Construct	Per-copy deletion probability	No deletion	Deletion in 1 copy	Deletion in 2 copies	Deletion in 3 copies
multiAsCas12a-KRAB	0.13	0.67	0.29	0.04	0.00
enAsCas12a-KRAB	0.97	0.00	0.00	0.09	0.90
multiAsCas12a	0.13	0.67	0.29	0.04	0.00
opAsCas12a	0.52	0.11	0.36	0.39	0.14
denAsCas12a-KRAB	0.00	0.00	0.00	0.00	0.00

Figure S14 – Indel quantification and gene expression knockdown simulation for single-targeting.
A) To obtain an upper estimate of the impact on gene expression arising from indels induced by crCD81-1 at the target site, we added an additional 20% unobserved large deletions (upper estimate based on long-read sequencing in Fig. S16) to the observed indel frequency from short-read sequencing of PCR amplicon (Fig. 2F) to arrive at an estimated per-copy deletion probability. Based on this estimate, the proportions of cells harboring deletions at varying DNA copy numbers are shown assuming independent probability of indel formation across the 3 DNA copies in this region of the K562 genome. Assuming that an indel of any size within the PCR amplicon results in a complete genetic null abolishing CD81 gene expression in cis, we simulate the expected effect of these indels on single-cell distributions of gene expression measured by flow cytometry ("expected null", simulated from 7 biological replicates of non-targeting crRNA CD81 expression distributions), compared to the observed gene expression knockdown by crCD81-1 ("observed", from 3 biological replicates).
B) Analogous to A, but for crCD55-4, which targets a tetraploid region of the K562 genome. "Expected null" was simulated from 7 biological replicates of non-targeting crRNA CD55 expression distributions. "Observed" was measured from 3 biological replicates. **C)** Indel quantification by short-read sequencing of PCR amplicon for a single-site targeting of the KIT TSS region using crKIT-2 encoded within the indicated 4-plex crRNA array.
D) Analogous to A-B, we simulated expected gene expression knockdown by crKIT-2 single-site targeting based on indel frequencies observed in C. Expected knockdown under this genetic null assumption exceeds that observed for opAsCas12a (fully active DNase), demonstrating the genetic null assumption is an overestimate of gene expression effects of indels in this region. To correct for this overestimate, we use the ratio of observed vs. expected null median expression knockdown by opAsCas12a as an estimate of the hypomorphic effect of deletions in this region ("hypomorphic coefficient"). We multiply the expected null median expression knockdown for all other fusion proteins by this hypomorphic coefficient to obtain an "expected hypomorph" median expression knockdown, which we propose as our final estimate of the effects arising from indels. "Expected" simulations are derived from 4-7 biological replicates of non-targeting crRNA KIT expression distributions. "Observed" measurements obtained from 2-5 biological replicates.
A-B, D) Individual data points represent medians of single-cell distributions from individual biological replicates. Error bars denote SEM.

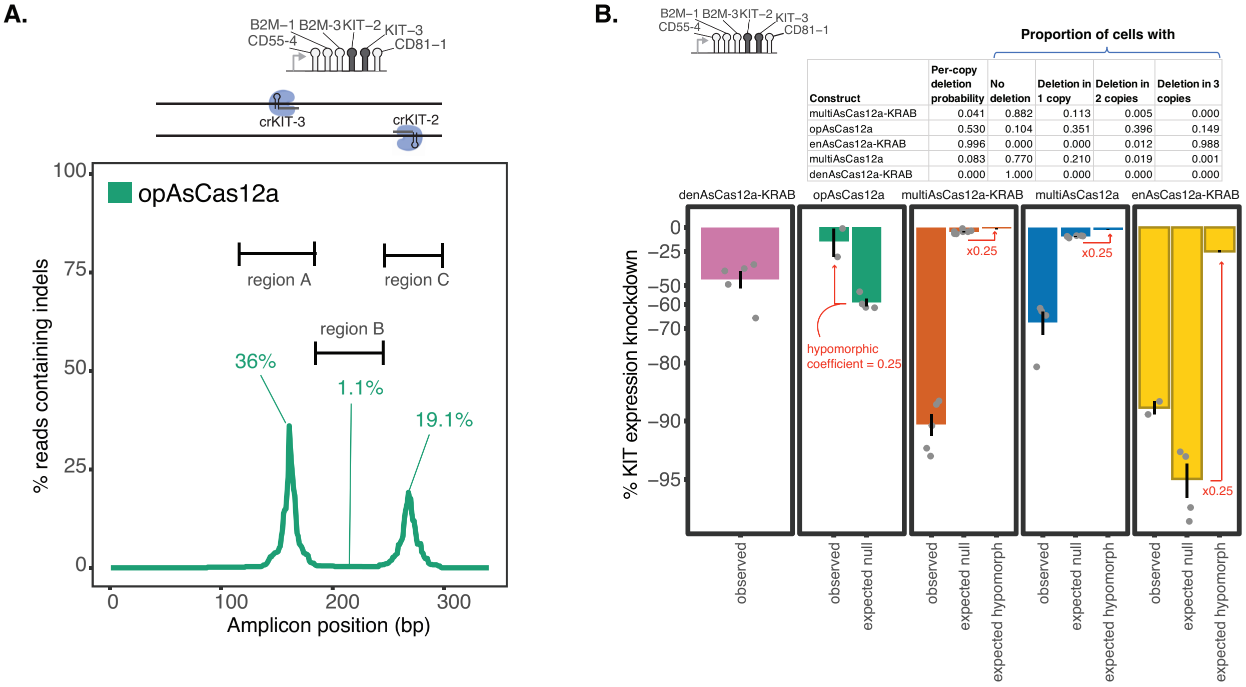

Construct	Per-copy deletion probability	No deletion	Deletion in 1 copy	Deletion in 2 copies	Deletion in 3 copies
multiAsCas12a-KRAB	0.041	0.882	0.113	0.005	0.000
opAsCas12a	0.530	0.104	0.351	0.396	0.149
enAsCas12a-KRAB	0.996	0.000	0.000	0.012	0.988
multiAsCas12a	0.083	0.770	0.210	0.019	0.001
denAsCas12a-KRAB	0.000	1.000	0.000	0.000	0.000

Figure S15 – Indel quantification and gene expression knockdown simulation for dual-targeting of the KIT TSS region. **A)** Related to Fig. 3D. Indel quantification of PCR amplicon near the KIT TSS region in K562 cells lentivirally engineered to constitutively express opAsCas12a 15 days after transduction of the indicated 6-plex crRNA array (sorted for crRNA transduced cells on 2 days after transduction). Note that opAsCas12a is encoded in a different expression backbone using a puromycin selectable marker and thus is not directly matched to other fusion constructs in Fig. 3D in transgenic expression level.

B) We estimated the per-copy deletion probability occuring any where within the PCR amplicon in the KIT locus in Fig. 3D and Fig. S15A based on the on the observed indel allelic frequencies from short-read sequencing, plus an additional 20% unobserved large deletions (upper estimate based on long-read sequencing in Fig. S16). A single composite per-copy deletion probability is assigned to the locus assuming independent probabilities of small indels generated separately at the crKIT-2 and crKIT-3 target sites, plus assuming that large deletions at one crRNA target site precludes additional alterations in DNA sequence at the other target site. The proportions of cells that harbor a specified number of DNA copies containing indels of any size in this locus are calculated assuming indels occur independently across DNA copies within each cell. We simulated the expected distribution of single-cell gene expression levels under the assumption that indels of any size in this locus result in a genetic null abolishing KIT expression in cis ("expected null"). The expected knockdown under this genetic null assumption exceeds that observed for opAsCas12a (fully active DNase), demonstrating the genetic null assumption is an overestimate of gene expression effects of indels in this region. To correct for this overestimate, we use the ratio of observed vs. expected null median expression knockdown by opAsCas12a as an estimate of the hypomorphic effect of deletions in this region ("hypomorphic coefficient"). We multiply the expected null median expression knockdown for all other fusion proteins by this hypomorphic coefficient to obtain an "expected hypormorph" median expression knockdown, which we propose as our final estimate of the effects arising from indels. "Expected" simulations are derived from 4-7 biological replicates of non-targeting crRNA KIT expression distributions. "Observed" measurements obtained from 2-5 biological replicates. Individual data points represent medians of single-cell distributions from individual biological replicates. Error bars denote SEM.

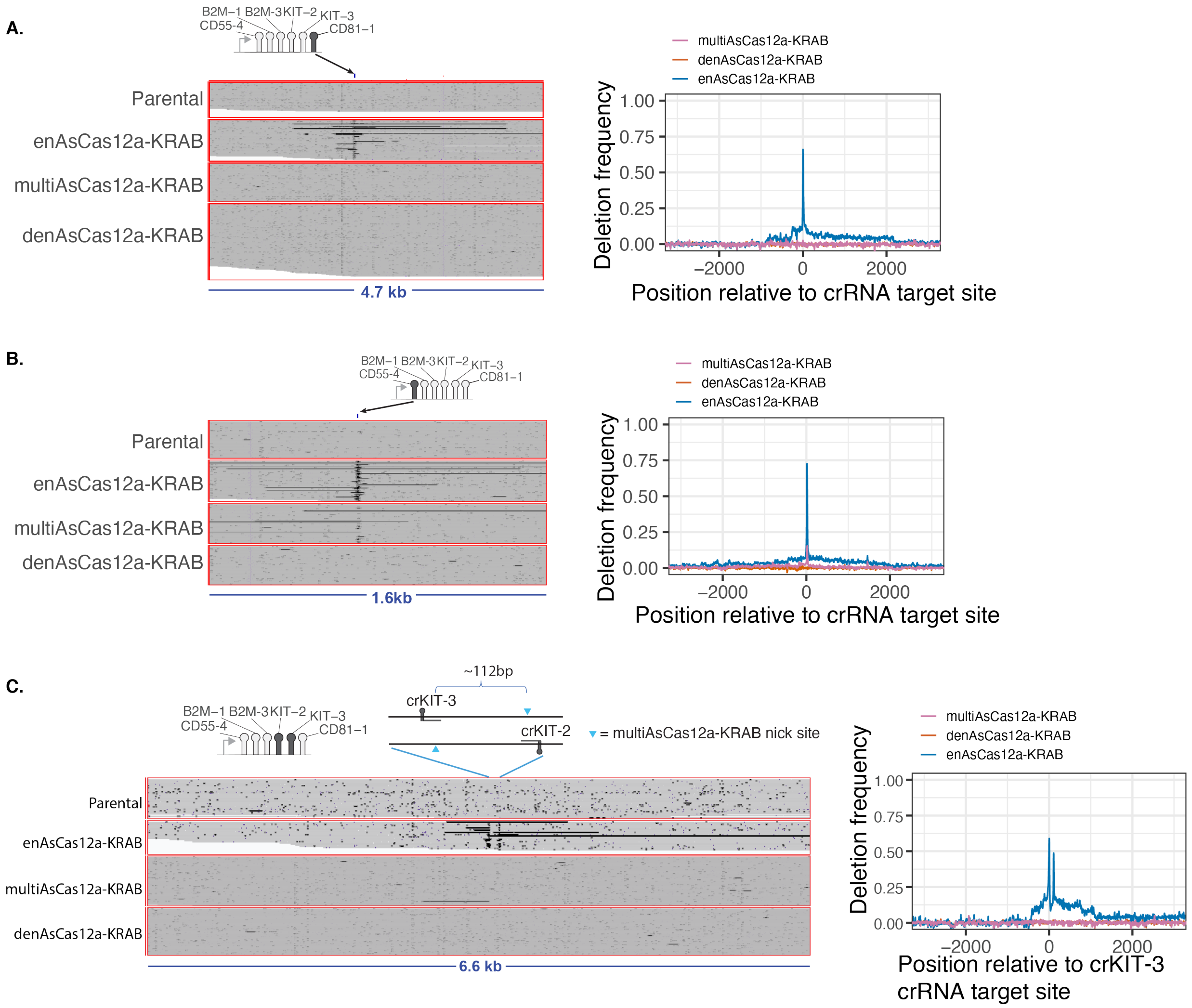

Figure S16 – Long-read Nanopore sequencing quantification of deletions surrounding single and dual crRNA target sites.

A) K562 cells piggyBAC-engineered to constitutively express the indicated fusion protein constructs were lentivirally transduced with the indicated 6-plex crRNA constructs at MOI < 0.13, sorted for transduced cells based on GFP reporter fluorescence, and the genomic DNA harvested 16 days later and subjected to long-read Nanopore sequencing. Left: Representative view of individual reads aligned to the region surrounding the crCD81-1 target site is shown, with deletions indicated by black horizontal lines. Right: Quantification of deletion frequency across the region surrounding the crCD81-1 target site.

B) Analogous to A), but shown for region surrounding the crCD55-4 target site.

C Analogous to A), but shown for region surrounding the crKIT-3 and crKIT-2 target sites located on opposing DNA strands spaced 112bp apart.

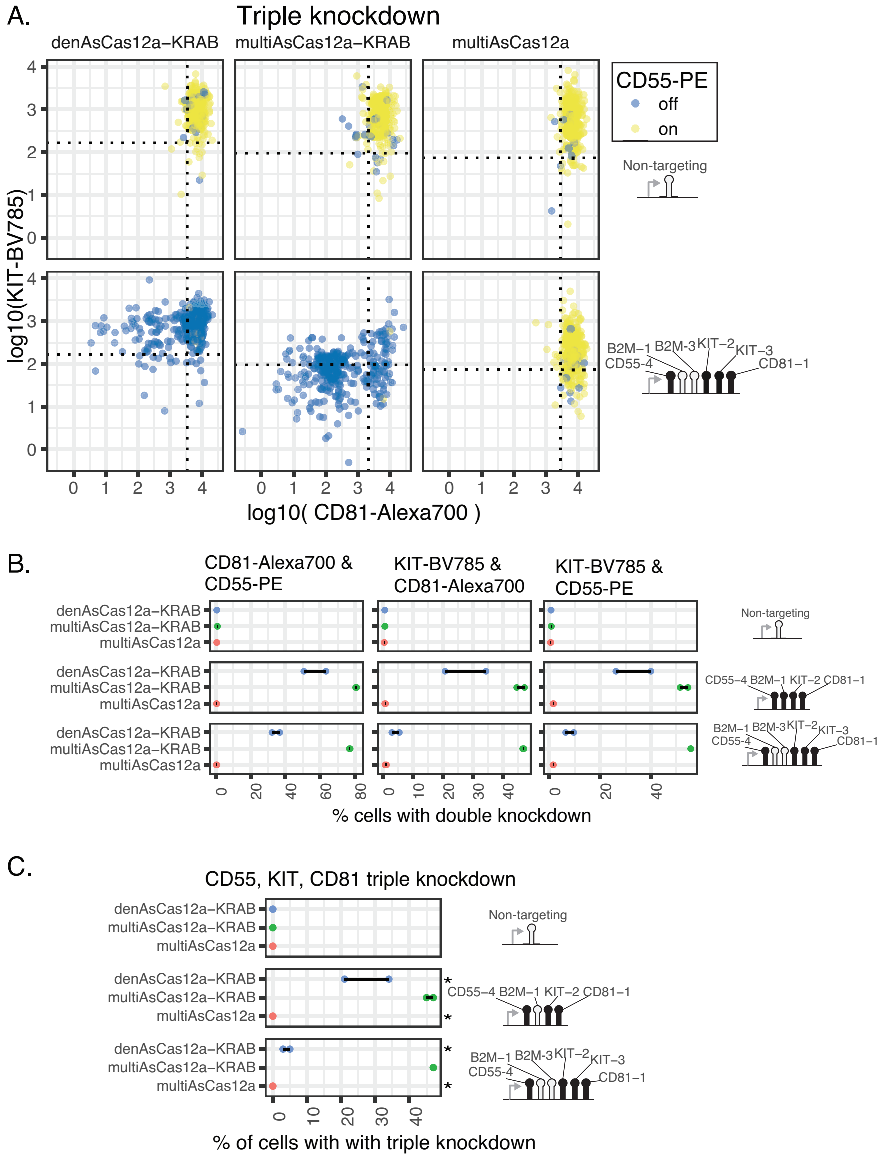

Figure S17 – Double and triple gene knockdown by CRISPRi using higher-order crRNA arrays.

A) Single-cell view of CD81, KIT, and CD55 3-way knockdown using a 6-plex crRNA construct in K562 cells piggyBac-engineered to constitutively express each of the indicated fusion protein constructs, measured by multiplexed flow cytometry.

B) Quantification of the fraction of cells showing double knockdown of pairs of target genes in the experiment described in A for 4-plex and 6-plex crRNA arrays. Double knockdown is defined as the fraction of cells with expression below the 5th percentile for the non-targeting crRNA for the expression of a given pair of target genes.

C) Triple knockdown of CD55, KIT, and CD81 from experiment described in A was quantified by the percentage of cells that are below the 5th percentile along all 3 dimensions on day 33 after transduction of crRNA constructs.

B-C) 2 biological replicates are shown as individual data points and the range is indicated by horizontal line. Two-sample chi-square test was used to compare the proportion of cells with double-knockdown between multiAsCas12a-KRAB and each of the other fusion protein constructs; asterisk indicates $p<0.01$ for all replicates (N>200 cells per replicate).

A.

Library 1 (single-plex)	TSS-targeting constructs	Negctrl-intergenic	Negctrl-unmapped
# constructs	77387	524	445
# genes targeted	559		
**For constructs with ≥1 RPM and no CRISPick Off-Target Tier I Match Bin I Matches**			
# constructs	76863	524	445
# TSS-targeting constructs targeting TTTV PAM	3357		
# target genes with >0 crRNA with lower fitness score than 1%tile of neg controls	452		
# target genes shared with prior dCas9 CRISPRi TSS tiling screen (Nunez et al., 2021)	240		

B.

Library 2 (6-plex)	Sublibrary A (positional shuffling of TSS-targeting + additional neg. controls)	Neg-ctrl only arrays (shared with Sublibrary B)
# constructs	42600	2000
**For constructs with ≥1 RPM**		
# constructs	32282	324
# constructs with TSS-targeting spacer in test position	22063	
# constructs with neg control spacer in test position	10219	
# constructs with TSS-targeting spacer in test position with lower fitness score than 1%tile of neg controls in Library 1 screen	2391	
# unique TSS-targeting spacers in test position with lower fitness score than 1%tile of neg controls in Library 1 screen	99	
# unique neg control spacers in test position	506	

C.

Library 2 (6-plex)	Sublibrary B (MYC locus)		Neg-ctrl only arrays
# constructs	6370		2000
**For constructs with ≥1 RPM**			
# constructs	1629		324
**Promoter-targeting**	**Enhancer-targeting (≥1 for each listed)**	**# constructs**	
≥1 promoter targeting spacer	none	48	
none	none	197	
≥1 promoter targeting spacer	e1	187	
none	e1	65	
≥1 promoter targeting spacer	e2	108	
none	e2	50	
≥1 promoter targeting spacer	e3	90	
none	e3	33	
≥1 promoter targeting spacer	e1e2	216	
none	e1e2	164	
≥1 promoter targeting spacer	e1e3	161	
none	e1e3	121	
≥1 promoter targeting spacer	e2e3	84	
none	e2e3	90	
≥1 promoter targeting spacer	e1e2e3	64	
none	e1e2e3	145	

Figure S18 – Summaries of Library 1 and Library 2 screens.
A) Summary of crRNA constructs in the Library 1 screen.
B) Summary of crRNA constructs in the Library 2, Sublibrary A screen. Negative control-only arrays constitute the intersect with Sublibrary B.
C) Summary of crRNA constructs in the Library 2, Sublibrary B screen. Negative control-only arrays constitute the intersect with Sublibrary A.

A.

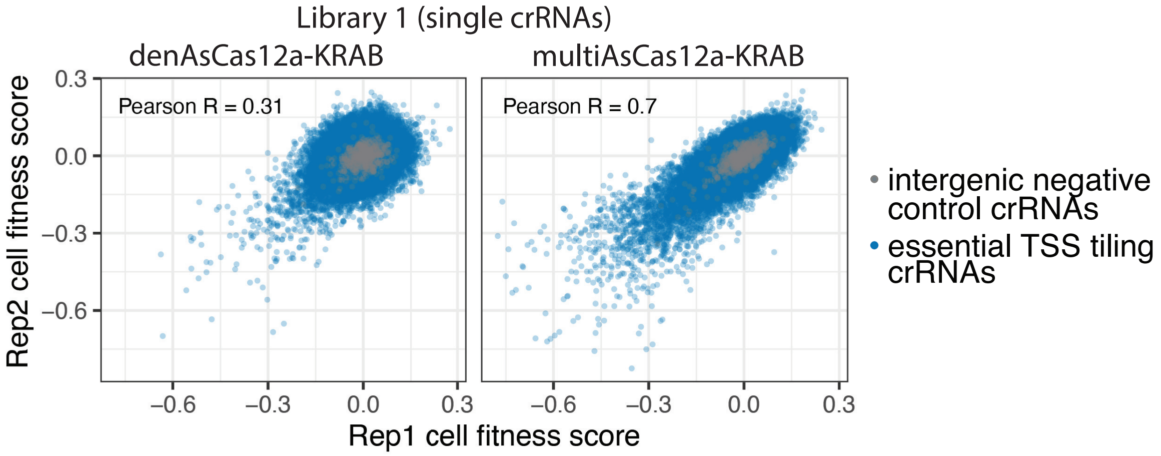

B.

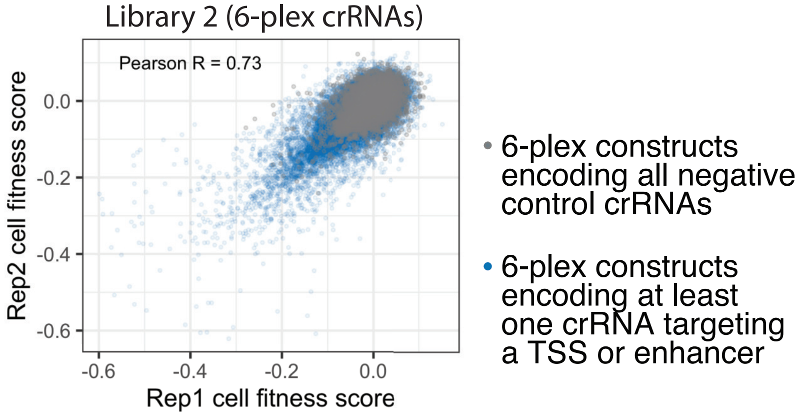

Figure S19 – **Screen replicate concordance for Library 1 and Library 2.** Shown are 2D density plots of cell fitness scores for individual crRNA constructs in **A)** Library 1 (crRNAs targeting essential TSS's in blue) and **B)** Library 2 (Sublibrary A and Sublibrary B in blue). Pearson correlation coefficients are shown for all elements in each graph excluding the intergenic negative control crRNAs (grey).

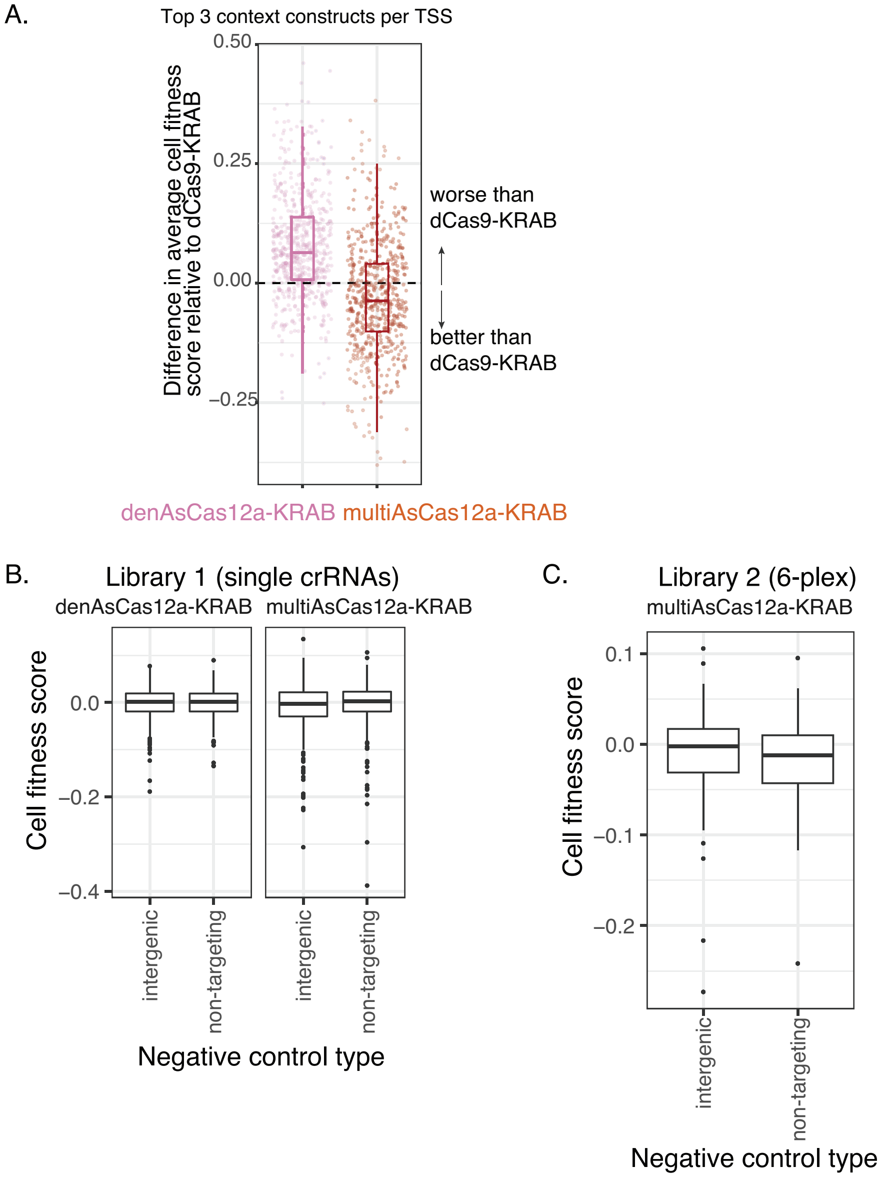

Figure S20 – Cell fitness score distributions of top multiAsCas12a-KRAB vs. dCas9-KRAB TSS-targeting guides and negative control crRNAs from pooled screens.

A) Library 1: Boxplots of average cell fitness scores of top 3 crRNAs for each essential TSS for multiAsCas12a-KRAB or denCas12a-KRAB, subtracted by the average cell fitness scores from top 3 sgRNAs for the same TSS for dCas9-KRAB. The top 3 sgRNA's per TSS for dCas9-KRAB are taken from a prior genome-wide screen (Horlbeck et al., 2016) that used 10 sgRNAs per TSS, which were pre-selected based on bioinformatic prediction of strong sgRNA activity. Boxplots show median, interquartile range, whiskers indicating 1.5x interquartile range, and are overlaid with individual data points (N = 582 TSS regions).

B) Library 1: Boxplots of cell fitness scores for the intergenic (N = 1,048 constructs) vs. non-targeting (N = 890) negative control crRNAs.

C) Library 2 6-plex constructs, categorized by whether the construct encodes exclusively intergenic (N = 1,164 constructs) vs. non-targeting (N = 780 constructs) negative control crRNAs.

A-C) Cell fitness score for each crRNA construct is averaged from two screen replicates. All boxplots display median, interquartile range, whiskers indicating 1.5x interquartile range, and outliers.

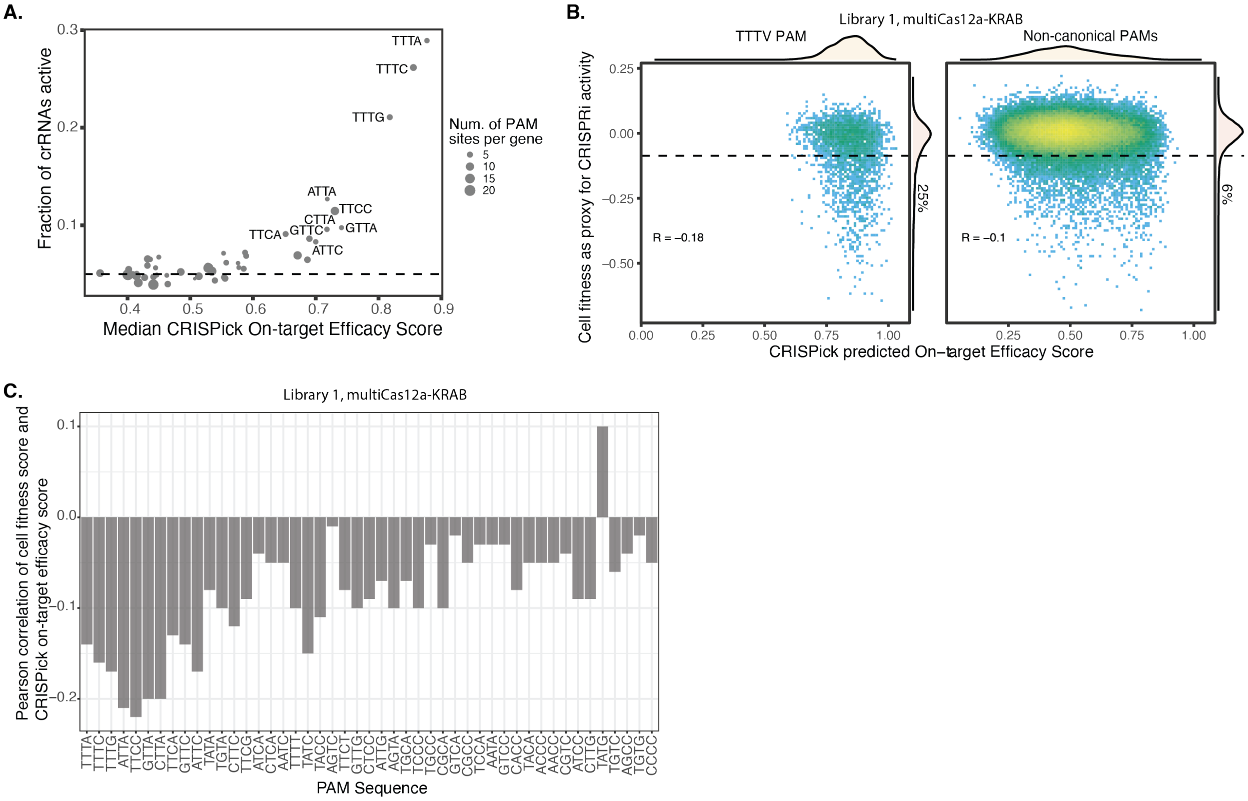

Figure S21 – Relationship among CRISPick predictions, PAM sequence, and empirical CRISPRi activity.

A) Library 1: For each of the canonical TTTV PAMs and 44 non-canonical PAMs, the fraction of crRNAs whose cell fitness scores are lower than the 5th percentile of negative controls is plotted against the median CRISPick on-target efficacy score for that PAM. Size of the bubble indicates the number of PAM sites per gene.

B) Library 1: 2D density plots of cell fitness scores of individual crRNAs for multiAsCas12a-KRAB vs. predicted crRNA on-targeting efficacy score from the CRISPick algorithm, grouped by TTTV PAM vs. non-canonical PAM's. The 5th percentiles of intergenic negative control crRNAs cell fitness scores are shown as a dashed horizontal line and the percentage of crRNAs below that threshold shown in the marginal histogram. Pearson correlation coefficients are shown.

C) Library 1: Pearson correlation coefficient between the cell fitness score and the CRISPick on-target efficacy score is shown for each individual PAM sequence for multiAsCas12a-KRAB.

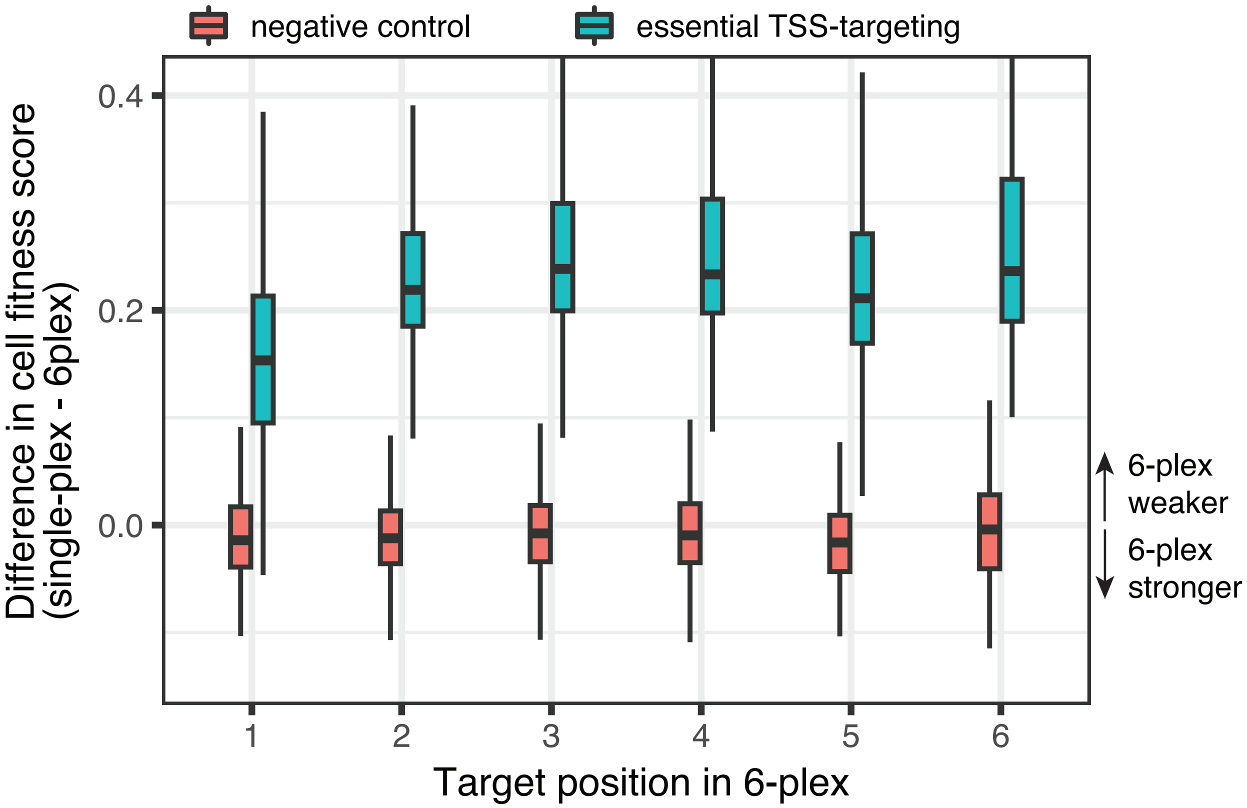

Figure S22 – Difference in cell fitness scores for 6-plex vs. 1-plex crRNA constructs from pooled screens. Library 2 Sublibrary A 6-plex crRNA cell fitness scores for a given test position spacer are compared to the cell fitness scores of the same spacer in the Library 1 (single-plex crRNA) screen. Difference in cell fitness scores (average of top 3 contexts from the 6-plex arrays minus single-plex) are shown as boxplots, which display the median, interquartile range, and whiskers indicating 1.5x interquartile range. For negative control crRNAs, N = 476, 506, 506, 506, 506, and 317 constructs in positions 1-6, respectively. For targeting crRNAs, N = 93, 99, 99, 99, 99, and 80 constructs, in position 1-6, respectively. Cell fitness scores were averaged from 2 screen replicates.

Supplementary Methods

1 Simulations of indel impacts on gene expression

The fraction of reads containing indels within each specified region was subjected to technical noise background subtraction by the fraction of reads containing indels observed in K562 parental cells. This background-subtracted indel allelic frequency was used to calculate per-copy deletion probabilities in Fig. S14 and Fig. S15B. For denAsCas12a-KRAB this background-subtraction can result in a small negative value and in those cases 0% is reported as per-copy deletion probability.

We simulated the impact of indels on gene expression under the assumption that gene expression changes are entirely driven by indels generated by the given AsCas12a fusion protein at the crRNA target site. A prior study reported at one Cas9 target site that the frequency of larger ($> 250bp$) indels is $\sim 20\%$ relative to the smaller ($< 250bp$) indels. This $20 : 80$ ratio of unobserved-to-observed indels is very likely a high overestimate in our case because our PCR amplicons are $340bp - 382bp$ and thus are expected to capture a large fraction of even the $> 250bp$ indels. Nevertheless, we added an additional 20% to the observed indel frequencies to arrive at our final estimates of probability of the occurrence of any $> 1bp$ indels per DNA copy for a single target site. Based on this indel probability, we calculated the proportion of cell population expected to harbor a given number of DNA copies with indels assuming indels occur independently among DNA copies and using previously measured DNA copy number for each genomic locus (Zhou et al., 2019). For dual targeting of the KIT locus by crKIT-2 and crKIT-3, we assume that the occurrence of a large ($> 250bp$) unobserved deletion at one target site precludes a deletion at the other target site.

Starting from the single-cell expression distributions obtained by flow cytometry from the non-targeting crRNA control, we simulated the expected change in single-cell expression distribution under the assumption that any $> 1bp$ indel in the PCR amplicon would generate a null allele completely abolishing expression of the target gene in cis (i.e., indel in 1 out of 3 copies would reduce expression by 33%). We refer to this as the "expected null" expression change, which is expected to be a high overestimate of impact on expression. To better more accurately estimate the true hypomorphic effects of indels, we calculated a "hypomorphic coefficient" defined as the ratio of the observed median expression change vs. the expected null expression change for opAsCas12a. We multiply the expected null expression change for all other fusion proteins by this hypomorphic coefficient to derive an "expected hypomorph" expression change for each fusion protein and crRNA construct combination.

2 Description of Library 2 screen read mapping algorithm for 6-plex crRNA constructs

First, reference construct sequences were created by interspersing provided spacer and constant regions. Each construct is then given a unique construct id (`CID`). Each `CID` is then split into `R1` and `R2` reference sequences, which are constructed by taking the first three and last three spacer-construct pairs of the reference sequence respectively. The `R2` sequence is then reverse complemented for matching against the `R2` sequencing reads.

Next, two hashmaps are created for the `R1` and `R2` spacer-construct pairs respectively, which map the `R1`/`R2` sequences to a set of corresponding `CID`s, requiring perfect sequence matches.

Finally, for each `R1`/`R2` sequencing pair, each k-mer ($k =$ length of `R1`/`R2` respective construct sequence) in the sequence is mapped against their respective `R1`/`R2` hashmap. If both sequencing pairs are able to be mapped to a `CID` set, then the intersection of their sets is their original construct, and the total count of that `CID` is incremented.

The above algorithm is implemented as the `casmap constructs` command in a package written in Rust, available at `https://github.com/noamteyssier/casmap`.

3 Golden Gate assembly and bacterial transformation for pooled crRNA library cloning

The Golden Gate Assembly reaction was carried out in a $100\mu l$ reaction containing $2.5U$ Esp3I (Thermo ER0452) and $1000U$ T4 DNA Ligase in T4 DNA Ligase reaction buffer (NEB M0202L). The reaction mix was incubated for 31 cycles alternating between $37°C$ and $16°C$ for $20min$ at each temperature, then heat-inactivated at $65°C$ for $5min$. Assembly reactions were column purified with Zymo DNA clean and concentrator-5 (Zymo D4004), eluted in $12\mu l$ of water and $< 7\mu l$ added to $70\mu l$ of MegaX DH10B T1R Electrocomp Cells (C640003) for electroporation using BioRad Gene Pulser Xcell Electroporator with settings $2.0kV$, $200ohms$, $25\mu F$. Cells were recovered at $37°C$ for rotating for $1h$ in $\sim 5ml$ recovery media from the MegaX DH10B T1R Electrocomp Cells kit and small volumes plated onto bacterial LB plates containing carbenicillin for quantification of colony forming units. The remaining recovery culture was inoculated directly into $200ml$ liquid LB media with carbenicillin and incubated in $37°C$ shaker for $12h - 16h$ prior to harvesting for plasmid purification using ZymoPURE II Plasmid Midiprep kit (Zymo D4200). The colony forming units from the small volumes in the bacterial plates were used to estimate coverage of the plasmid

library. 24 individual colonies were verified by Sanger sequencing to check for any gross abnormalities in cloning, and the library subjected to library preparation and Illumina sequencing as described in Methods.

4 Bioinformatics processing of indel quantificaition by short-read sequencing of PCR amplicons

CRISPResso2 was run as follows, here shown for the crCD55-4 spacer sequence ("reference sequence" refers to the PCR amplicon sequence provided in Supplementary Table 2):

```
    CRISPResso --fastq\_r1 R1.fastq.gz --fastq\_r2 R2.fastq.gz --amplicon\_seq [reference sequence] -g
     ACTGGTATTGCGGAGCCACGAGG -wc -3 -w 12
```

For crRNA constructs in which the PAM is found on the opposite strand with respect to the amplicon sequence (in this case, CD81) the following modifications were included:

```
CRISPResso --fastq_r1 pCH45H-CD81-1_S4_L001_R1_001.fastq.gz --fastq_r2 pCH45H-CD81-1_S4_L001_R2_001.fastq.gz --
     amplicon_seq [reference sequence] -p 3 -g gagaccttcctgggggtcgcgcc -wc -18 -w 20
```

Quantification diagrams were generated in R.

For analysis at the KIT locus, cells were lysed using QuickExtract DNA Solution (Lucigen) and amplicons were generated using 15 cycles of PCR to introduce Illumina sequencing primer binding sites and 0-8 staggered bases to ensure library diversity. After reaction clean-up using ExoSAP-IT kit (Thermo Fisher 78201), an additional 15 cycles of PCR was used to introduce unique dual indices and Illumina P5 and P7 adaptors. Libraries were pooled and purified by SPRIselect magnetic beads before paired-end sequencing using an Illumina MiSeq at the Arc Institute Multi-omics Technology Center. Sequencing primer binding sites, unique dual indices (from Illumina TruSeq kits), P5 and P7 adaptor sequences are from Illumina Adaptor Sequences Document # 1000000002694 v16.

Reads were analyzed using CRISPRessoBatch from CRISPResso2 117 as follows:

```
CRISPRessoBatch --batch_settings batch2.batch --amplicon_seq [reference sequence] -p 3 -g
     TCTGCGTTCTGCTCCTACTGCTT -wc -4 -w 15
```

For dual gRNA cutting, both guides were included in the batch analysis.The total number of insertions and deletions at each amplicon position were calculated and displayed using the `effect_vector_combined.txt` output.

5 Analysis of 3' RNA-seq data

For generating scatter plots, `normTransform` function from DESeq2 v1.34 (Love et al., 2014) was used to normalize the raw counts and then a pseudocount of 1 was added, and log2-transformed. The output was plotted in R as scatter plots. For differential expression analysis, DESeq2 default Wald test was used to compare each targeting construct (one replicate) with non-targeting samples (two replicates). We calculated log2 transformed TPM counts and applied the threshold of 6.5 to eliminate genes with low expression. Using `ggplot2`, volcano plots visualized in R are then displayed for genes with `log2FoldChange` above or below 2.055 and p-values smaller than 0.01. The `log2FoldChange` cutoff was based on visually examining the concordance between two replicates of untransduced controls and manually identifying a threshold below which the `log2FoldChange` are poorly correlated between the replicates of the untransduced control.

To evaluate potential off-target effect of spacers, we used crisprVerse v1.0.0 and crisprBowtie v1.2.0 (Hoberecht et al., 2022) together with other R packages including GenomicRanges (version 1.50) and tidyverse (version 1.3.2). First, we defined a dictionary of spacers as follows:

"TCCTCCAGCATCTTCCACATTCA":"HBG-2"
"TTCTTCATCCCTAGCCAGCCGCC":"HBG-3"
"CTTAGAAGGTTACACAGAACCAG":"HS2-1"
"TGTGTAACCTTCTAAGCAAACCT":"HS2-2"
"AGGTGGAGTTTTAGTCAGGTGGT":"HS2-3"
"ATTAACTGATGCGTAAGGAGATC":"NT-3"

Then, the `runCrisprBowtie` function was used with these parameters: `crisprNuclease` as `enAsCas12a`, `n_mismatches` equal 3, `canonical` equal FALSE, and `bowtie_index` as a path to folder including pre-indexed hg38 reference genome. Thus, the results from this step allow us to assess our previously designed spacers and annotate potential off-target loci in the human genome. To annotate results, we used reference annotation GENCODE (version 34) and we defined pam_site +/- 2500 bp for each predicted off-target to overlap them with matched transcription start sites (TSS) +/- 1000 bp of all annotated genes. Results shown as annotated tables.

6 Nanopore long-read sequencing analysis of deletion frequencies

Except where specified below, reagents are from the Nanopore Ligation Sequencing Kit V14 (`SQK-LSK114`). To summarize in brief, synthetic spCas9 Alt-R crRNAs targeting ∼15-20kb regions surrounding Cas12a crRNA target sites were designed according to instructions for the Nanopore Cas9 Sequencing Kit and ordered from IDT. At least two Cas9 guides were used to target the (+) strand upstream of the Cas12a crRNA target sites, and at least two Cas9 guides were used to target the (-) strand downstream of the target sites. Synthetic spCas9 guides were pooled and annealed with spCas9 tracrRNA (IDT Cat # 1072532) by heating to 95°C for 5 minutes and cooling on the bench at room temperature, followed by assembly into ribonucleoprotein complexes by incubating the following mixture at room temperature for 30 minutes:

- 10 μl annealed crRNA:tracrRNA pool (100μM),
- 10 μl 10x NEB rCutSmart Buffer (NEB B6004S),
- 79.2 μl nuclease-free water, and
- 0.8 μl HiFi Cas9 (62μM, *S. pyogenes* HiFi Cas9 nuclease V3 IDT Cat#1081060).

5 μg of genomic DNA was dephosphorylated in a 30 μl reaction volume using 15U of Quick calf intestinal phosphatase (NEB cat #M0525) and 3 μl of 10x rCutsmart Buffer (NEB B6004S), incubated at 37°C for 10 minutes, 80°C for 2 minutes, and held at 20°C. The entire volume of dephosphorylated genomic DNA is then mixed with 10 μl of Cas9 ribonucleoprotein, 1 μl 10mM dATP, and 1 μl Taq polymerase for a total of 42 μl, incubated at 37°C for 20 minutes, 72 °C for 5 minutes, and held at 4°C. The reaction is then cleaned up using 21 μl (0.5X) AMPure XP using two washes with 200 μl 80% ethanol, and eluted in 61 μl of water.

Adapters ligation was set up as follows:

- 60 μl DNA eluate,
- 25 μl Ligation Buffer,
- 10 μl NEBNExt Quick T4 DNA Ligase (NEB E6056S), and
- 5 μl Ligation Adapter,

and incubated for 40 minutes at room temperature. The entire ligation reaction was subjected to AMPure XP bead clean up using 40 μl (0.4X) of AMPure beads, two washes with Long Fragment Buffer, and eluted in 15 μl Elution Buffer. Size distribution and concentration of library was checked by Agilent Tapestation Genomic DNA ScreenTape Analysis. ∼75ng-560ng of each library per flow cell was each run on 1 or 2 flow cells on a Promethion P2 Solo, yielding ∼100k reads per flow cell run over 40h-72h.

References

Hoberecht, L., Perampalam, P., Lun, A. and Fortin, J.-P. (2022). A comprehensive Bioconductor ecosystem for the design of CRISPR guide RNAs across nucleases and technologies. Nat. Commun. *13*, 6568.

Horlbeck, M. A., Gilbert, L. A., Villalta, J. E., Adamson, B., Pak, R. A., Chen, Y., Fields, A. P., Park, C. Y., Corn, J. E., Kampmann, M. and Weissman, J. S. (2016). Compact and highly active next-generation libraries for CRISPR-mediated gene repression and activation. Elife *5*.

Love, M. I., Huber, W. and Anders, S. (2014). Moderated estimation of fold change and dispersion for RNA-seq data with DESeq2. Genome Biol. *15*, 550.

Zhou, B., Ho, S. S., Greer, S. U., Zhu, X., Bell, J. M., Arthur, J. G., Spies, N., Zhang, X., Byeon, S., Pattni, R., Ben-Efraim, N., Haney, M. S., Haraksingh, R. R., Song, G., Ji, H. P., Perrin, D., Wong, W. H., Abyzov, A. and Urban, A. E. (2019). Comprehensive, integrated, and phased whole-genome analysis of the primary ENCODE cell line K562. Genome Res. *29*, 472–484.
