# Supplementary material for: Engineered CRISPR-Cas12a for higher-order combinatorial chromatin perturbations
Source: Nat Biotechnol. 2024 May 17;43(3):369–83. doi: 10.1038/s41587-024-02224-0 (PMC11919711; doi:10.1038/s41587-024-02224-0)
Supplement: Supplementary file 2 — Reporting Summary [file 41587_2024_2224_MOESM2_ESM.pdf]

nature portfolio

Corresponding author(s): Luke Gilbert

Last updated by author(s): Mar 26, 2024

Reporting Summary

Nature Portfolio wishes to improve the reproducibility of the work that we publish. This form provides structure for consistency and transparency in reporting. For further information on Nature Portfolio policies, see our Editorial Policies and the Editorial Policy Checklist.

Statistics

For all statistical analyses, confirm that the following items are present in the figure legend, table legend, main text, or Methods section.

n/a	Confirmed	
☐	☒	The exact sample size (*n*) for each experimental group/condition, given as a discrete number and unit of measurement
☐	☒	A statement on whether measurements were taken from distinct samples or whether the same sample was measured repeatedly
☐	☒	The statistical test(s) used AND whether they are one- or two-sided<br>*Only common tests should be described solely by name; describe more complex techniques in the Methods section.*
☒	☐	A description of all covariates tested
☒	☐	A description of any assumptions or corrections, such as tests of normality and adjustment for multiple comparisons
☐	☒	A full description of the statistical parameters including central tendency (e.g. means) or other basic estimates (e.g. regression coefficient) AND variation (e.g. standard deviation) or associated estimates of uncertainty (e.g. confidence intervals)
☐	☒	For null hypothesis testing, the test statistic (e.g. *F*, *t*, *r*) with confidence intervals, effect sizes, degrees of freedom and *P* value noted<br>*Give P values as exact values whenever suitable.*
☒	☐	For Bayesian analysis, information on the choice of priors and Markov chain Monte Carlo settings
☒	☐	For hierarchical and complex designs, identification of the appropriate level for tests and full reporting of outcomes
☐	☒	Estimates of effect sizes (e.g. Cohen's *d*, Pearson's *r*), indicating how they were calculated

Our web collection on statistics for biologists contains articles on many of the points above.

Software and code

Policy information about availability of computer code

Data collection	No specialized software or custom code was used for data collection.
Data analysis	Code for read counting for single-plex screens is available at https://noamteyssier.github.io/sgcount/. Code for read counting for 6-plex screens is available at https://github.com/noamteyssier/casmap. R scripts for downstream screen analyses are deposited on Zenodo (DOI: 10.5281/zenodo.10784378) and incorporates functions available as an R package at https://github.com/chris-hsiung/bears01.

For manuscripts utilizing custom algorithms or software that are central to the research but not yet described in published literature, software must be made available to editors and reviewers. We strongly encourage code deposition in a community repository (e.g. GitHub). See the Nature Portfolio guidelines for submitting code & software for further information.

Data

Policy information about availability of data

All manuscripts must include a data availability statement. This statement should provide the following information, where applicable:

- Accession codes, unique identifiers, or web links for publicly available datasets
- A description of any restrictions on data availability
- For clinical datasets or third party data, please ensure that the statement adheres to our policy

Guide sequences, read counts and processed data for all screens, primer sequences, and plasmid sequences are included as supplementary files.

Human research participants

Policy information about studies involving human research participants and Sex and Gender in Research.

Reporting on sex and gender	N/A
Population characteristics	N/A
Recruitment	N/A
Ethics oversight	N/A

Note that full information on the approval of the study protocol must also be provided in the manuscript.

Field-specific reporting

Please select the one below that is the best fit for your research. If you are not sure, read the appropriate sections before making your selection.

☒ Life sciences ☐ Behavioural & social sciences ☐ Ecological, evolutionary & environmental sciences

For a reference copy of the document with all sections, see nature.com/documents/nr-reporting-summary-flat.pdf

Life sciences study design

All studies must disclose on these points even when the disclosure is negative.

Sample size	All screens were performed in duplicate per standards in the functional genomics field. Correlation between samples demonstrate adequate sample size.
Data exclusions	Exclusions of constructs in screen analyses are described in Methods based on minimum RPM > 1. Both Library 1 and Library 2 were constructed from pooled oligonucleotide libraries designed to contain crRNA constructs designed for exploratory analysis for a separate unpublished study. Sequencing reads from those non-contributory constructs are present in the raw fastq files, do not affect interpretation of Library 1 and Library 2 screen cell fitness scores, and are excluded from analysis in the present study.
Replication	Number of replicates are specified in figure captions.
Randomization	Populations of cells are randomly allocated into individual wells for treatments.
Blinding	Not used. This is not applicable to molecular biology and functional genomics experiments. The analyses entail interpretations of quantitative distributions and do not involve subjective judgment calls by the experimenter.

Reporting for specific materials, systems and methods

We require information from authors about some types of materials, experimental systems and methods used in many studies. Here, indicate whether each material, system or method listed is relevant to your study. If you are not sure if a list item applies to your research, read the appropriate section before selecting a response.

Materials & experimental systems

n/a	Involved in the study	
☐	☒	Antibodies
☐	☒	Eukaryotic cell lines
☒	☐	Palaeontology and archaeology
☒	☐	Animals and other organisms
☒	☐	Clinical data
☒	☐	Dual use research of concern

Methods

n/a	Involved in the study	
☒	☐	ChIP-seq
☐	☒	Flow cytometry
☒	☐	MRI-based neuroimaging

Antibodies

Antibodies used	The following antibodies were used for flow cytometry at 1:100 dilution: CD55-APC (Biolegend 311312), CD55-PE (Biolegend 311308), CD81-PE (Biolegend 349506), CD81-AlexaFluor700 (Biolegend 349518), B2M-APC (Biolegend 316311), KIT-PE (Biolegend 313204), KIT-BrilliantViolet785 (Biolegend 313238), FOLH1-APC (Biolegend 342508). The following antibodies were used for western

	blotting: anti-HA-tag rabbit antibody (Cell Signaling Technology, cat# 3724S) at 1:1000 dilution and anti-GAPDH rabbit antibody (Cell Signaling Technology, cat# 2118) at 1:3000 dilution.
Validation	Antibody specificity was directly validated by expression knockdown in CRISPRi experiments. Western blot antibodies were validated by migration of band at the expected size and/or the negative control samples that do not express the transgenic epitope targeted by the antibody (e.g. anti-HA).

Eukaryotic cell lines

Policy information about cell lines and Sex and Gender in Research

Cell line source(s)	K562 and HEK 293T cells were from ATCC. C42B cells are a gift from Leland Chung lab (the lab that originally derived the cell line). RN2 cell line was described previously (Zuber, J et al. Nature Biotechnology 29, 79-83, 2011) and was a gift from the laboratory of Christopher Vakoc (CSHL) to Junwei Shi's laboratory. B16-F10 cell line was obtained from ATCC (CRL-6475).
Authentication	STR authentication was used to determine the identity of the parental cell lines used.
Mycoplasma contamination	Mycoplasma screening was conducted on a routine basis and were always negative.
Commonly misidentified lines (See ICLAC register)	N/A

Flow Cytometry

Plots

Confirm that:

☒ The axis labels state the marker and fluorochrome used (e.g. CD4-FITC).

☒ The axis scales are clearly visible. Include numbers along axes only for bottom left plot of group (a 'group' is an analysis of identical markers).

☒ All plots are contour plots with outliers or pseudocolor plots.

☒ A numerical value for number of cells or percentage (with statistics) is provided.

Methodology

Sample preparation	K562 cell suspensions were transferred into 96-well plates. C42B and HEK 293T cells are harvested by trypsinization with 0.25% Trypsin-EDTA for 5min at 37deg. C and then neutralized with 2x volumes of media, then transferred into 96-well plates. Washes, antibody staining, and data acquisition were performed using PBS 1% BSA (including 1:100 dilution of antibody for staining step) as described in detail in the Methods section.
Instrument	BD FACS Aria Fusion sorter. Attune NxT flow cytometer.
Software	For analysis, .fcs files were exported and populations gated in FlowJo (10.8.2). Data exported as .csv files and plotted using custom R (version 4.1.0) scripts
Cell population abundance	>5,000 cells were sorted for cell line engineering involving K562 cells. For C42B cell line engineering, >200 cells were sorted. Post-sort purity for sorting analyses were >90%, as determined by analyzing a small aliquot of a representative sorted sample to determine the fraction of cells that fall into the original sorting gate.
Gating strategy	Single cells are gated based on FSC/SSC, followed by gating of specific fluorescent markers for crRNA constructs using the 99.9%tile of untransduced controls as threshold.

☒ Tick this box to confirm that a figure exemplifying the gating strategy is provided in the Supplementary Information.
